# Nanoscale goldbeating: Solid-state transformation of 0D and 1D gold nanoparticles to anisotropic 2D morphologies

**DOI:** 10.1093/pnasnexus/pgad267

**Published:** 2023-08-18

**Authors:** Md Rubayat-E Tanjil, Tanuj Gupta, Matthew T Gole, Keegan P Suero, Zhewen Yin, Donald J McCleeary, Ossie R T Douglas, Maegen M Kincanon, Nicholas G Rudawski, Alissa B Anderson, Catherine J Murphy, Huijuan Zhao, Michael Cai Wang

**Affiliations:** Department of Mechanical Engineering, University of South Florida, Tampa, FL 33620, USA; Department of Mechanical Engineering, Clemson University, Clemson, SC 29634-0921, USA; Department of Chemistry, University of Illinois at Urbana-Champaign, Urbana, IL 61801, USA; Department of Mechanical Engineering, University of South Florida, Tampa, FL 33620, USA; Department of Mechanical Engineering, University of South Florida, Tampa, FL 33620, USA; Department of Mechanical Engineering, University of South Florida, Tampa, FL 33620, USA; Department of Mechanical Engineering, University of South Florida, Tampa, FL 33620, USA; Department of Chemistry, University of Illinois at Urbana-Champaign, Urbana, IL 61801, USA; Herbert Wertheim College of Engineering Research Service Centers, University of Florida, Gainesville, FL 32611, USA; Department of Chemistry, University of South Florida, Tampa, FL 33620, USA; Department of Chemistry, University of Illinois at Urbana-Champaign, Urbana, IL 61801, USA; Department of Mechanical Engineering, Clemson University, Clemson, SC 29634-0921, USA; Department of Mechanical Engineering, University of South Florida, Tampa, FL 33620, USA; Department of Medical Engineering, University of South Florida, Tampa, FL 33620, USA; Department of Chemical, Biological, and Materials Engineering, University of South Florida, Tampa, FL 33620, USA

**Keywords:** nanoparticle deformation, high-pressure compression, molecular dynamics

## Abstract

Goldbeating is the ancient craft of thinning bulk gold (Au) into gossamer leaves. Pioneered by ancient Egyptian craftsmen, modern mechanized iterations of this technique can fabricate sheets as thin as ∼100 nm. We take inspiration from this millennia-old craft and adapt it to the nanoscale regime, using colloidally synthesized 0D/1D Au nanoparticles (AuNPs) as highly ductile and malleable nanoscopic Au ingots and subjecting them to solid-state, uniaxial compression. The applied stress induces anisotropic morphological transformation of AuNPs into 2D leaf form and elucidates insights into metal nanocrystal deformation at the extreme length scales. The induced 2D morphology is found to be dependent on the precursor 0D/1D NP morphology, size (0D nanosphere diameter and 1D nanorod diameter and length), and their on-substrate arrangement (e.g., interparticle separation and packing order) prior to compression. Overall, this versatile and generalizable solid-state compression technique enables new pathways to synthesize and investigate the anisotropic morphological transformation of arbitrary NPs and their resultant emergent phenomena.

**Significance Statement**

Inspired by the ancient craft of mechanically beating bulk gold ingots into ultrathin leaves/sheets, we adopt this technique at the nanoscale regime, using gold nanocrystals to gain insights into the deformation of metallic nanoparticles at extreme length scales. Our uniaxial compression technique transforms arbitrary 0D and 1D metal nanoparticles into anisotropic 2D leaf-like morphologies, which are dependent on the precursor nanoparticles' original morphology, size, and particle arrangement. This opens new routes toward understanding anisotropic 2D transformation and the concomitant emergent material properties, generalizable to arbitrary nanocrystals.

## Introduction

The process of thinning bulk gold (Au) into gossamer, leaf-like sheets—known as goldbeating—is an ancient handicraft. Adopted by the Egyptians more than five millennia ago, the unparalleled inertness, malleability, and durability of Au uniquely enable their transformation via manual pounding from bulk ingots to thin leaf form, becoming indispensable for myriad applications in art and adornments, such as on the tomb of Thebes and Saqqara (1). As the craft evolved, the thinness of the Au leaf progressed toward the nanoscale regime from ∼300 nm in the Eighteenth Dynasty of ancient Egypt toward less than 100 nm, achievable with modern mechanized goldbeating. Despite standing the test of time, this lower limit of ∼100 nm achieved by goldbeating is still much thicker than the single-digit nanometer Au thin films achievable via modern metallization technologies such as electrochemical deposition, atomic layer deposition, vacuum-based evaporation, and molecular beam epitaxy (2). This begs the question: is it possible to goldbeat down to the few or even single-digit nanometer regime? Here, we take inspiration from this ancient craft of goldbeating and apply it at the nanoscale regime to push the thinness limit of Au leaves toward the 2D limit. Using Au nanoparticles (AuNPs) as nanoscale solid precursors in the form of 0D nanospheres (NSs) and 1D nanorods (NRs), we investigate their transformation via uniaxial compression (nanoscale goldbeating) into 2D Au leaf and study their resultant morphologies.

Au, as the canonical precious metal with superlative material properties, has been instrumental in shaping the modern world, from microelectronics to (nano)medicine (3–8). In nanoscopic form, low-dimensional 0D/1D AuNPs exhibit properties different from their “bulk” 3D counterparts, arising from reduced physical dimensionality and confinement effects (9–11). Significant advancements in the synthesis of low-dimensional Au have been made, especially in seed-mediated colloidal NP synthesis with precise control over size, shape, aspect ratio (AR), surface chemistry, and ligand distribution (12–15). Due to the minimization of net surface energy, these colloidally synthesized NPs typically exhibit intrinsic high-symmetry and faceted crystalline morphologies such as NSs, NRs, nanopolyhedrons, nanocubes, nanooctahedrons, nanostars, nanoplates, nanocages, and nanoshells (16, 17). Modifying these intrinsic symmetries in NPs introduces morphological anisotropy that can induce emergent properties such as localized surface plasmon resonance (18–21), catalysis (22), and magnetism (23, 24). Conventional bottom-up solution-based techniques, including seed-mediated colloidal synthesis (25–28), vapor deposition (29), electrochemistry, and sonochemistry (30), can yield morphologically anisotropic AuNPs. Multistep, seed-mediated techniques allow morphological control with capping layers over NP shape, sizes, and surface chemistries while achieving scalable synthesis (31, 32). Kinetically controlled geometric anisotropy can be achieved by optimizing metal precursor concentration, synthesis conditions (temperature, time), structure-directing reducing agents, additives, and surfactants (33–40).

In contrast, top-down, solid-state techniques remain underexplored as an alternative route to achieving morphological control and thus far have mainly entailed lithographic patterning (optical, imprint, focused ion beam, etc.) (41) and compression strategies (42, 43). However, lithography techniques are often limited by lithographic resolution, scalability, and poor crystallinity (44, 45). Mechanical-based (i.e. deformation) strategies offer exciting opportunities for control over NP morphology to induce anisotropy. Conventional top-down compression-based strategies primarily entail hydrostatic compression of NP superlattices, resulting in omnidirectional sintering-driven 3D mesoporous Au architectures (46, 47). Alternatively, deviatoric, uniaxial hard-contact compression (nanoscale goldbeating) can induce displacive, motion-mediated (48), anisotropic morphological transformation of metal NPs. Previous efforts on uniaxial compression of individual or superlattices of AuNPs (and other metallic NPs) have primarily focused on investigating the fabrication aspect of the nanostructured superlattices and resultant properties (49, 50).

Overall, shape anisotropy in AuNPs induced by uniaxial compression remains underexplored. Further understanding of this severe plastic deformation process akin to nanoscale goldbeating lends insights into the deformation of metal NPs at extreme length scales and the associated parameters. These anisotropic transformations into 2D Au leaf morphologies are investigated experimentally and computationally. The top-down, uniaxial compression-based nanoscale goldbeating technique demonstrated here is versatile and generalizable to other metallic, polymeric, or ceramic NPs. The observed plasticity and transformed 2D morphologies depend on the precursor AuNP size (diameter and AR) and the on-substrate assembly/interparticle separation.

## Results and discussion

**Fig. 1.**
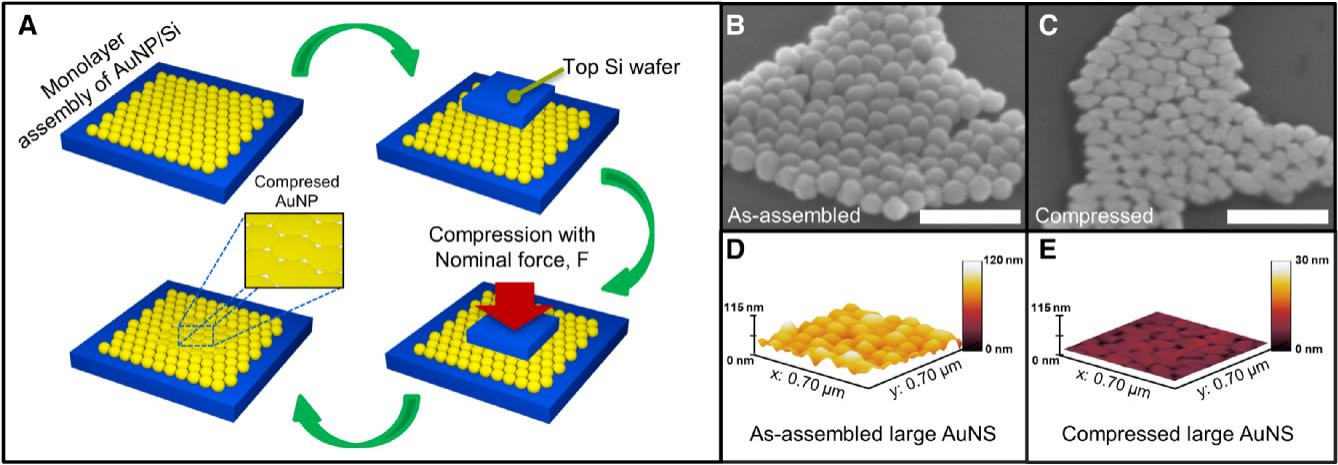
A) Schematic showing the compression of monolayer surface assembly of AuNPs on silicon substrate. The top silicon compression wafer is compressed uniaxially onto the AuNS/Si. The total lateral area of the compressed 2D Au domain equals the top compression silicon wafer dimension. SEM (50° tilted view) images of B) large AuNS (103 nm) as-assembled on the silicon substrate and C) after compression. The flattened 2D Au leaf-like morphology is evident from the SEM images. All scale bars are 500 nm. AFM height maps of D) as-assembled and E) compressed AuNS morphology, indicating the severe plastic deformation and flattening of the AuNSs due to uniaxial compression.

AuNSs and AuNRs were colloidally synthesized and subsequently functionalized with thiolated methoxyl polyethylene glycol (mPEG-SH). The hydrophilic capping polymer brushes improve AuNP colloidal stability and reduce agglomeration in solution via steric isolation, thus improving deposition and assembly uniformity on the substrate for the compression process (see Materials and methods for details). The AuNS and AuNR colloidal solutions were drop-casted and assembled in (sub)monolayers onto polished silicon substrates (2 × 2 mm^2^). A custom-built hard-contact compression setup was used to apply uniaxial compressive stress via direct contact between the top silicon wafer surface and the assembled AuNP on the bottom silicon substrate (AuNP/Si) (Fig. 1A). The degree of compression is quantified from the applied *nominal* stress (*σ*_nom_) and applied *normalized* stress (*σ*_norm_) (see Materials and methods for details).

To determine the appropriate amount of stress to apply and induce severe plastic deformation and 2D transformation of the AuNPs, we refer to the strengths of Au micro/nanocrystals documented in the existing literature (Table S1) (51–58). It is clear that significant disparities of reported yield strength exist in the literature, mainly due to the difference of particle size, shape, loading conditions, etc. Therefore, considering higher stiffness (59) and higher compression resistance, and lower density of structural defects in our colloidally synthesized AuNPs compared with bulk Au, we estimated that for our small AuNPs (∼18 nm diameter), an applied *normalized* stress of ∼3.6 GPa would be sufficient to initiate dislocation nucleation and induce severe plastic deformation to study the anisotropic 2D morphological transformation. Our empirical results indeed show that AuNPs of different sizes, morphologies, and assemblies all exhibit significant plastic deformation under ∼3.6 GPa applied uniaxial *normalized* compressive stress.

**Fig. 2.**
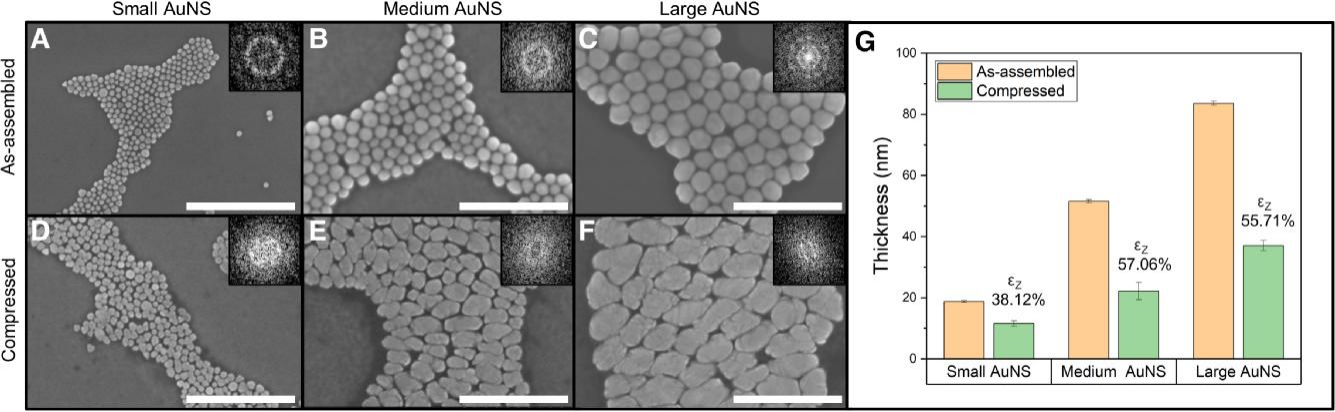
Morphological transformation of various sizes of AuNSs via uniaxial compression. SEM of as-assembled AuNSs of various sizes with 5k PEG: A) small—18 nm, B) medium—59 nm, and C) large—103 nm. The inset FFT shows diffused rings with hexagonal spots, indicative of a hexagonal closed-packed assembly of the AuNSs. SEM of transformed 2D morphology after compression of AuNSs of sizes D) small, E) medium, and F) large. The AuNS's deformation could be influenced by the stochastic crystal facet orientation of the individual particles and the number of neighboring AuNSs, leading to different deformed morphologies under the same applied *normalized* compressive stress. The inset FFTs indicate the appearance of anisotropy due to the 2D transformation. All scale bars are 500 nm. G) Thickness evolution and z-strain from the as-assembled to the compressed small, medium, and large AuNSs. The represented data indicate an induced vertical out-of-plane z-strain (*ε*_Z_) of 38.12 ± 0.1% (from 18.8 ± 0.4 to 11.6 ± 0.8 nm) for small AuNS, 57.06 ± 0.1% (from 51.6 ± 0.6 to 22.1 ± 2.9 nm) for medium AuNS, and 55.71 ± 0.1% (from 83.6 ± 0.7 to 37.0 ± 1.7 nm) for large AuNS under applied *normalized* compressive stress.

Three different sizes (average diameter) of AuNSs, small (∼18 nm), medium (∼59 nm), and large (∼103 nm), were studied to understand the effect of uniaxial compressive stress on different-sized AuNSs and their resultant solid-state transformation to 2D morphology. These AuNSs were coated with 5k Da PEG (also denoted as “shorter PEG”) capping layer to provide a steric hindrance to maintain interparticle separation and stability in the monolayer assembly, which is estimated as ∼4.5 ± 4.7 nm (small AuNSs), ∼7.9 ± 11.9 nm (medium AuNSs), and ∼7.7 ± 12.7 nm (large AuNSs) (Fig. S1 and Table S2). The as-assembled morphology of the small, medium, and large AuNSs with 5k PEG on the substrate form monolayer hexagonal closed-packed (HCP) assemblies, verified via atomic force microscopy (AFM) and scanning electron microscope (SEM) (Fig. 2A–C; Figs. S2–S4). The AuNSs undergo severe plastic deformation due to applied uniaxial *normalized* compressive stress, with anisotropic expansion on the substrate in the lateral in-plane directions and concomitant thickness reduction in the vertical out-of-plane direction. As a result, the AuNSs deform into various disk-like, oblong, and oval shapes due to compressive stress-induced dislocation nucleation and mobility (Fig. 1B and C). The induced severe plastic deformation phenomenon is evident from the flattened 2D leaf-like morphology, which is attributable to the unique malleability (55) of Au extending down even to the single-digit nanometer scale (Fig. 1D and E).

The deformation of bulk (polycrystalline) Au is dictated by the dislocation mobility, dislocation hindrance, dislocation entanglement, dislocation bowing (Orowan strengthening), dislocation pile-up (Hall–Petch mechanism), etc. (55, 60). At the nanoscale regime in AuNPs, dislocation nucleation, migration toward interfaces, and starvation (less probability of multiplication) dominate the deformation behavior (61). During compression of the AuNSs, elastic deformation is followed by a plastic deformation regime where dislocations initiate at the contact interfaces and propagate through multiple possible slip planes within the particle. Experimentally, different possible compression directions coexist due to the stochastic crystallographic orientations of the individual AuNSs (Schmid factor ranges from 0.37 to 0.49) (Table S3).

The dislocation nucleation and movement are evident from the *postmortem* SEM images where shear bands are prominent (Figs. S5 and S6). Owing to the intrinsic high surface-to-volume ratio of AuNSs, many free facets of AuNSs act as sources and sinks for these dislocations (62, 63). The transformed morphologies of the compressed AuNSs exhibit anisotropic morphologies with significant expansion of the NP lateral sizes and reduction of the interparticle spacing (Fig. 2D–F; Figs. S7–S9). The transformed 2D morphologies from the 0D AuNSs may result from dislocation escape toward the free surfaces of AuNSs upon compression. Eventually, the lateral in-plane expansion causes adjacent AuNSs to narrow the gap of initial interparticle separation and could also introduce sintering via atomic migration at the AuNS boundary edge (64–66). The resultant fast Fourier transform (FFT) pattern of the deformed AuNSs no longer shows distinguishable spots but rather a diffused ring, indicative of a lowering of the NPs' ordering (lower correlation length) and higher distribution of interparticle centroid-to-centroid distances (inset of Fig. 2D–F).

To understand the effect of crystal orientations of the as-assembled AuNSs on the anisotropic transformation process and the resultant 2D morphology, we analyzed the crystallographic evolution of the AuNSs via electron backscattered diffraction (EBSD). While the characterization of the relatively smaller AuNSs was elusive due to limitations in imaging resolution, the crystallographic orientation of the large AuNSs was resolvable both in their as-assembled and compressed 2D morphologies (Fig. S10A–F). The facet orientations of the as-assembled AuNSs appear to be fairly random, with some minor texturing of (212) facets aligned with the (substrate) nominal *z*-direction (Fig. S10C). After compression, the 2D morphology appears to feature predominantly (101) facets aligned with the (substrate) nominal *z*-direction (Fig. S10D). This direct observation of the crystallographic evolution is further evidence of the severe plastic deformation and 2D transformation experienced by the AuNSs. A complex dependency could exist between the transformed 2D morphology, nanostructure, and crystallographic texture stemming from the individual’s initial faceting and crystallographic orientation as-assembled AuNSs and their mutual interactions, which calls for further in-depth exploration beyond the scope of this work.

The morphological evolution and induced z-strain (*ε*_Z_) of different-sized AuNSs are statistically quantified from AFM mapping by comparing the thicknesses of initial as-assembled (*t*_as-assembled_) versus the compressed NPs (*t*_compressed_) (Fig. S11). Here, the vertical out-of-plane z-strain is defined as *ε*_Z_ = (*t*_as-assembled_ − *t*_compressed_)/(*t*_as-assembled_). The effective thicknesses of the as-assembled AuNSs are determined to be 18.8 ± 0.4 nm (small AuNS), 51.6 ± 0.6 nm (medium AuNS), and 83.6 ± 0.7 nm (large AuNS) (Figs. S12–S14 and Table S4). In comparison, the thickness of the compressed anisotropic 2D AuNSs is much thinner than the dimensions of the as-synthesized and the thickness of the as-assembled AuNSs. The thicknesses of the compressed AuNSs are 11.6 ± 0.8 nm (small AuNS), 22.1 ± 2.9 nm (medium AuNS), and 37.0 ± 1.7 nm (large AuNS) (Figs. S15–S17 and Table S4). This reduction in vertical dimension translates to a compression-induced vertical strain (*ε*_Z_) of 38.12 ± 0.1% for small AuNS, 57.06 ± 0.1% for medium AuNS, and 55.71 ± 0.1% for large AuNS. Given the same applied *normalized* stress for all three AuNS sizes, the smaller AuNSs appear to be relatively less compressible.

**Fig. 3.**
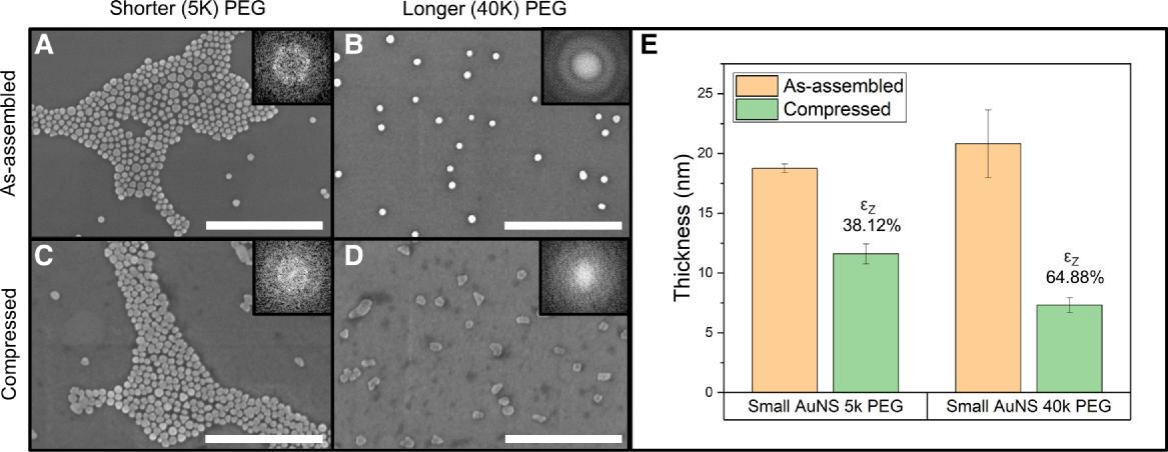
Transformation of small AuNSs into anisotropic 2D morphologies with varied interparticle separation. As-assembled small (20 nm) AuNSs with A) shorter (5k) PEG, B) longer (40k) PEG, and after compression: C) 5k PEG and D) 40k PEG. The C) 5k PEG AuNSs yield lower interparticle distance (∼4.5 ± 4.7 nm) and form 2D morphologies with some interparticle sintering after compression, whereas the D) 40k PEG with larger interparticle distance (∼126.2 ± 65.5 nm) maintains the large interparticle separation and exhibits a higher degree of 2D transformation. The deformation could be influenced by the stochastic crystal facet orientation of the individual AuNSs in isolation. All scale bars 500 nm. E) Thickness evolution and z-strain from the as-assembled to the compressed small AuNSs with 5k PEG and 40k PEG. Under the same applied *normalized* compressive stress, the AuNSs with 40k PEG experience z-strain (*ε*_Z_) of 64.88 ± 0.1% (from 20.8 ± 2.8 to 7.3 ± 0.6 nm), compared with 38.12 ± 0.1% (from 18.8 ± 0.4 to 11.6 ± 0.8 nm) for AuNS with 5k PEG.

In addition to the NP size, the as-assembled interparticle separation and adjacent NP interactions also affect the transformed 2D morphology. More significant interparticle separation can be achieved by (i) reducing the drop-casted AuNS colloidal concentration and/or solution volume and (ii) attaching PEG capping layers of different (longer) lengths. However, just lowering the deposited colloidal concentration and/or solution volume alone is insufficient to effectively modulate the as-assembled interparticle separation, as it yields sparse but agglomerated (closed-packed) patches of AuNSs on the substrate surface. Therefore, the length of the PEG brushes grafted is varied for this study: shorter PEG—molecular weight (MW) 5k Da with a hydrodynamic length of ∼10–18 nm–and longer PEG—MW 40k Da with a hydrodynamic length of ∼34–39 nm (Fig. S18). Along with tuning the colloidal concentration, the varied PEG length modulates the steric hindrance between NPs during the drop-casting and assembly process while providing control over the interparticle separation of the as-assembled AuNSs. Small AuNSs (20 nm) were selected to study the influence of interparticle separation on the 2D morphological transformation of AuNSs (close-packed assembly versus isolated particles). The small AuNSs are suitable for this investigation due to their higher grafting surface density and their relatively larger PEG length to AuNS size ratio, making the effect of interparticle separation more pronounced. After the deposition, the average as-assembled interparticle separation of small AuNS with 40k PEG (∼126.2 ± 65.5 nm) was found to be higher compared with those with 5k PEG (∼4.5 ± 4.7 nm) (Fig. 3A and B; Table S2).

The small AuNSs with 5k PEG mostly remained in close-packed and monolayer arrangement, forming a mesoscopic networked pattern. Any particular AuNSs in this networked structure can have a range of neighboring AuNSs depending on its location: interior AuNSs have six adjacent AuNSs, and edge or corner AuNSs have one to five adjacent AuNSs, which results in varying degrees of lateral resistance under the same applied compression in the *z*-direction (Fig. 3A and C; Figs. S2 and S7). In comparison, the isolated small AuNSs with 40k PEG have larger interparticle separation without lateral resistance from adjacent AuNSs, and the same applied compression induces a higher degree of 2D transformation (Fig. 3B and D; Figs. S19 and S20), transforming the isolated small AuNSs with 40k PEG into oval, disk-like 2D morphologies (Fig. 3D). Statistical analysis of SEM images shows that isolated small AuNSs with 40k PEG exhibit ∼1.9 times larger lateral area expansion due to significantly larger plastic deformation compared with the close-packed small AuNSs with 5k PEG (Fig. S21). In addition, AFM measurements indicate a much larger *ε*_Z_ for isolated small AuNSs with 40k PEG (64.88 ± 0.1%) compared with the closed-packed small AuNSs with 5k PEG (38.12 ± 0.1%) (Fig. 3E; Figs. S22 and S23). This larger deformation of the isolated small AuNSs with 40k PEG in comparison to the closed-packed small AuNSs with 5k PEG is in part attributable to their differing lateral resistance from neighboring AuNSs. Similarly, a higher degree of 2D transformation was observed for isolated large AuNSs with 40k PEG compared with close-packed large AuNSs with 5k PEG (Figs. S24 and S25). AFM measurements corroborate the larger *ε*_Z_ for isolated large AuNSs with 40k PEG (75.54 ± 0.1%) (Figs. S26–S28) compared with the closed-packed large AuNSs with 5k PEG (55.71 ± 0.1%) (Figs. S4 and S9).

Moreover, the 2D transformation of AuNP was also found to be influenced by the magnitude of the applied normalized compressive stress, *σ*_norm_. Close-packed AuNSs with 5k PEG can be progressively compressed to ever-thinner 2D morphologies by applying progressively larger normalized compressive stress (i.e., nanoscale goldbeating) (Fig. S29). In addition, a much larger applied normalized compressive stress (*σ*_norm_ = 6 GPa) induces a higher degree of 2D transformation and z-strain to the isolated AuNSs. Here, a higher *ε*_Z_ for isolated large AuNSs with 40k PEG (80.16 ± 0.1%) was observed under *σ*_norm_ = 6 GPa compared with *ε*_Z_ = 75.54 ± 0.01% under *σ*_norm_ = 3.6 GPa (Figs. S30–S34). It was observed that the relationship between the induced z-strain and the applied compressive stress is nonlinear.

**Fig. 4.**
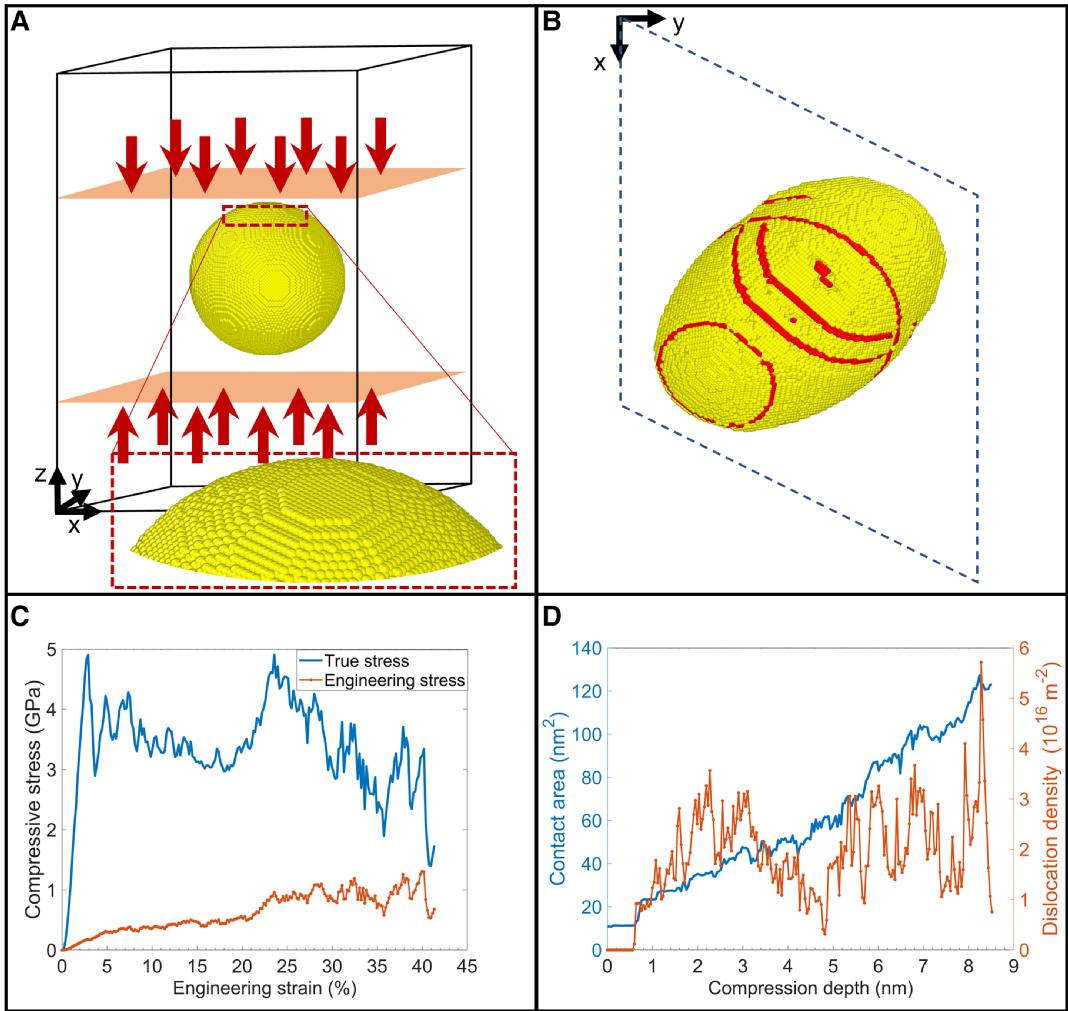
Molecular dynamics simulation of a single AuNS. A) Simulation domain of small AuNS (20 nm in diameter) placed between two planar force fields with a (inset of A) zoomed-in view showing the top few atomic layers of the AuNS. B) Top view of the compressed AuNS at ∼38% strain. Atoms are colored using dislocation analysis (DXA) in OVITO; yellow refers to the FCC structure, and red refers to the HCP structure on the slipping plane. Here, the surface atoms are removed for better visualization. C) Compressive stress variation with respect to engineering strain. The true yield stress and engineering yield stress are calculated to be 4.9 and 0.18 GPa, respectively, at an engineering strain of 2.9%. D) Contact area and dislocation density variation with respect to compression depth at compression velocity of 0.001 nm/ps.

To understand the atomistic deformation mechanisms of AuNSs under solid-state uniaxial compression, classical molecular dynamics (MD) simulations were adopted to simulate the compression of isolated spherical AuNSs (Fig. 4A) at room temperature (300 K). The compression force is applied along the [001] direction of the AuNS with a velocity of 0.001 nm/ps. The compressive stress within the AuNS was evaluated using two metrics (Fig. 4C). The *true* stress is calculated based on the instantaneous contact surface area (shown in Fig. 4D) of the compressing AuNS. The *engineering* stress is calculated based on the initial (maximum, equatorial) cross-sectional area of the AuNS, similar to the empirical applied *normalized* compressive stress defined earlier. When compression starts, the isolated AuNS presents a linear elastic behavior (Fig. 4C) with zero dislocation density and a constant contact surface area (Fig. 4D). When the engineering strain reaches ∼2.9% at a compression depth of 0.6 nm, yielding occurs and the dislocation density starts to increase. The true yield stress is 4.9 GPa, and the corresponding engineering yield stress is 0.18 GPa. The mechanical properties of metal NPs (such as Cu, Au, Al, and Ag) have been reported to be sensitive to particle size, shape, temperature, orientation (55, 67–72), etc. Therefore, the true yield stress (4.9 GPa) and engineering yield stress (0.18 GPa) predicted in our MD simulations reflect reasonable upper and lower limits of the compressive stress for an individual AuNP to initiate the plastic deformation process, which is consistent with the applied *normalized* compressive stress 3.6 GPa we adopted in the empirical solid-state compression process.

As the deformation continues, the top and bottom contact surfaces act as stress concentrators, causing glissile Shockley partial dislocations to form at these contact surfaces on the [111] slip planes as the compression depth reaches 0.6 nm, resulting in a huge increment in dislocation density (Fig. 4D). These glissile dislocations formed on [111] slip planes move inward of the AuNP. As the topmost layer of atoms becomes compressed into the second topmost layer, the Shockley partials symmetrically formed on the {111} planes react with each other, leading to the formation of pyramid hillock structures. Similar pyramid hillock structures have been reported in other face-centered cubic (FCC) spherical monocrystals (Cu and Al) at low temperatures (10 K) through MD simulations (67, 69). The topmost contact area progresses into the second topmost layer of AuNS, causing the sharp decrease of the true stress after yielding. Then the true stress climbs again before new dislocations are nucleated. The critical resolved shear stress (CRSS) is calculated to be 2.3 GPa, which falls in a similar range to previously published results (55, 73, 74). The deformed AuNS appears to have an oblong oval shape at 38% strain, with dislocation nucleation on {111} slip planes (Fig. 4B). Multiple MD simulations were conducted at two different loading rates (0.005 and 0.001 nm/ps). Different deformed shapes were observed due to dynamic evolution of dislocations at 300 K (the selected deformed shapes at 38% strain are listed in Table S5). The variety of deformed AuNS shapes obtained from MD simulations are consistent with the polydispersity of empirically observed anisotropic 2D morphologies (Fig. 3D).

**Fig. 5.**
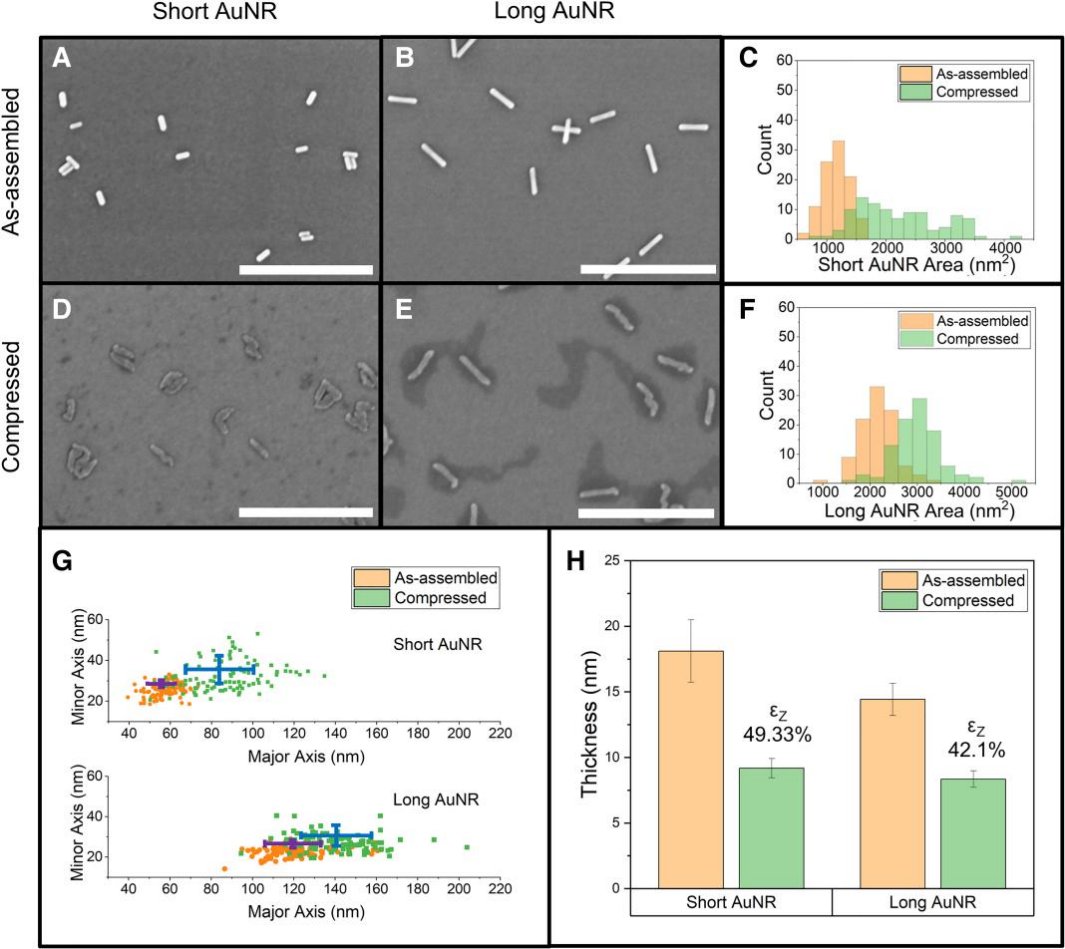
Generalizability of the top-down compression technique to 1D NRs (AuNRs). Monolayer A) as-assembled and D) compressed morphologies of short AuNR (60 × 20 nm, 5k PEG, and AR 3). C) After compression, the lateral area was estimated to be increased by 80% for the short AuNR (from 1,162.4 ± 41.0 to 2,093.2 ± 98.0 nm^2^). Monolayer B) as-assembled and E) compressed morphologies of long AuNR (99 × 14 nm, 5k PEG, and “AR 7”). All scale bars are 500 nm. F) After compression, the lateral area was increased by 37.68% for the long AuNR (from 2,155.0 ± 28.8 to 2,967.0 ± 39.0 nm^2^). G) The elongation in the lateral dimension (estimated using ellipse fitting) of the short and long AuNR is demonstrated in the scatter plots. For the short AuNR, the length (major axis) increased from 57.9 ± 0.7 to 85.8 ± 1.9 nm, and the width (minor axis) increased from 25.2 ± 0.3 to 29.9 ± 0.7 nm after compression. For the long AuNR, the length (major axis) has expanded from 117.6 ± 0.9 to 139.6 ± 1.6 nm, and the width (minor axis) expanded from 23.1 ± 0.2 to 26.8 ± 0.3 nm after compression. H) The induced z-strain of short AuNR due to compression is estimated to be *ε*_Z, short AuNR_ = 49.33 ± 0.1% (from 18.1 ± 2.4 to 9.2 ± 0.7 nm), whereas for long AuNR, the induced z-strain is estimated to be *ε*_Z, long AuNR_ = 42.1 ± 0.1% (from 14.4 ± 1.2 to 8.4 ± 0.6 nm).

Solid-state nanoscale goldbeating enables morphological transformation not just of 0D AuNS precursors but also of other metal NP shapes such as NRs, nanopolyhedrons, nanostars, and nanocages. In particular, AuNRs, as canonical anisotropic 1D metal nanocrystals, have well-established synthesis protocols with precise control over crystallinity, dimensionality (i.e. AR = length/width), and their dependent properties. To demonstrate the generalizability of nanoscale goldbeating, two different-sized AuNRs were transformed into 2D morphologies: short AuNR (60 × 20 nm, “AR 3,” with 5k PEG) and long AuNR (99 × 14 nm, “AR 7,” with 5k PEG). The as-assembled short AuNRs (Fig. 5A; Fig. S35) and long AuNRs (Fig. 5B; Fig. S37) were both subjected to applied uniaxial *normalized* compressive stress. The transformed 2D morphologies of both the short (Fig. 5D; Fig. S36) and long AuNRs (Fig. 5E; Fig. S38) exhibit severe plastic deformation and lateral elongation, transforming from a prismatic morphology into a more flattened, oblong, ellipsoidal morphology with nonstraight edges along the axial direction and in some cases bent rod ends. Regardless of the precursor AuNR size, significant deformation is evident in the transformed 2D morphology, along with the presence of shear bands (Fig. S6).

Statistical metrology analysis of individual AuNRs was performed to gain further insight into the morphological transformation. The projected lateral areas for the as-assembled and compressed AuNRs were quantified from SEM images (Fig. S39A and B). Metrological analysis of the lateral elongation along the AuNR axial length and radial width quantifies the in-plane (i.e. substrate plane) anisotropic deformation. However, characterizing the nominal length and width will generate erroneous conclusions due to the nonuniform boundary edges of the compressed AuNR morphology. Therefore, the closest approximation of the as-assembled and compressed AuNRs shape is performed by ellipse fitting and extracting the major (length) and minor (width) axis information (Fig. S39C and D). Each AuNR was analyzed via ellipse fitting by equating their enclosed area and equating their second-order central moment of area.

The aforementioned statistical metrology analysis of the average lateral area indicated that the short AuNRs were expanded by 80% (from 1,162.4 ± 41.0 to 2,093.2 ± 98.0 nm^2^) as a result of compression (Fig. 5C; Fig. S40), whereas the long AuNRs experienced an average lateral area increase of 37.68% (from 2,155.0 ± 28.7 to 2,967.0 ± 38.9 nm^2^) (Fig. 5F; Fig. S42). Due to severe plastic deformation, the short AuNR length (major axis) expanded from 57.9 ± 0.7 to 85.8 ± 1.9 nm, and the width (minor axis) expanded from 25.2 ± 0.3 to 29.9 ± 0.7 nm (Fig. 5G; Fig. S41). Similarly, after compression, the long AuNR length (major axis) increased from 117.6 ± 0.9 to 139.6 ± 1.6 nm, and the width (minor axis) expanded from 23.1 ± 0.2 to 26.8 ± 0.3 nm (Fig. 5G; Fig. S43).

The vertical z-strain for transformed AuNRs was statistically derived from AFM height maps. The thicknesses of the as-assembled and compressed short AuNRs indicate an induced z-strain *ε*_Z, short AuNR_ of 49.33 ± 0.1% (from ∼18.1 ± 2.4 to ∼9.2 ± 0.7 nm) (Fig. 5H; Figs. S44 and S45), whereas the long AuNRs exhibited a slightly smaller *ε*_Z, long AuNR_ of 42.1 ± 0.1% (from 14.4 ± 1.2 to 8.4 ± 0.6 nm) (Fig. 5H; Figs. S46 and S47). Considering both the thickness reduction (z-strain) and the average lateral area expansion, the more slender, long AuNRs appear to be relatively less compressible compared with the girthier, short AuNRs under the same applied *normalized* compressive stress, suggested by earlier studies (75). Previous computational and experimental efforts on similar Au nanocrystals (i.e. nanowires) have primarily focused on axial tensile or nanoscale bending tests (57, 76–79). These observed dependencies of mechanical properties on the size of metallic nanowires generally exhibited trends of smaller nanowires having higher strength. Our results here provide a different perspective to the existing literature by studying the deformation of NRs in the radial direction, which warrants further investigations to gain a better understanding of the mechanisms underlying metallic nanocrystal deformation. Overall, it is evident that our solid-state 2D transformation technique is extendable to study the deformation and transformation of other nanocrystals beyond 0D AuNSs and 1D AuNRs.

## Conclusion

The ancient craft of goldbeating is extended by several orders of magnitude down to the single-digit nanometer scale via solid-state, uniaxial compression of 0D AuNSs and 1D AuNRs, transforming them into anisotropic 2D Au leaf morphologies. The resultant 2D morphologies are found to be influenced by the precursor Au nanocrystal morphology, dimensions (diameter, AR), and interparticle interactions. In addition, the ability to induce additional NP shape anisotropy enables control of the leaf-like 2D Au's shape, lateral size, thickness, and crystallinity. We postulate that this nanoscale goldbeating process is potentially compatible with nanoimprinting or nanoembossing techniques to induce various hierarchical morphologies. Such versatility and generalizability of this solid-state compression methodology could open new pathways to investigate interesting morphological transformations and strain-induced emergent phenomena across a broad palette of nanocrystals.

## Materials and methods

### Preparation of 0D AuNS and 1D AuNR

Small AuNSs (∼18 nm) were prepared via the Turkevich method (80) (Fig. S48). One hundred milliliters of nanopure H_2_O and 250 µL of 100 mM HAuCl_4_ were added to a 125-mL flask. The solution was stirred at 600 rpm and brought to a rapid boil before adding 7 mL of 1% sodium citrate (m/v). The solution was then stirred for 30 min at just below boiling. An additional 2.5 mL of 1% sodium citrate (m/v) was added, and the reaction was stirred while heating for 10 min. The heat was then turned off, and the particles were cooled to near-room temperature with continued stirring (60 min). The solution was centrifuged at 9,000 RCF for 20 min, the supernatant was removed, and the pellet was resuspended in 10 mL nanopure H_2_O.

Medium (∼59 nm) and large (∼103 nm) AuNSs were prepared via the seed-mediated synthesis by Chan et al. (81) (Fig. S48). As outlined above, small Au seeds (14 nm) were prepared by the Turkevich method, with 60 mL nanopure H_2_O, 0.1524 mL of 100 mM HAuCl_4_, and 1.8 mL of 1% sodium citrate (m/v). The reaction was stirred for 10 min just below boiling, and the second sodium citrate addition was omitted. After cooling the particles to near-room temperature, the solution was centrifuged at 11,000 RCF for 20 min, the supernatant was removed, and the pellet was resuspended in 10 mL nanopure H_2_O. For the growth of the 59 and 103 nm AuNSs, 480 mL nanopure H_2_O and 1.27 mL of 100 mM HAuCl_4_ were added to two 500-mL flasks. While stirring at 800 rpm, Au seed solution was added to each flask to reach a final seed concentration of 0.0353 nM (for 59 nm AuNS) and 0.0127 nM (for 103 nm AuNS). 1.1 mL 1% sodium citrate (m/v) was added to each flask. The reactions were stirred 5 s, followed by rapidly adding 5 mL of 30 mM hydroquinone. The reactions were stirred at 800 rpm at 25°C for 60 min. Particles were then centrifuged at 4,000 RCF (59 nm AuNS) and 2,000 RCF (103 nm AuNS) for 15 min, supernatants were removed, and pellets were resuspended in 10 mL nanopure H_2_O.

Short AuNRs (47 × 16 nm, AR 3) were prepared via our previously reported method (82). Single-crystal cetyltrimethylammonium bromide (CTAB)-capped seeds were prepared by rapid injection of ice-cold 10 mM aqueous sodium borohydride into a rapidly stirring solution of 10 mL aqueous 100 mM CTAB and 0.25 mM HAuCl_4_. The reaction was stirred rapidly for 10 min and then left still for 1 h. For NR growth, 475 mL 100 mM aqueous CTAB, 1.25 mL 100 mM aqueous AgNO_3_, and 25 mL 10 mM HAuCl_4_ were added to a 1-L flask. Next, 2.75 mL 0.1 M ascorbic acid was added, which caused the solution to turn colorless. Finally, 600 μL of the Au seed solution was added, and after thorough mixing, the reaction was kept still at 27°C overnight. The solution was centrifuged at 4,500 RCF for 15 min, and the particles were washed twice in 1 mM aqueous CTAB.

Long AuNRs (99 × 14 nm, AR 7) were prepared via the synthesis method of Vigderman and Zubarev et al. (81). Single-crystal CTAB-capped seeds were prepared by rapid injection of 460 µL ice-cold 10 mM sodium borohydride in 10 mM aqueous NaOH into a rapidly stirring solution of 10 mL aqueous 100 mM CTAB and 0.50 mM HAuCl_4_. The reaction was stirred rapidly for 10 min and then left still for 1 h. For NR growth, 475 mL 100 mM aqueous CTAB, 1.5 mL 100 mM aqueous AgNO_3_, and 25 mL 10 mM HAuCl_4_ were added to a 1-L flask. Next, 25 mL of 100 mM hydroquinone was added, which caused the solution to turn colorless. Finally, 8 mL of the Au seed solution was added, and after thorough mixing, the reaction was kept still at 27°C overnight. The solution was centrifuged at 4,500 RCF for 15 min, and the particles were washed twice in 1 mM aqueous CTAB.

All particles were functionalized with mPEG-SH to reduce NP aggregation upon deposition. Particles were diluted to 1 nM in 0.2 mM mPEG-SH MW 5k (shorter) PEG or 0.1 mM mPEG-SH MW 40k (longer) PEG. Excess capping ligands were removed from the particles by washing them six times with nanopure water, followed by dispersion in nanopure water to the target particle concentration.

### AuNS and AuNR assembly on silicon substrate

Polished silicon wafers (prime grade, UniversityWafer, Inc.) were used for assembling AuNS and AuNR. The silicon wafers were diced into 2 mm × 2 mm square substrates and cleaned using deionized (DI) water, acetone, and isopropyl alcohol (IPA) via ultrasonication, followed by piranha cleaning (3:1 sulfuric acid and 30% hydrogen peroxide) and DI water rinsing. Silicon substrates were further undergone through O_2_ plasma to increase hydrophilicity. The concentration and volume of the colloidal AuNP solutions were tailored to the desired as-assembled area coverage and then drop-casted onto the silicon wafers and left to dry slowly to ensure monolayer assembly of the AuNPs. The as-assembled AuNP/Si was characterized via SEM and AFM to statistically quantify the area of coverage and confirm monolayer closed-packed assembly.

### Compression of AuNP

Uniaxial compression was applied to the AuNPs via a custom-built hydraulic press setup ensuring solid-state, hard-contact compression. This study uses this custom technique to ensure displacive motion-mediated structural transformation, in contrast to in situ deformation within transmission electron microscopy (TEM) setups, which is often dominated by diffusion-mediated structural reconstruction due to electron beam–induced sample heating and atomic diffusion (43). Silicon wafers prepared in the same manner as the AuNP assembly substrate were diced into 1 mm × 1 mm squares as top compression surfaces. To prevent the top compression surface from picking up the AuNPs (i.e. to keep the AuNPs on the silicon substrate), a self-assembled monolayer (SAM) was deposited onto the top compression surfaces by exposing them to trichloro(1H,1H,2H,2H-perfluorooctyl)silane vapor in a vacuum desiccator (Fig. S49) (83). The compression force was measured using a flat membrane box load cell sensor. The duration of the compression transformation process is ∼10 s. Our solid-state uniaxial compression was performed at a loading rate of 0.58–1.86 nm/s (i.e. strain rate of 0.03–0.19/s), with the compressive force held stable for ∼10 s before unloading.

### Estimation of the *nominal* compression stress and *normalized* compression stress

The custom-made solid-state compression setup consists of a top compression silicon wafer of 1 mm × 1 mm and a bottom substrate silicon wafer of 2 mm × 2 mm. Colloidal AuNP solution is deposited on the bottom substrate silicon wafer and then compressed with the top compression silicon wafer. The applied *nominal* stress (*σ*_nom_) is calculated by considering the nominal force (*F*_nom_) applied over the total nominal contact area (*A*_nom_) between the top compression silicon wafer and the bottom substrate silicon wafer (*A*_nom_ = ∼1 mm^2^). The applied *normalized* stress (*σ*_norm_) is calculated by considering the *nominal* force (*F*_nom_) acting on the area covered by the as-assembled AuNPs on the substrate (Figs. S50 and S51). Therefore, *σ*_norm_ = (*σ*_nom_/area coverage of AuNP) = (*F*_nom_)/(*A*_nom_ × area coverage of AuNP). Evenly spaced, 13 positions (with polar symmetry) are statistically sampled across each of the as-assembled AuNP/Si wafers to determine the area coverage of AuNP (Table S6). The as-assembled samples with uniform coverage (with a statistical coefficient of variation [COV] of less than 30%) are used for compression to ensure uniform compressive stress distribution.

### Molecular dynamics simulation

The LAMMPS package (84) was adopted to perform the classical molecular dynamics simulations. Spherical AuNSs of diameter 20 nm were carved out from a perfect FCC Au crystal structure (Fig. 4A). The lattice constant is 0.408 nm. The simulation box size is 40.4 nm × 32.23 nm × 59.99 nm with a *xy* tilt of 18.6 nm. The vacuum space within the simulation box keeps AuNS isolated from interactions and allows AuNS to deform freely under compression. The embedded atom method (EAM) potential (85) was adopted to describe the pairwise interactions of Au atoms. The AuNS was first equilibrated at 300 K under an NVT ensemble for structure relaxation (86). The center of mass and momentum of AuNS is confined to eliminate the rigid body translation and rigid body rotation motions. After relaxation at 300 K, the AuNS was placed between two virtual planar compression surfaces. The planar surfaces are presented by two repulsive force fields moving toward the center of AuNS with a constant velocity of 0.001 nm/ps. The uniaxial compression is along the *z*-direction aligned with the [001] crystallographic orientation of the Au crystal. The loading speed is faster than the experimental setup but appears to have a minimum impact on the deformation mechanism of AuNS during the compression. The stiffness of the force field was defined as 1,000 eV/Å^−3^, equivalent to rigid body compression. The time step size is 1 fs. During compression, the force applied on AuNS was calculated by the reaction force exerted on the planar compression surface. The compressed contact surface area was calculated using Delaunay triangulation (87). The dislocation density was evaluated through the visualization software OVITO (88).

### EBSD

EBSD mapping was performed using an FEI Helios G4 PFIB equipped with an EDAX Velocity EBSD camera, a 10-kV beam voltage, a beam current of 6.4 nA, and a nominal working distance of approximately 6 mm. All EBSD data sets (maps) were collected using EDAX TEAM software with a 6-nm step size, the “Medium-Large” Hough mask setting, 8 × 8 EBSD pattern binning, and the “Enhanced” EBSD pattern digital processing setting. The EBSD pattern at each point in the map was simultaneously recorded during the collection of the map data set. Subsequently, EDAX OIM 8 software was used to generate the inverse pole figure maps and pole figures for each data set. Finally, neighbor pattern averaging and reindexing was performed to improve indexing accuracy using the acquired EBSD patterns for each map data set.

## Supplementary Material

pgad267_Supplementary_Data

## Data Availability

All data that support the findings of this study are available in the manuscript and the Supplementary material.
