# Supplementary material for: Nanoscale goldbeating: Solid-state transformation of 0D and 1D gold nanoparticles to anisotropic 2D morphologies
Source: PNAS Nexus. 2023 Aug 18;2(8):pgad267. doi: 10.1093/pnasnexus/pgad267 (PMC10446819; doi:10.1093/pnasnexus/pgad267)
Supplement: pgad267_Supplementary_Data [file pgad267_supplementary_data.docx]

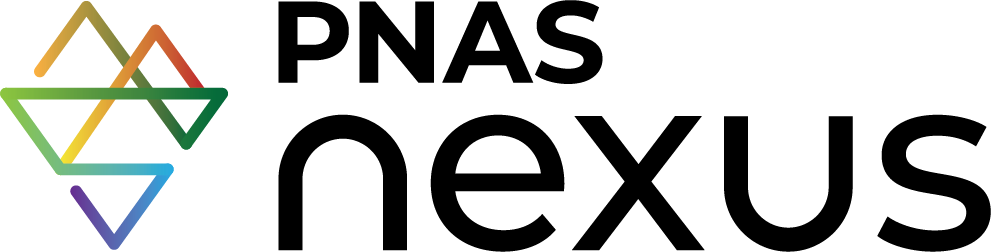


**Supporting Information for**

Nanoscale Goldbeating: Solid-State Transformation of 0D and 1D Gold Nanoparticles to Anisotropic 2D Morphologies

Md Rubayat-E Tanjil^a^*, Tanuj Gupta^b^*, Matthew T. Gole^c^*, Keegan P. Suero^a^, Zhewen Yin^a^, Donald J. McCleeary^a^, Ossie R. T. Douglas^a^, Maegen M. Kincanon^c^, Nicholas G. Rudawski^d^, Alissa B. Anderson^e^, Catherine J. Murphy^c^**, Huijuan Zhao^b**^, Michael Cai Wang^a, f, g^**.

*These authors contributed equally to this research work.

**To whom correspondence may be addressed.

**Email:**  Michael Cai Wang ([mcwang@usf.edu](mailto:mcwang@usf.edu)), Huijuan Zhao ([hzhao2@clemson.edu](mailto:hzhao2@clemson.edu)), Catherine J. Murphy ([murphycj@illinois.edu](mailto:murphycj@illinois.edu)).

**This PDF file includes:**

Supporting text

Figures S1 to S51

Tables S1 to S6

SI References

**Supporting Information Text and Figures**

**Interparticle separation (IPS) calculation**

The interparticle separation (IPS) of the as-assembled AuNSs is calculated by analyzing SEM images. The SEM images are obtained of AuNSs at 100kx and processed using ImageJ software to perform binary conversions. A Fast Fourier transformation (FFT) pattern of the binary images is obtained. Subsequently, six distinguished FFT spots were used to estimate the centroid to centroid distance for the as-assembled AuNSs (Fig. S1). The average centroid-to-centroid distance (d_cc_) was calculated statistically by averaging data from SEM images from thirteen spots across the “central region”. We denote the particle diameter as d_p_, so IPS = d_cc_ – d_p_. The average particle diameter is estimated from the nominal particle projection area from SEM images for each sized AuNS. The IPS data for different-sized AuNS with shorter (5k) and longer (40k) PEG are all tabulated in Table S2.

The as-assembled small AuNSs with 40k PEG are spaced far from each other. After performing the same image analysis, the generated FFT pattern does not show any distinguishable pattern to quantify the centroid-to-centroid distances. Therefore, for as-assembled small AuNS with 40k PEG, the IPS is calculated from SEM images (Fig. S19) by manually determining the centroid-to-centroid distance between 100 pairs of AuNSs using ImageJ.


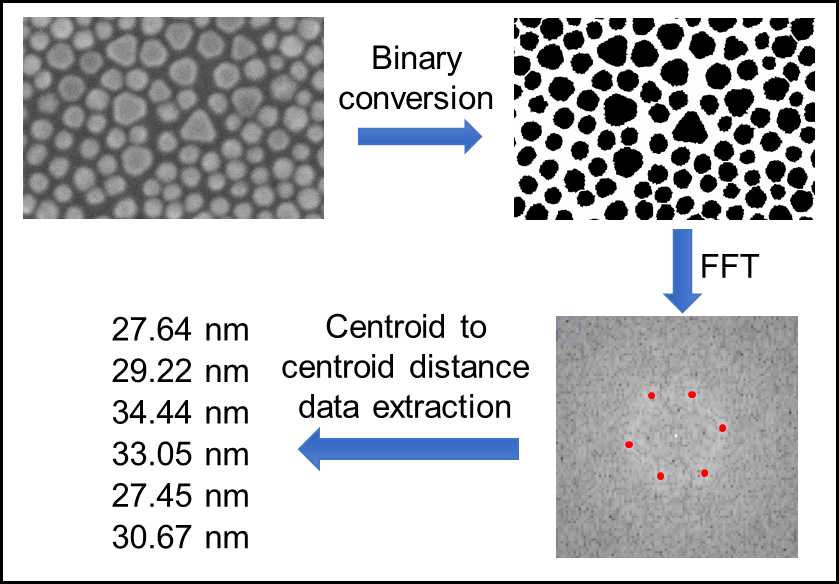


**Fig. S1.** Example of centroid-to-centroid distance calculation for small AuNS with 5k PEG, position 1. SEM image is converted to binary; subsequently, centroid to centroid distance was extracted from the FFT.

**The morphology of as-assembled AuNS 5k PEG**

All scanning electron microscope (SEM) images are captured with a Hitachi model SU70 SEM.

**
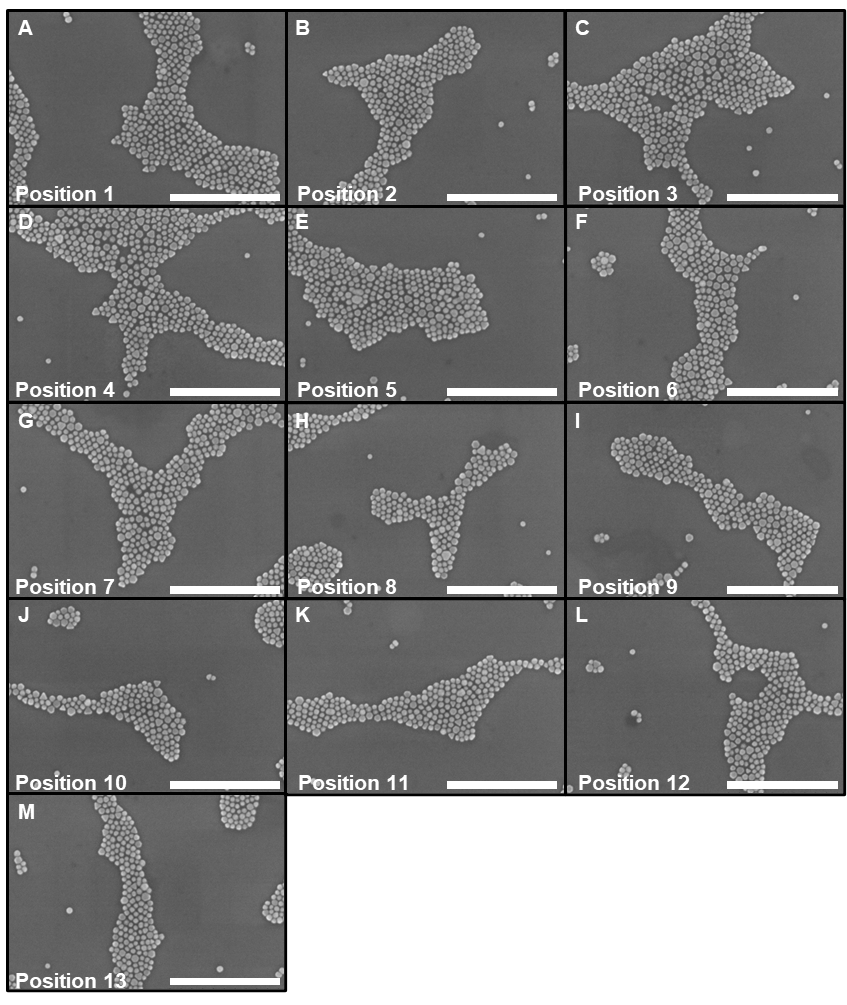
**

**Fig. S2.** A) - M) SEM images of as-assembled small AuNS with 5k PEG on silicon substrate from position 1 to position 13. All scale bars are 500 nm.

**
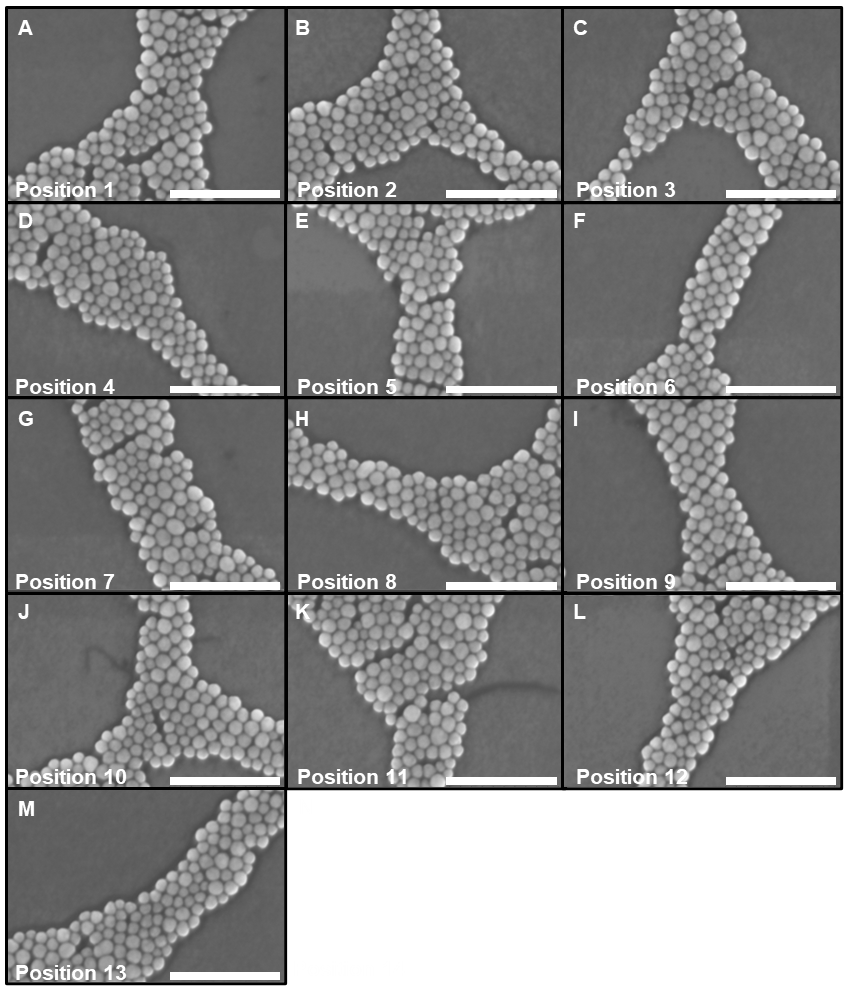
**

**Fig. S3.** A) - M) SEM images of as-assembled medium AuNS with 5k PEG on silicon substrate from position 1 to position 13. All scale bars are 500 nm.

**
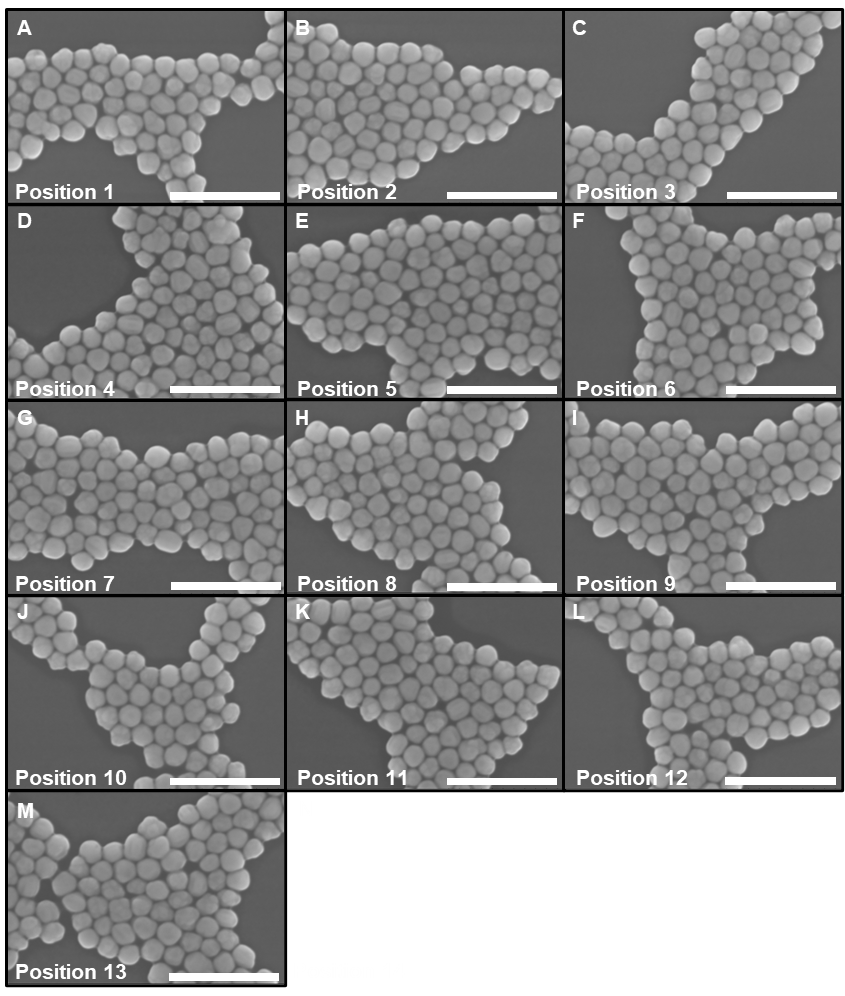
**

**Fig. S4.** A) - M) SEM images of as-assembled large AuNS with 5k PEG on silicon substrate from position 1 to position 13. All scale bars are 500 nm.

**The deformation-induced shear bands in AuNSs and AuNRs**

The compression-induced plastic deformation causes dislocation nucleation and movement in the AuNPs (AuNSs and AuNRs). The solid-state deformation process is performed in ambient conditions and is likely dominated by displacement-mediated structural transformation over diffusion-mediated structural transformation. In the *post-mortem* SEM images, we observed prominent shear bands in the compressed AuNSs (Fig S5) and AuNRs (Fig S6), suggesting the dislocation nucleation and movement and displacement-mediated nature of the transformation.


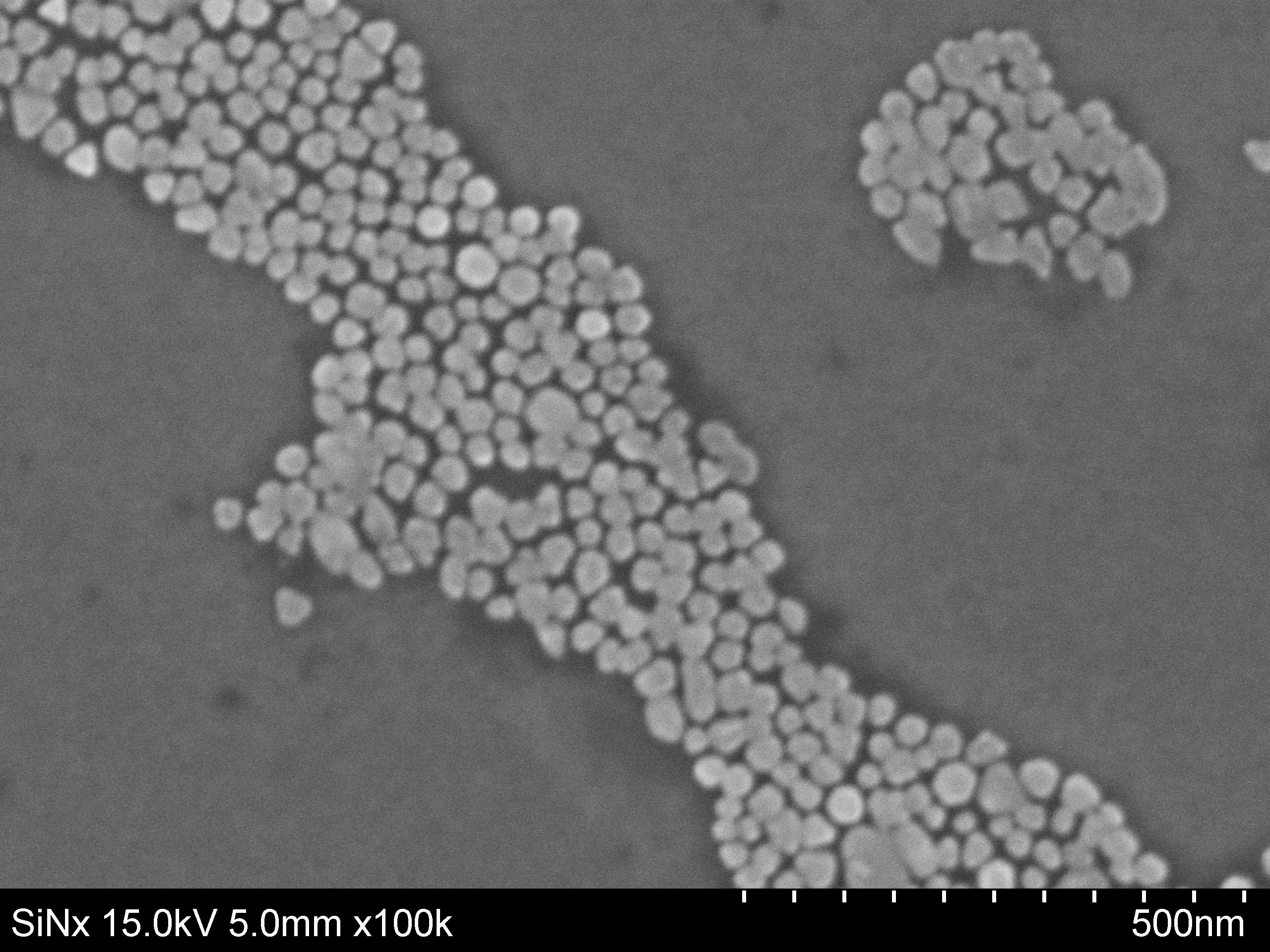


Small AuNS with 5k PEG

F


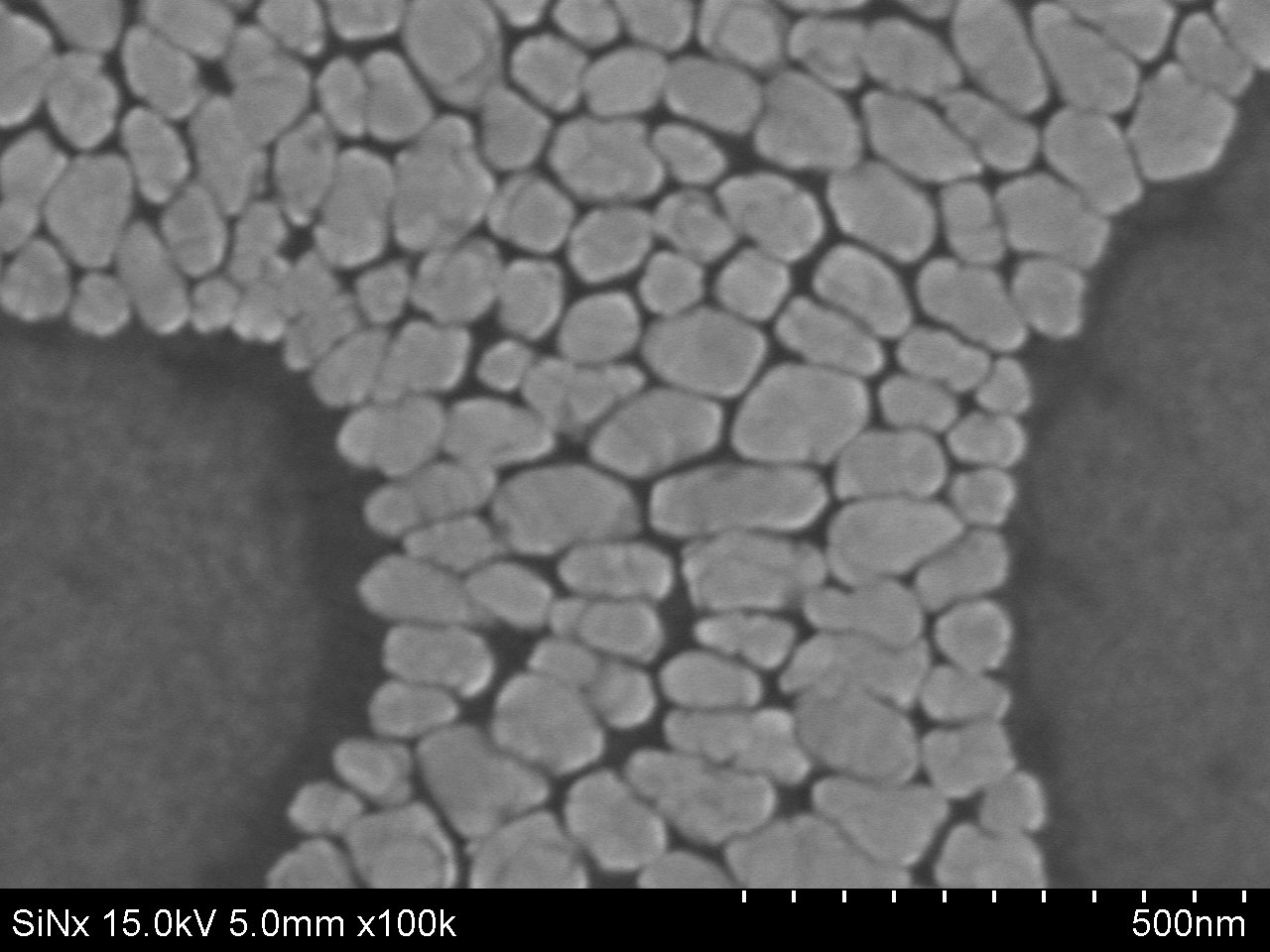


B

A

Medium AuNS with 5k PEG


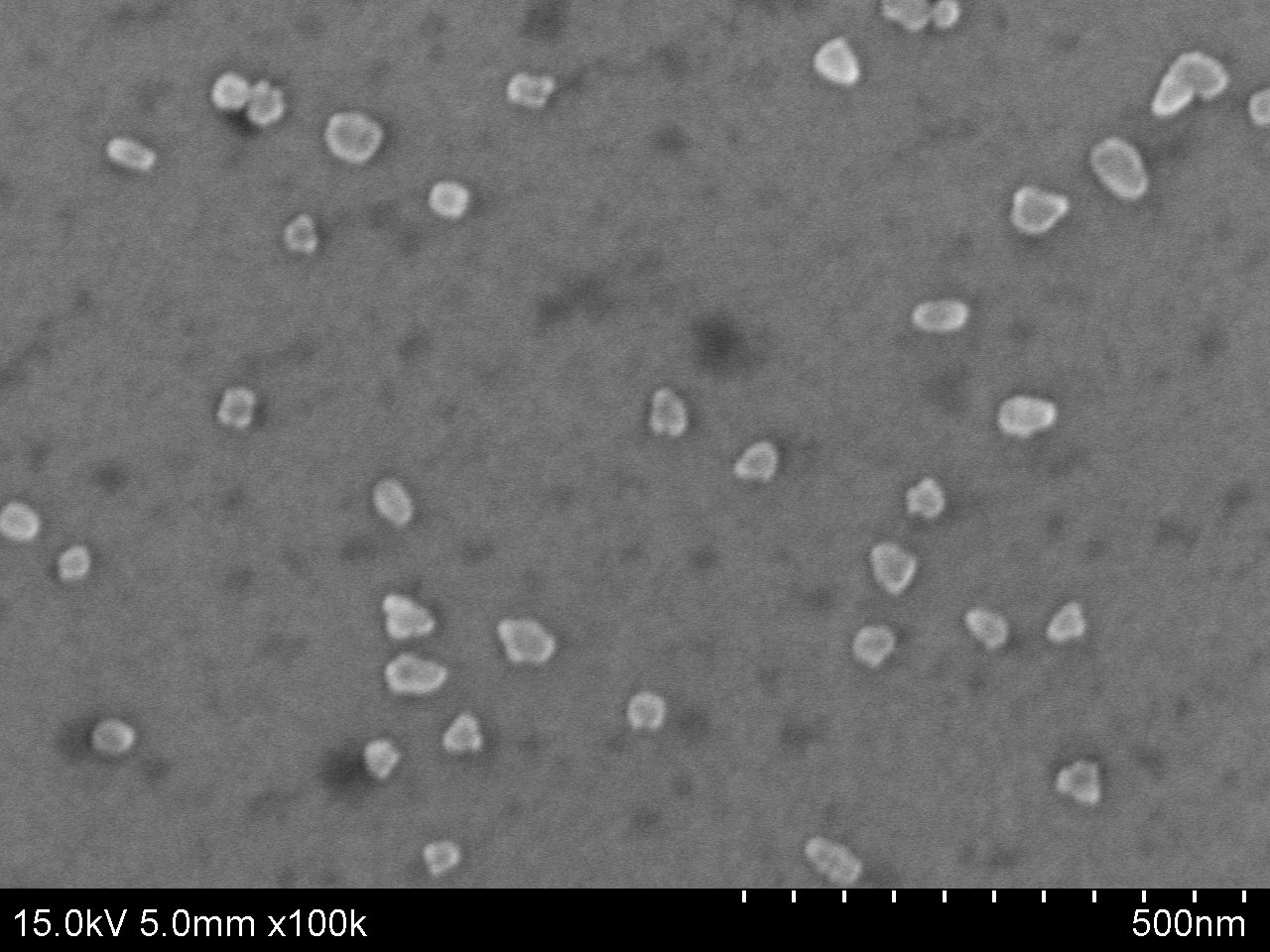


D

Small AuNS with 40k PEG


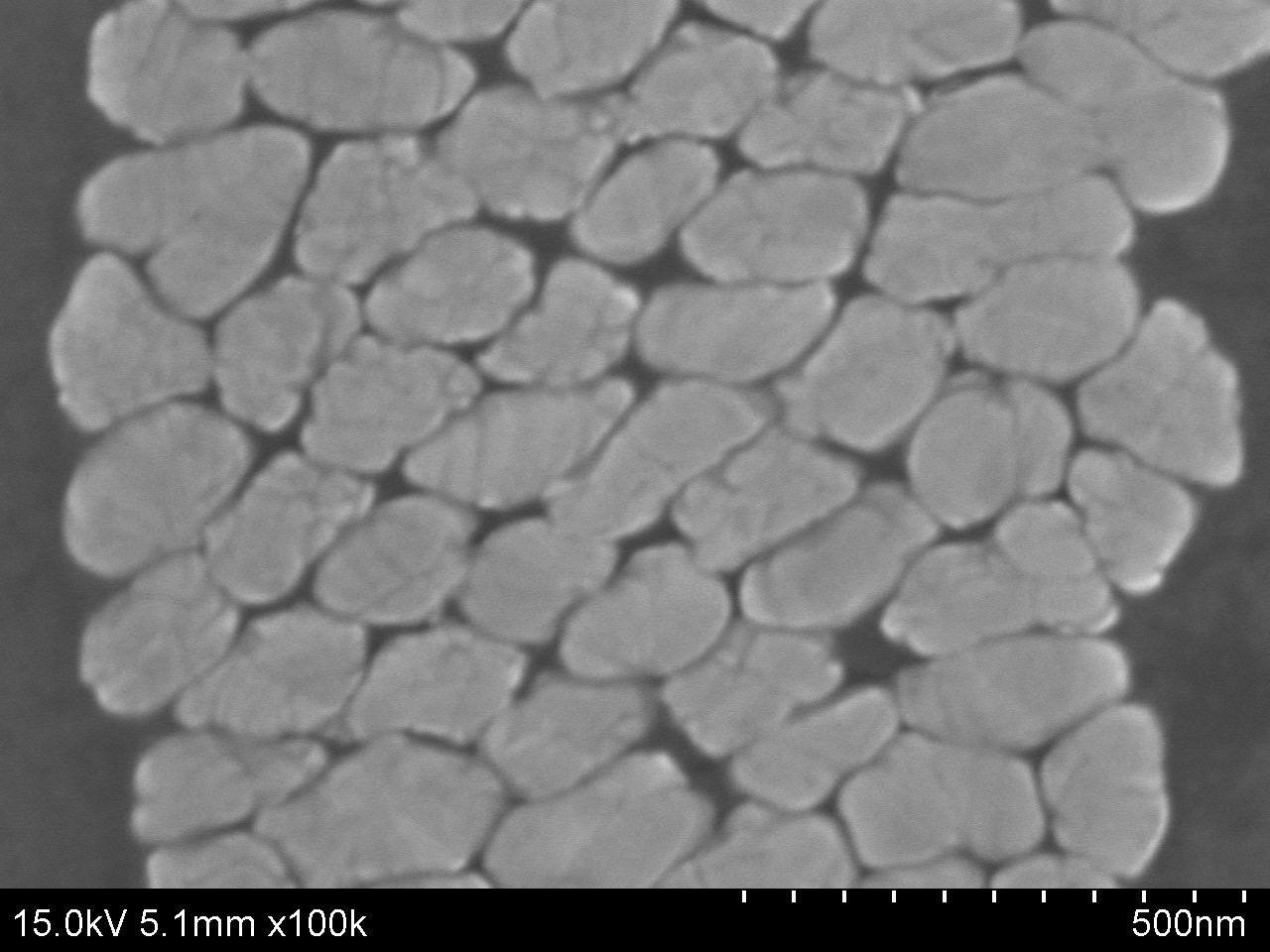


C

Large AuNS with 5k PEG

**Fig. S5.** The AuNSs undergo severe plastic deformation during uniaxial solid-state compression. The shear bands are prominent (pink dotted line) in the post-mortem SEM images for A) small AuNSs with 5k PEG, B) medium AuNSs with 5k PEG, C) large AuNSs with 5k PEG, and D) small AuNSs with 40k PEG. All scale bars are 100 nm.


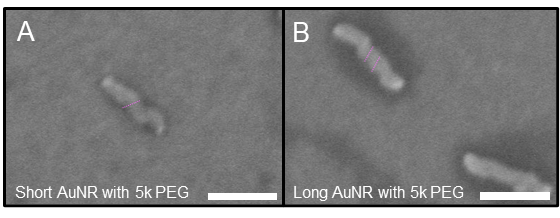


**Fig. S6.** The AuNRs undergo severe plastic deformation during uniaxial solid-state compression. The shear bands are prominent (pink dotted line) in the post-mortem SEM images for A) short AuNR 5k PEG and B) long AuNR with 5k PEG. All scale bars are 100 nm.

**The morphology of compressed AuNS 5k PEG**


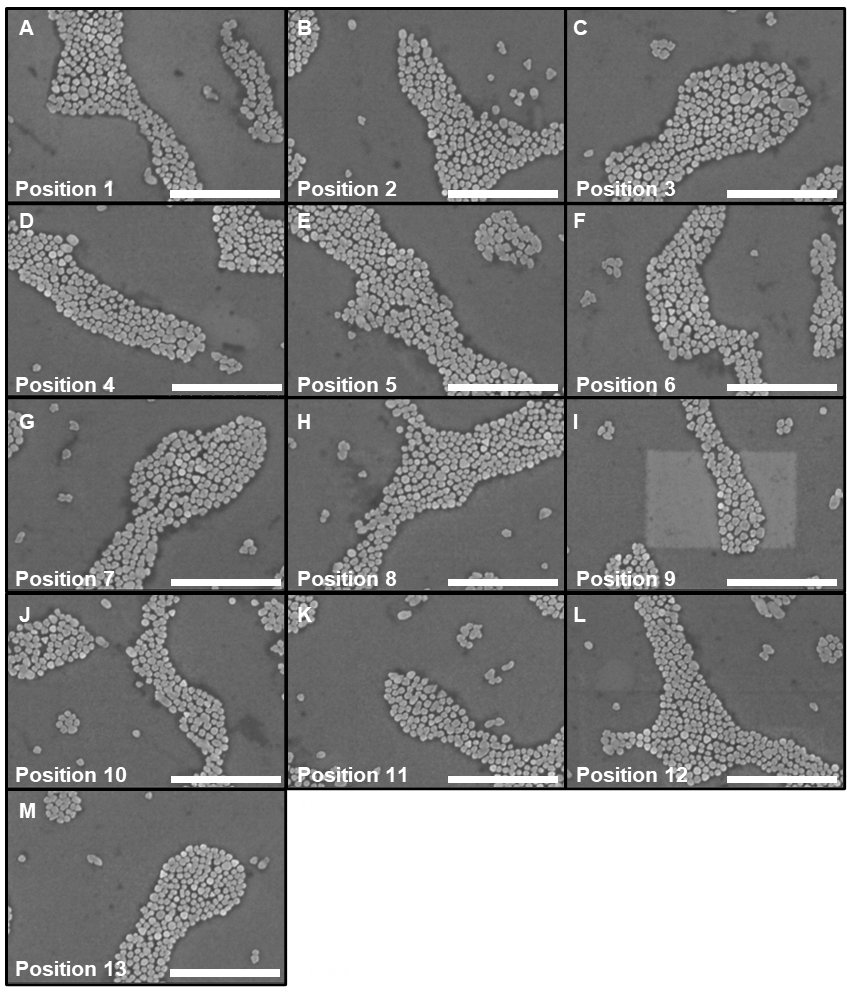


**Fig. S7**. A) - M) SEM images of compressed small AuNS with 5k PEG on silicon substrate from position 1 to position 13. All scale bars are 500 nm.


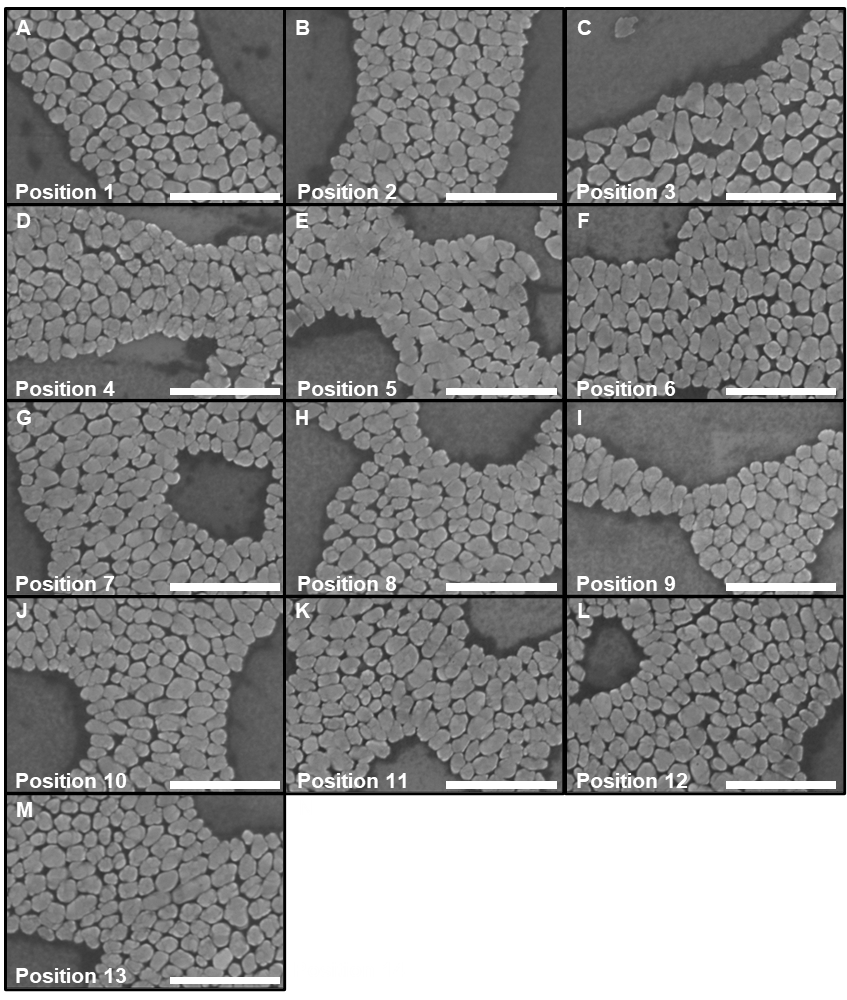


**Fig. S8**. A) - M) SEM images of compressed medium AuNS with 5k PEG on silicon substrate from position 1 to position 13. All scale bars are 500 nm.


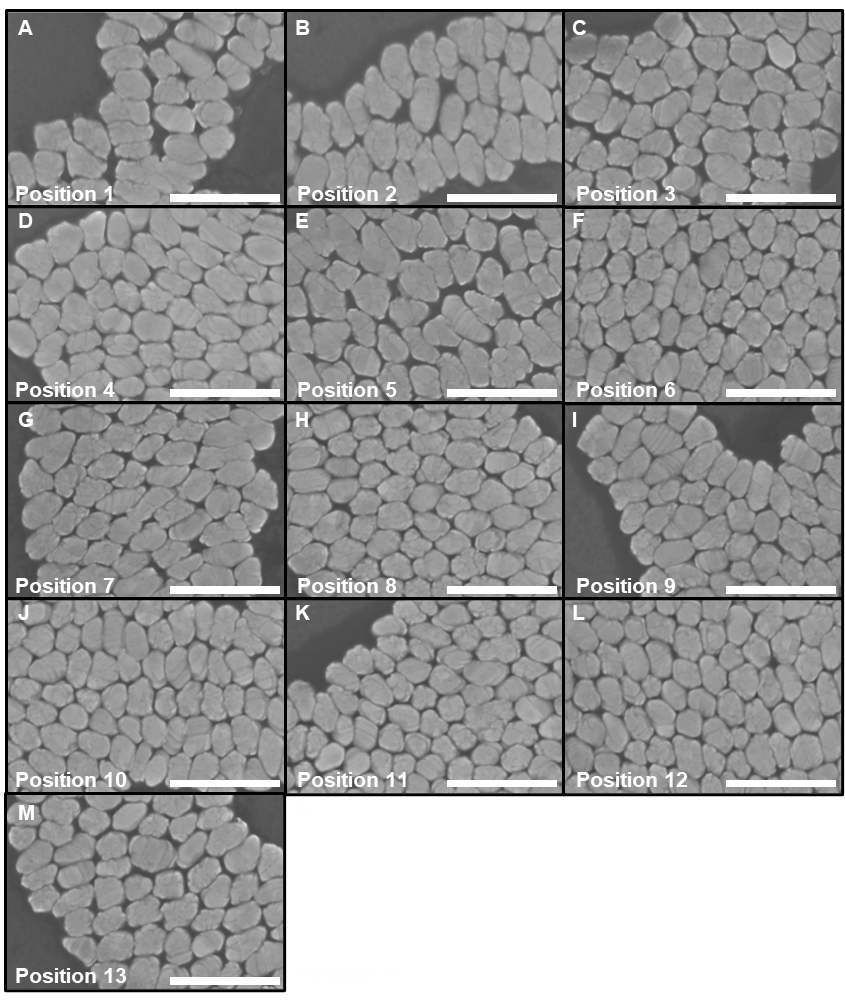


**Fig. S9**. A) - M) SEM images of compressed large AuNS with 5k PEG on silicon substrate from position 1 to position 13. All scale bars are 500 nm.

**Crystallographic evolution of AuNS**

The crystallographic orientation of the as-assembled and compressed AuNS was evaluated using electron backscatter diffraction (EBSD) (FEI Helios G4 PFIB) (Fig. S10).

**
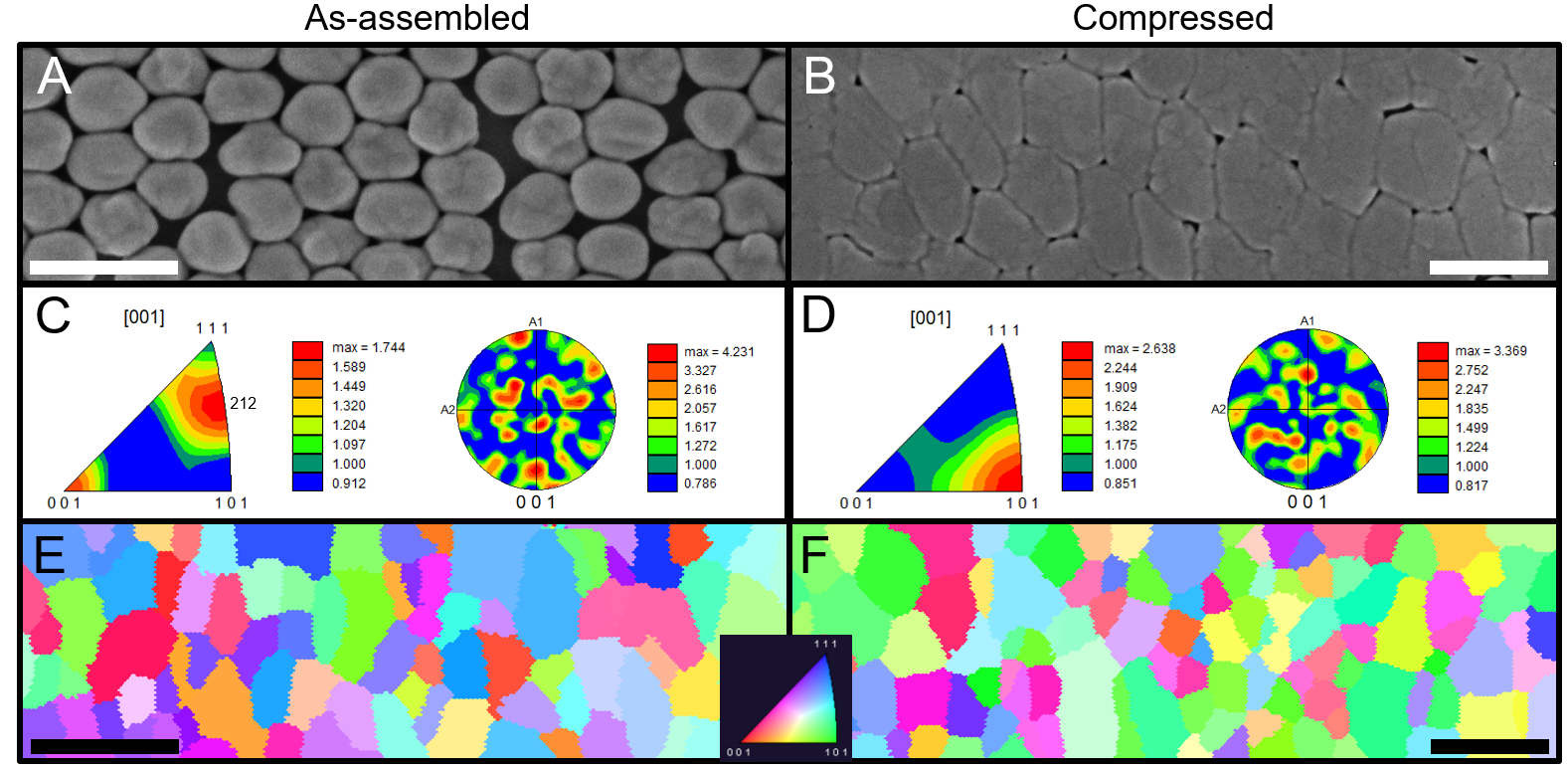
**

**Fig. S10.** Crystallographic evolution of AuNSs characterized via EBSD. The SEM images of A) as-assembled and B) compressed large AuNSs; The corresponding inverse pole figures (left) and {001} pole figures (right) indicate the crystallographic orientation of the C) as-assembled and D) compressed large AuNSs. The corresponding inverse pole figure orientation maps show the spatial distribution of the preferential crystallographic orientations E) before and F) after compression of the AuNSs. All scale bars are 200 nm.

**Characterization of AuNS 5k PEG Morphology and Its 2D Transformation**

The morphology of AuNSs (especially in the z-direction) is characterized via Atomic Force Microscopy (AFM) mapping. For statistical analysis of the thickness evolution and understanding of the uniformity of the compression, ten AFM datasets across the sample are acquired for each as-assembled and compressed sample (Fig. S11*A* and Table S4). All AFM measurements are acquired via a Veeco Dimension 3000.

Figs. S12-17, showing the AFM maps for as-assembled and compressed small, medium, and large AuNSs. Each AFM map is statistically analyzed to estimate the thickness of the as-assembled AuNSs or the compressed 2D morphology. The AFM images are post-processed, with height histogram information extracted, and fitted with Gaussian distributions.

$y=y_{0}+\left( \frac{A}{W\times\sqrt{\frac{\pi}{2}}} \right)\times e^{(-2\times\left( \frac{X-Xc}{w} \right)^{2})}$ (1)

The histogram is fitted with two independent Gaussians, corresponding to the pixel sampling of the bare silicon substrate and those of the (as-assembled or compressed) AuNSs. The thickness is calculated from the difference between two fitted means, which corresponds to the difference between the bare substrate and the average height of the as-assembled or compressed AuNSs (Figs. S11*B* and S11*C*).


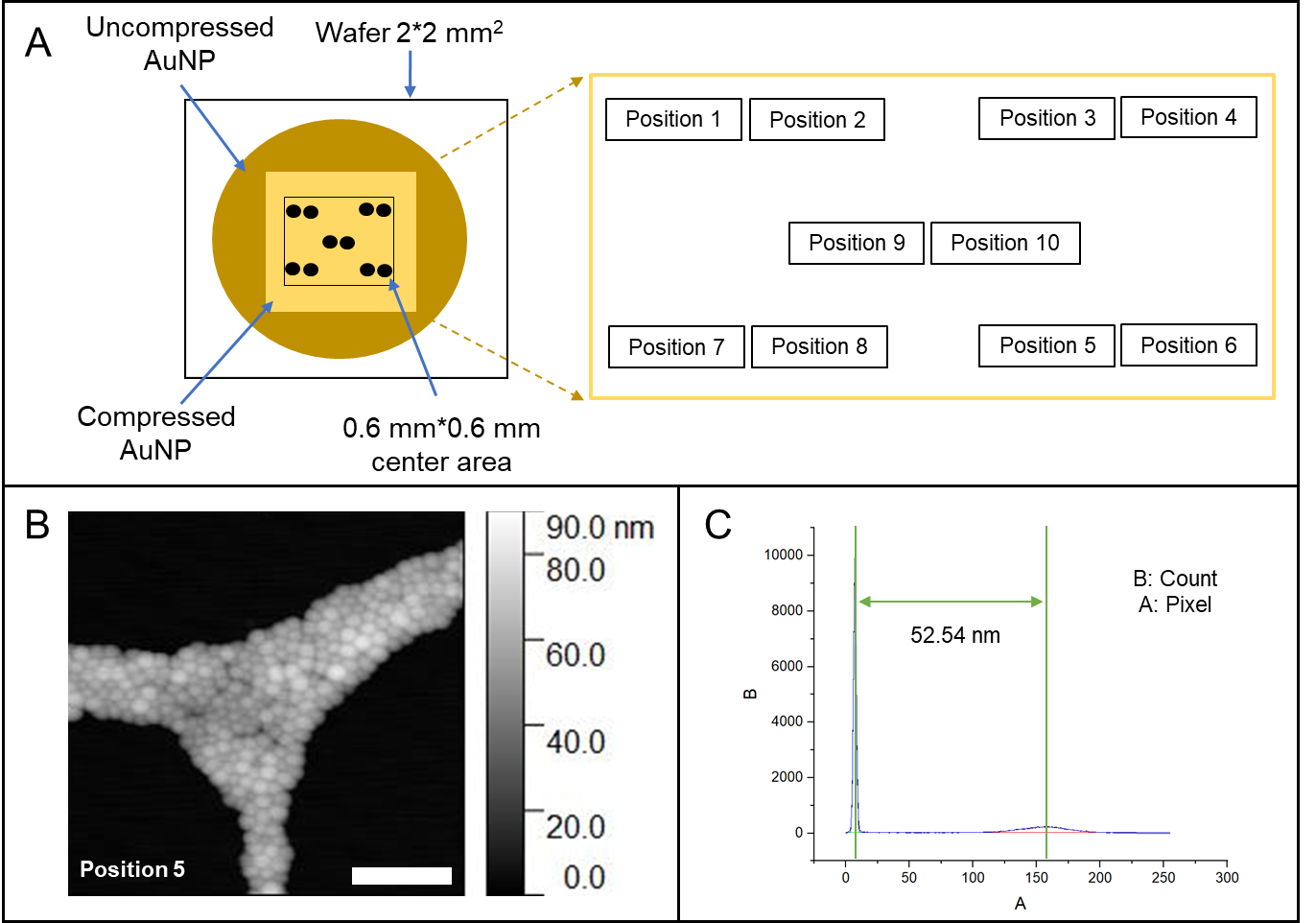


**Fig. S11**. (A) Schematic of ten sampling positions across the substrate for statistical quantification of thickness evolution of the AuNS via compression. B) AFM map of medium AuNSs with 5k PEG from position 5. C) Histogram extraction and Gaussian fit to statistically determine the thickness profile of the as-assembled medium AuNSs 5k PEG for position 5.


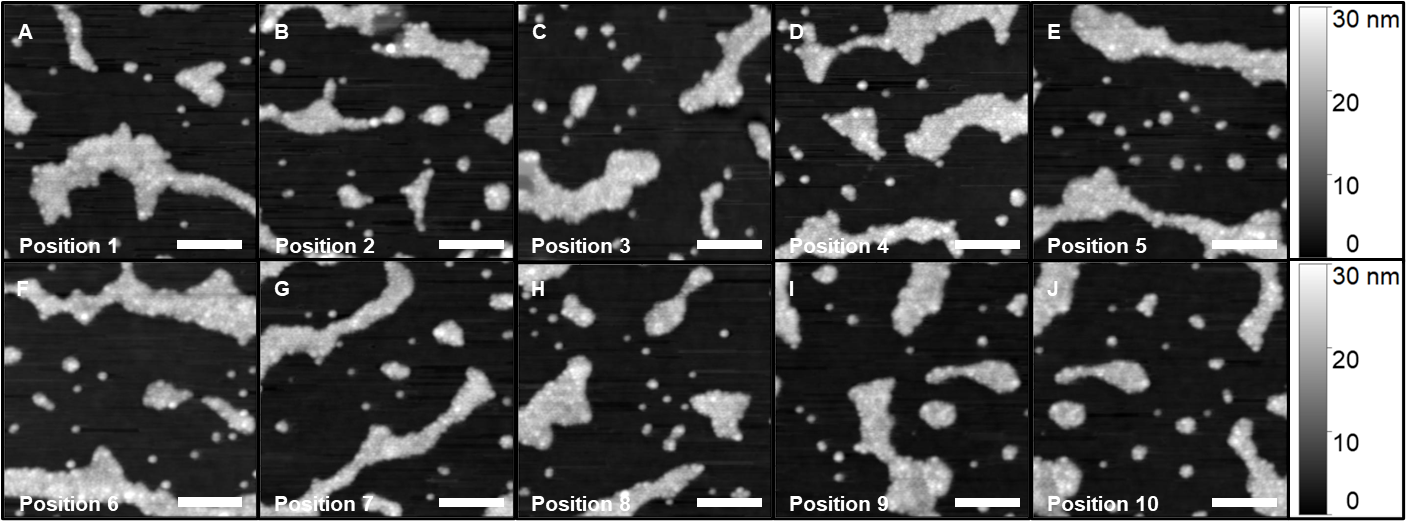


**Fig. S12.** A) - J) AFM maps of as-assembled small AuNSs with 5k PEG. All scale bars are 520 nm.


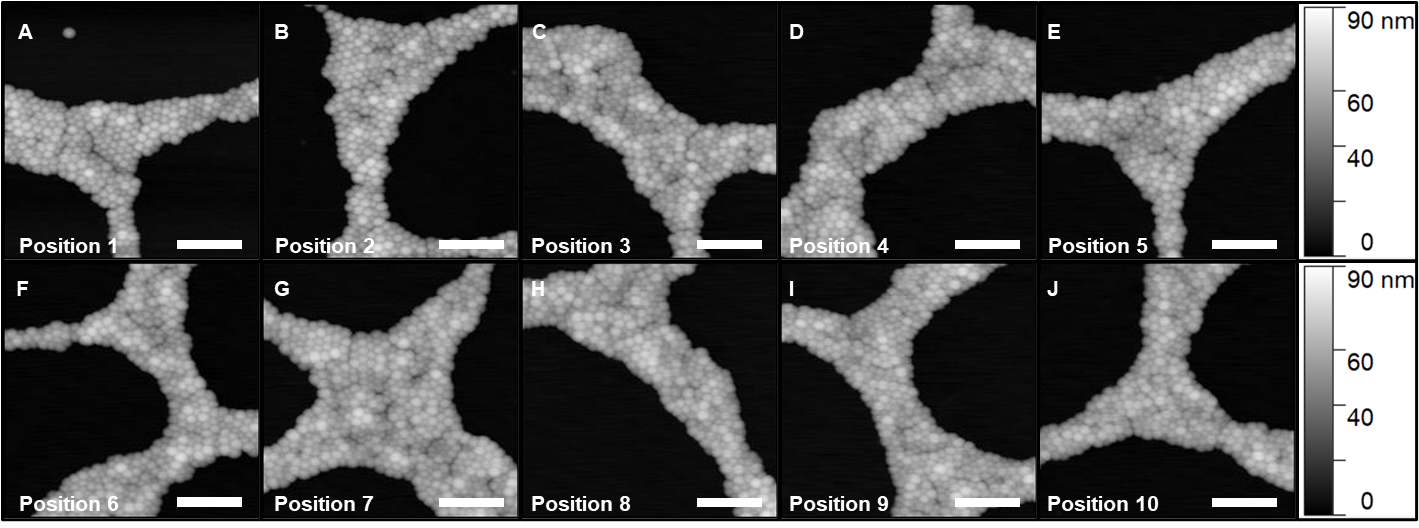


**Fig. S13.** A) - J) AFM maps of as-assembled medium AuNSs with 5k PEG. All scale bars are 520 nm.


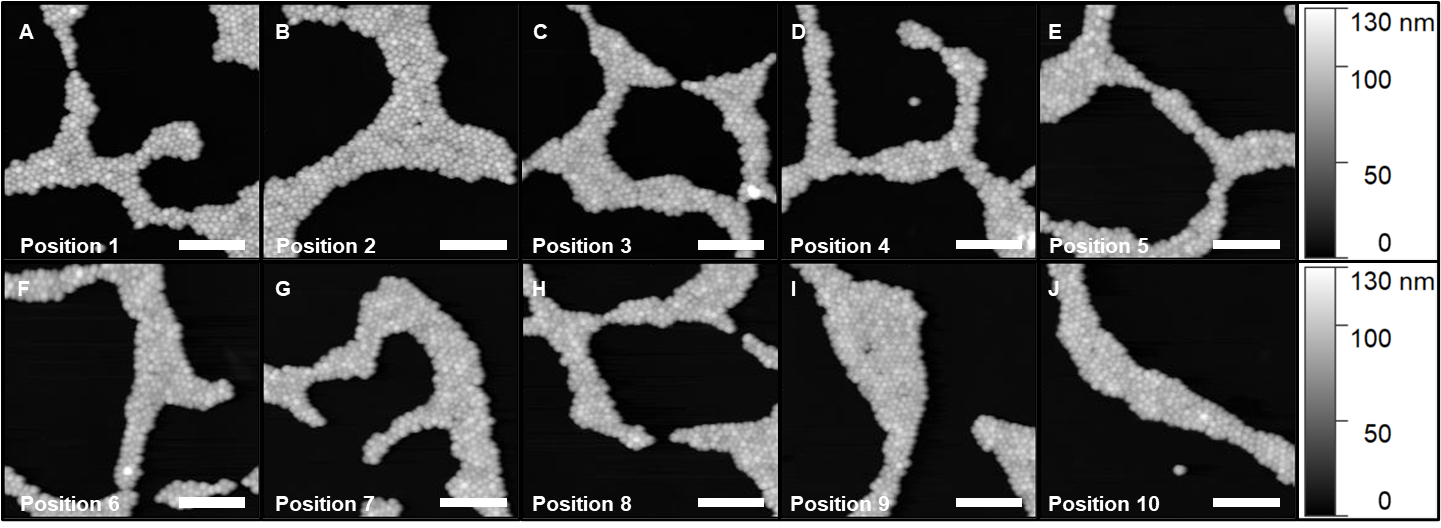


**Fig. S14.** A) - J) AFM maps of as-assembled large AuNS with 5k PEG. All scale bars are 520 nm.


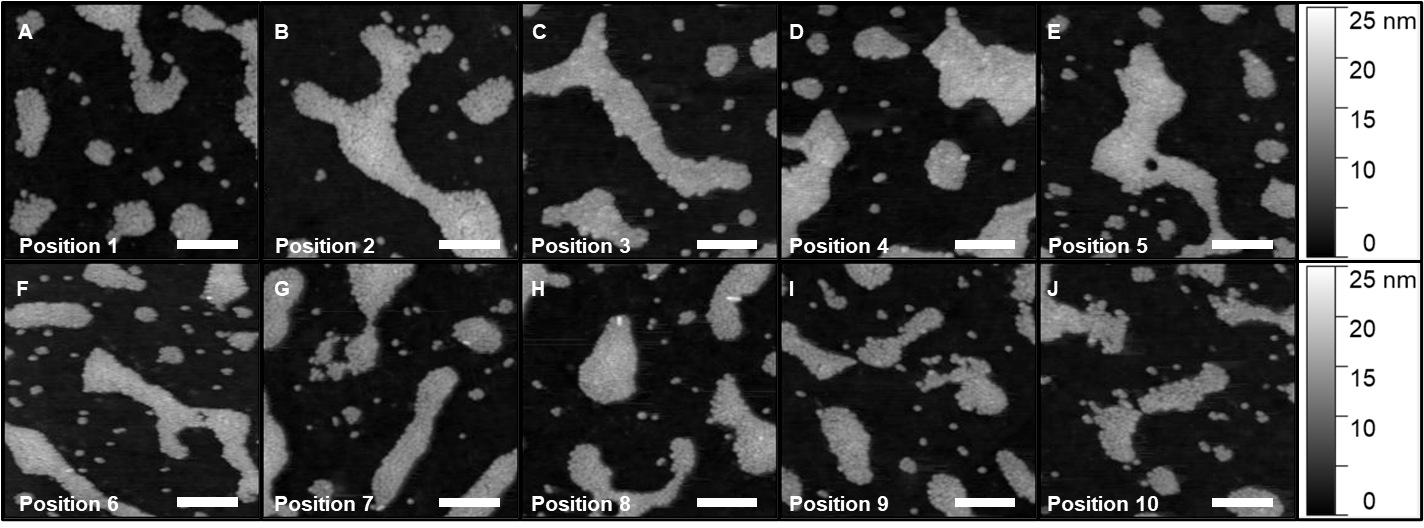


**Fig. S15.** A) - J) AFM maps of compressed small AuNS with 5k PEG. All scale bars are 520 nm.


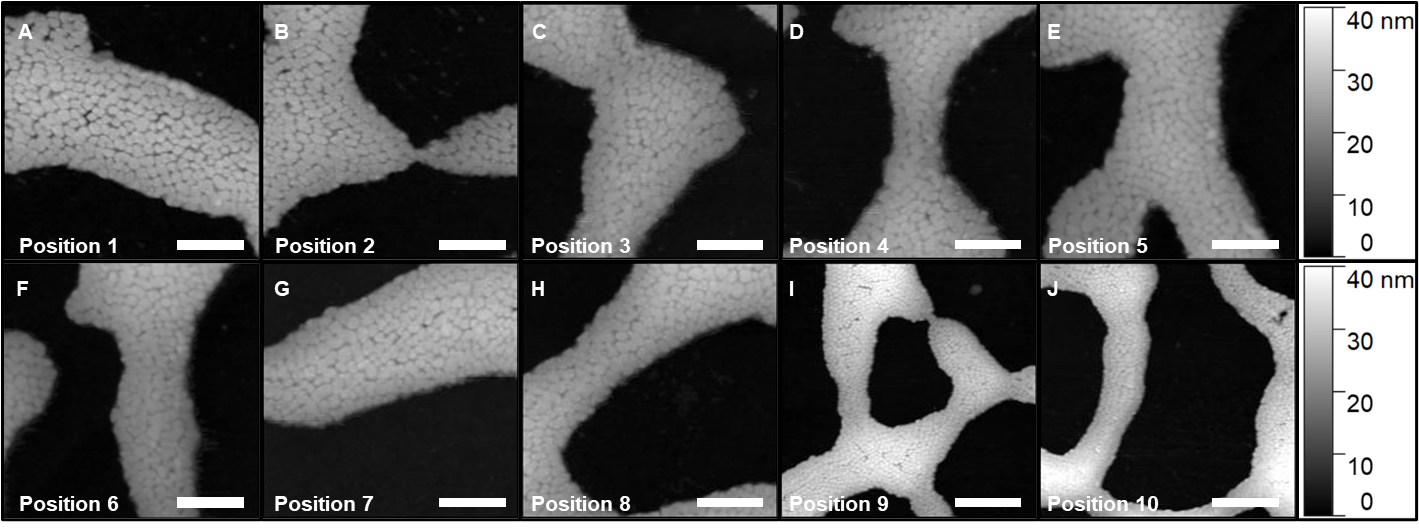


**Fig. S16.** A) - J) AFM maps of compressed medium AuNS with 5k PEG. All scale bars are 520 nm.


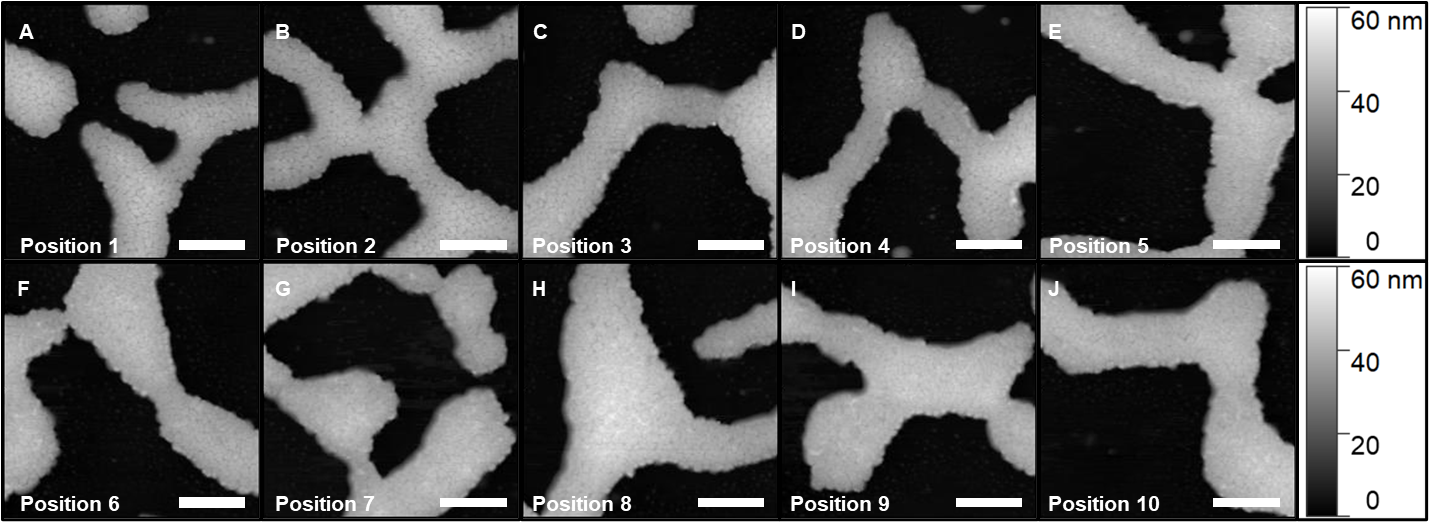


**Fig. S17.** A) - J) AFM maps of compressed large AuNS with 5k PEG. All scale bars are 520 nm.

**Hydrodynamic Length Estimation of PEG (5k and 40k) on small AuNS**


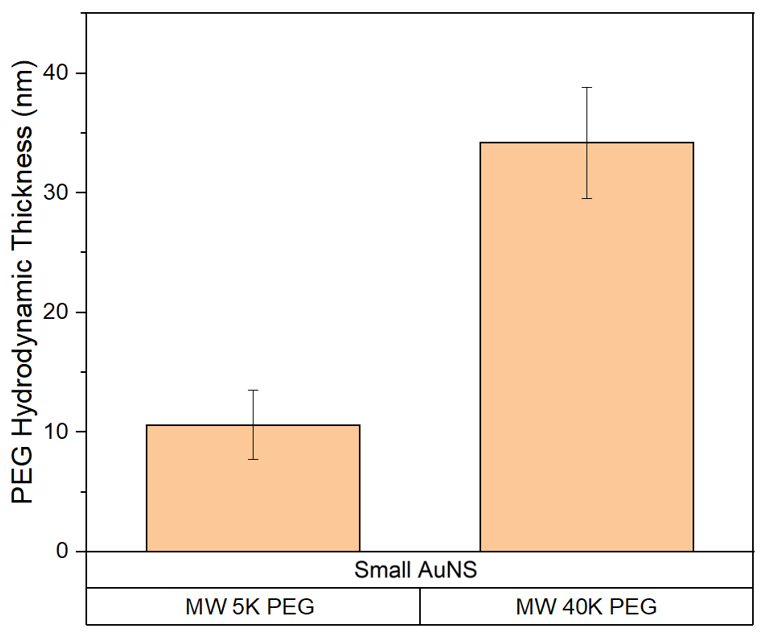


**Fig. S18.** Estimation of hydrodynamic length of PEG on small AuNSs, indicating significantly larger length for 40k PEG compared to 5k PEG.

**The morphology of as-assembled and compressed small AuNS with 40k PEG**

**
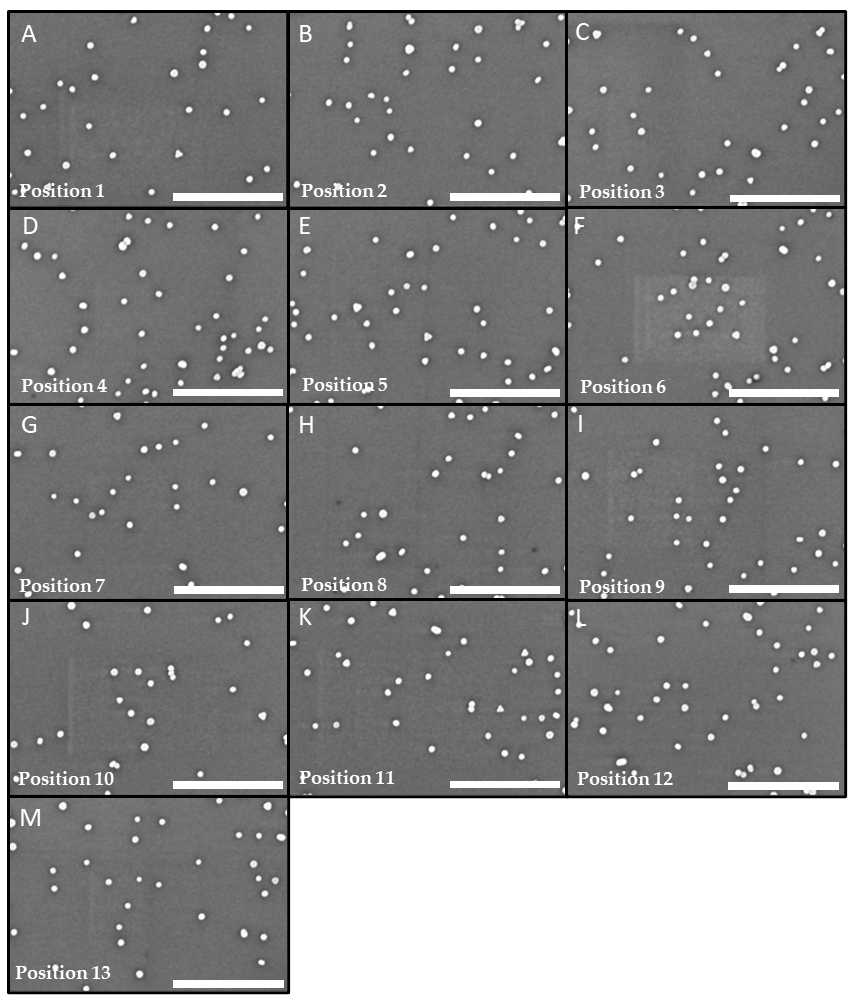
**

**Fig. S19.** A) - M) SEM images of as-assembled small AuNS with 40k PEG on silicon substrate from position 1 to position 13. All scale bars are 500 nm.


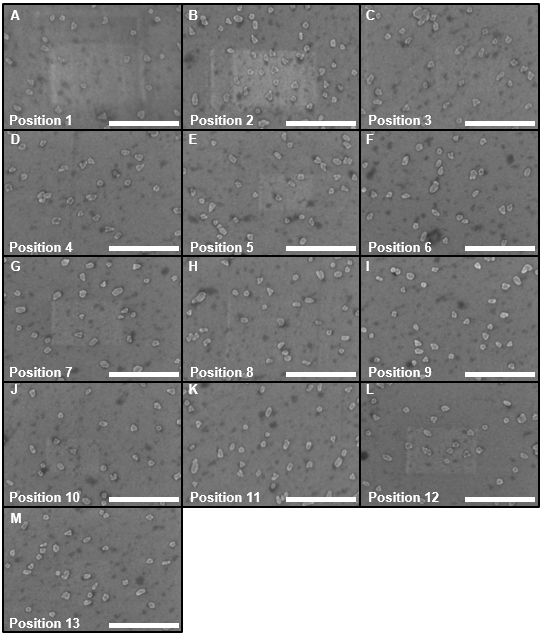


**Fig. S20**. A) - M) SEM images of compressed small AuNS with 40k PEG on silicon substrate from position 1 to position 13. All scale bars are 500 nm.

**Metrology analysis of small AuNS with 40k PEG**

Metrology analysis of individual as-assembled and 3.6 GPa (applied normalized stress) compressed small AuNS with 40k PEG was performed by measuring the in-plane lateral projected 2D area. The goal of the metrology analysis of small AuNS 40k PEG is to understand the induced lateral anisotropy on the nanoparticles when they undergo deformation in isolation from other nanoparticles. Four positions near the central compressed region (positions 10, 11, 12, and 13) were chosen, and SEM images of the positions were analyzed (Figs. S19 and 20). A shape line was drawn along the boundary edges of the as-assembled and compressed AuNSs. Subsequently, the associated AuNS area were measured using ImageJ. Only freely deformed AuNSs were included in the area calculation, while the AuNSs touching the adjacent ones were avoided. The area of the as-assembled and compressed AuNS is 702.5 ± 24.7 nm^2^ and 1337.9 ± 74.6 nm^2^, respectively, indicating ~1.9 times lateral area increment due to compression (Fig. S21).


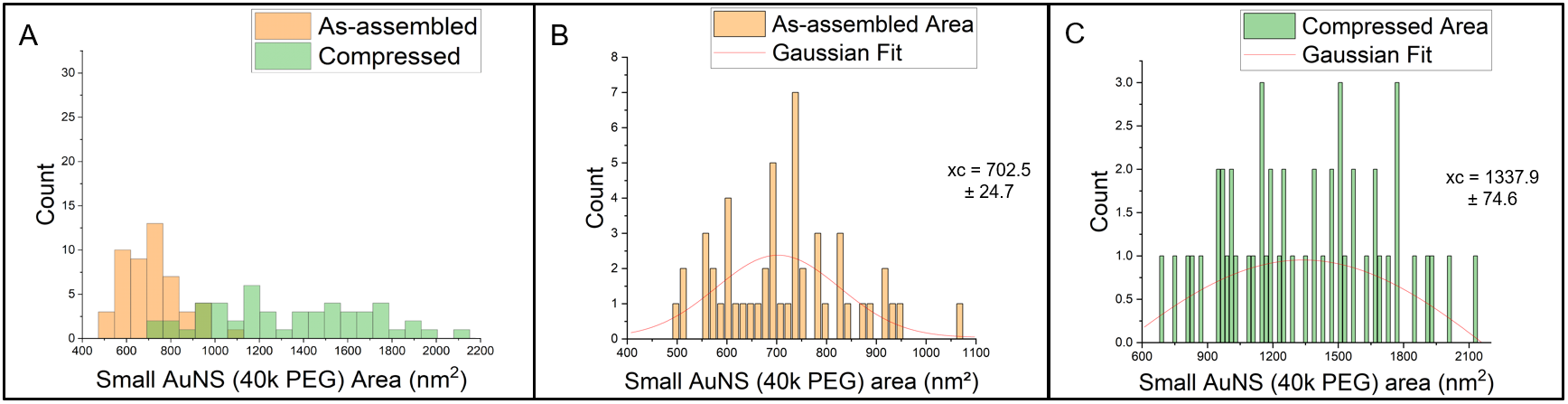


**Fig. S21**. A) Graphical representation of area occupied by the as-assembled and compressed small AuNS with 40k PEG, which is B) 702.5 ± 24.7 nm^2^ and C) 1337.9 ± 74.6 nm^2^, respectively (xc refers to the center of the peak for Gaussian fitting). As can be seen, there is a ~1.9 times increment of the lateral area due to compression.

**Characterization of AuNS (40k PEG) Morphology and Its 2D Transformation**

**
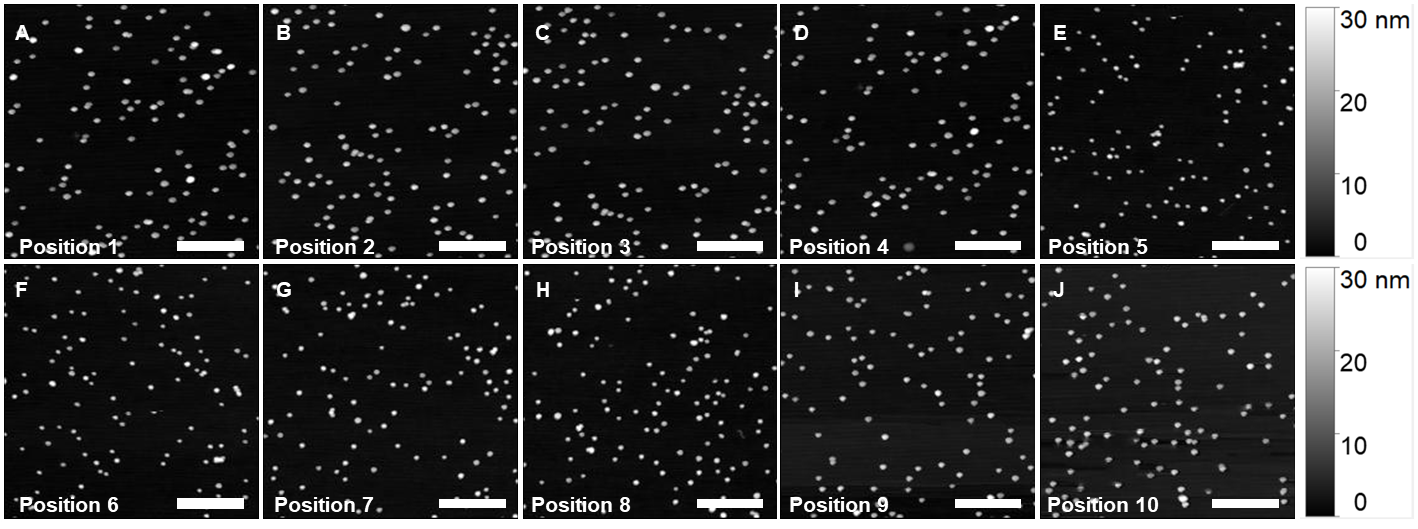
**

**Fig. S22.** A) - J) AFM maps of as-assembled small AuNS with 40k PEG. All scale bars are 520 nm.

**
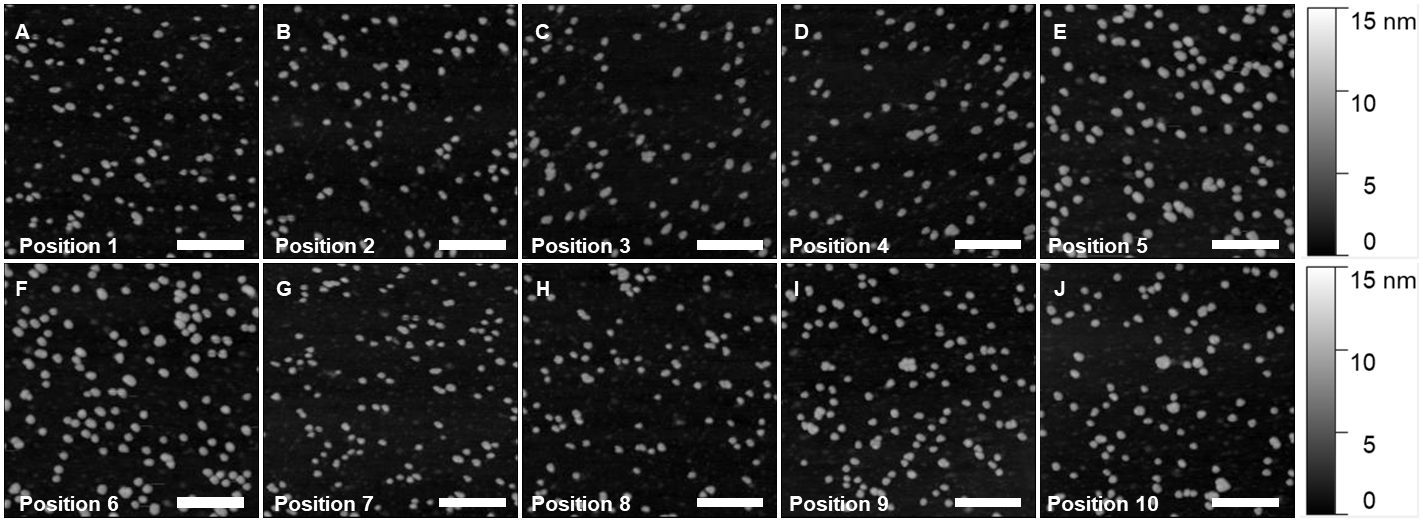
**

**Fig. S23.** A) - J) AFM maps of compressed small AuNS with 40k PEG. All scale bars are 520 nm.

**The morphology of as-assembled and compressed large AuNS with 40k PEG**


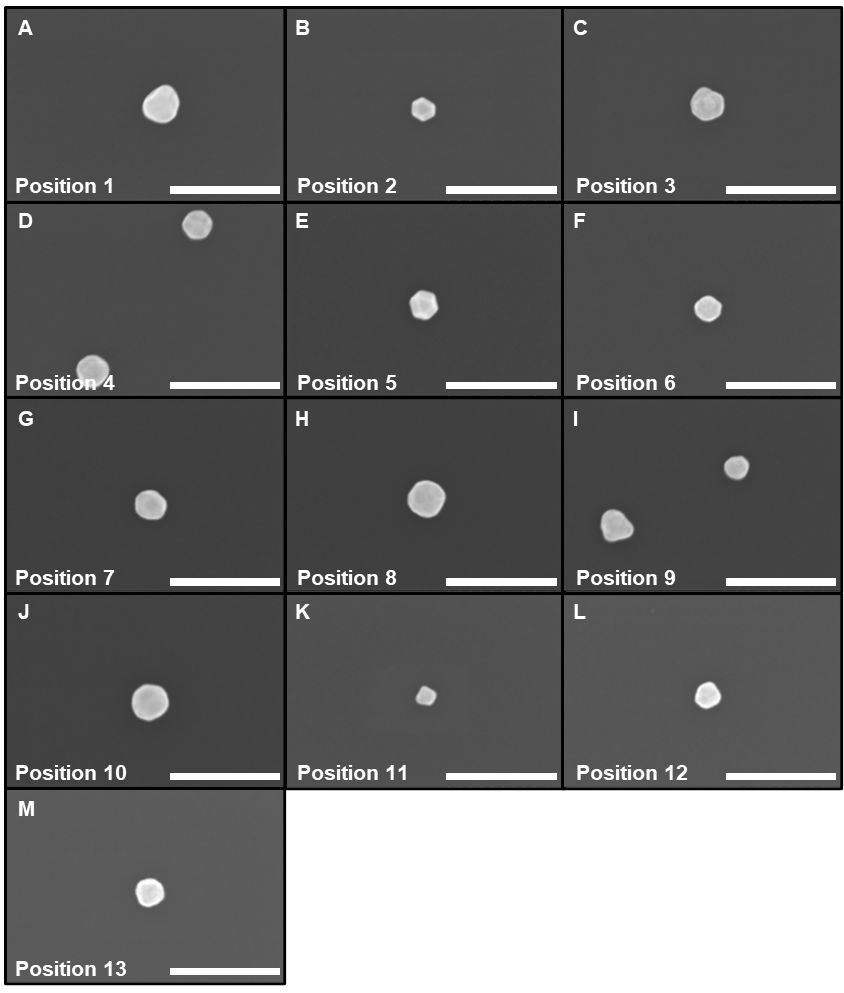


**Fig. S24.** A) - M) SEM images of as-assembled isolated large AuNSs with 40k PEG from position 1 through position 13. All scale bars are 500 nm.

**
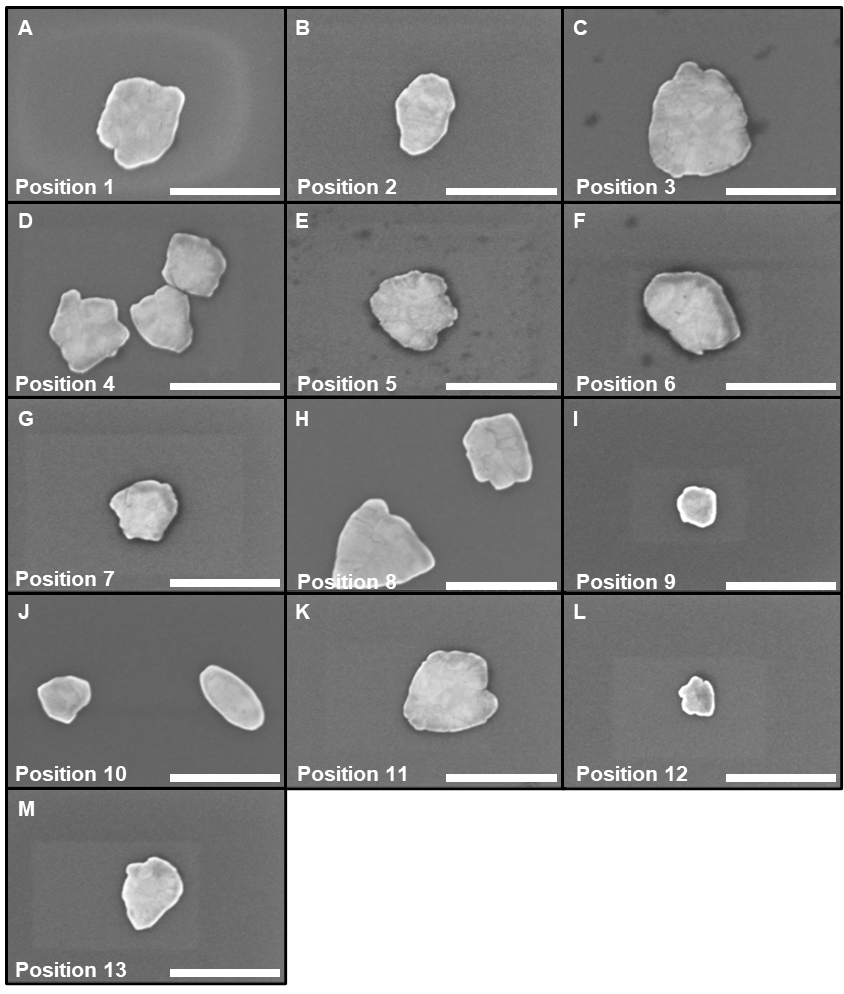
**

**Fig. S25.** A) - M) SEM images of compressed isolated large AuNSs with 40k PEG from position 1 through position 13. All scale bars are 500 nm.


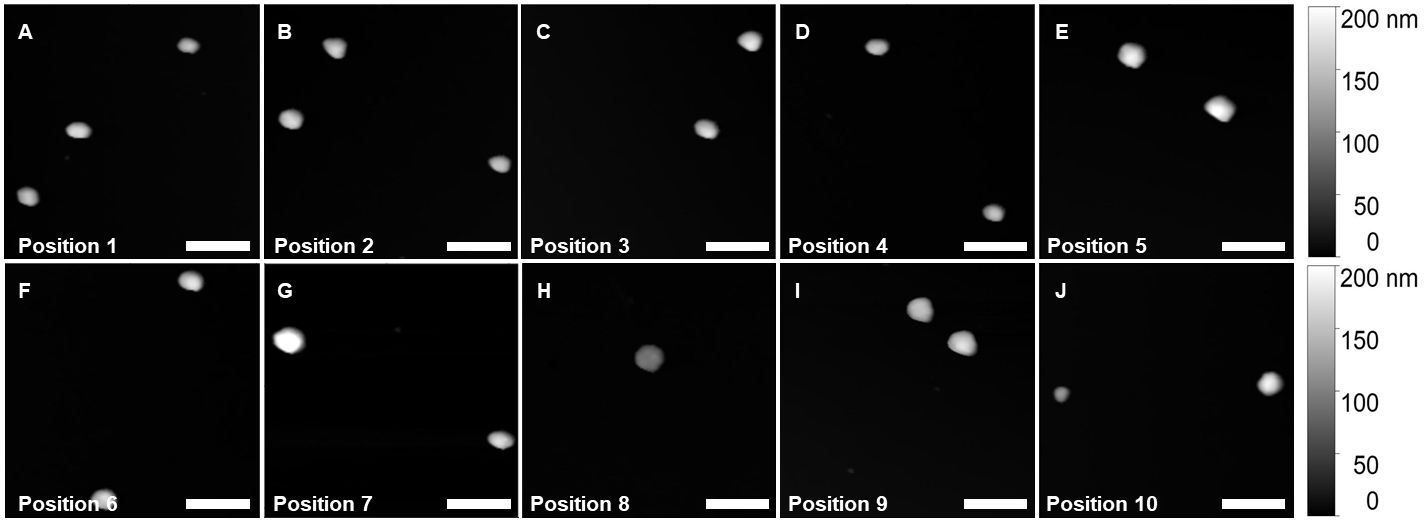


**Fig. S26.** A) - J) AFM maps of as-assembled isolated large AuNS with 40k PEG. All scale bars are 500 nm.


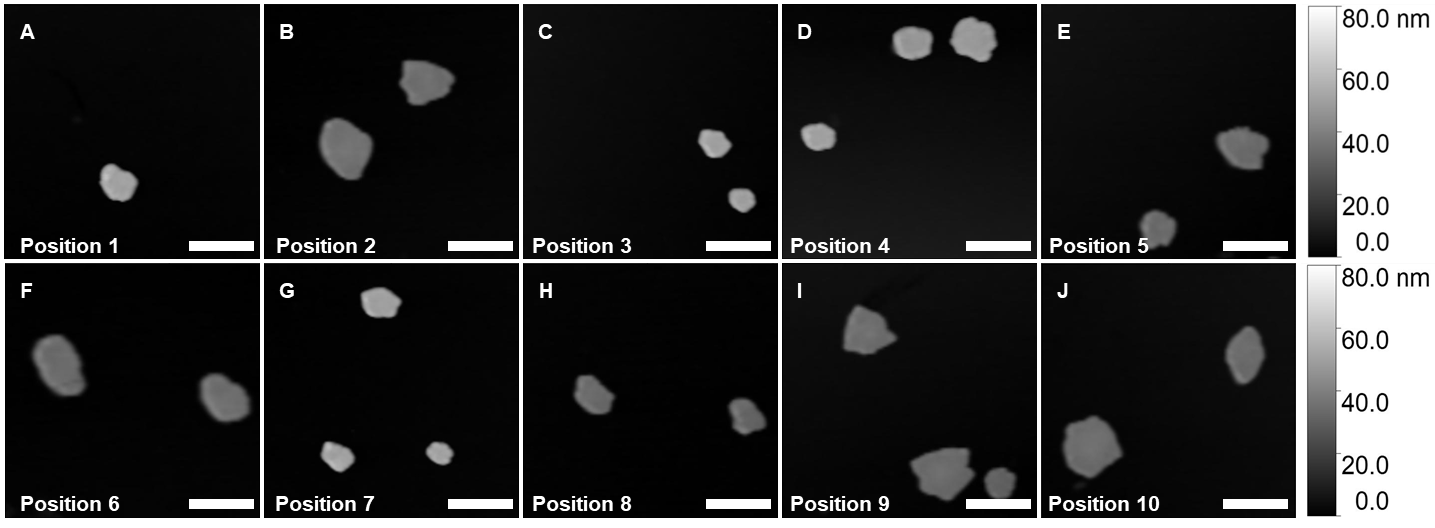


**Fig. S27.** A) - J) AFM maps of compressed isolated large AuNS with 40k PEG. All scale bars are 500 nm.

**
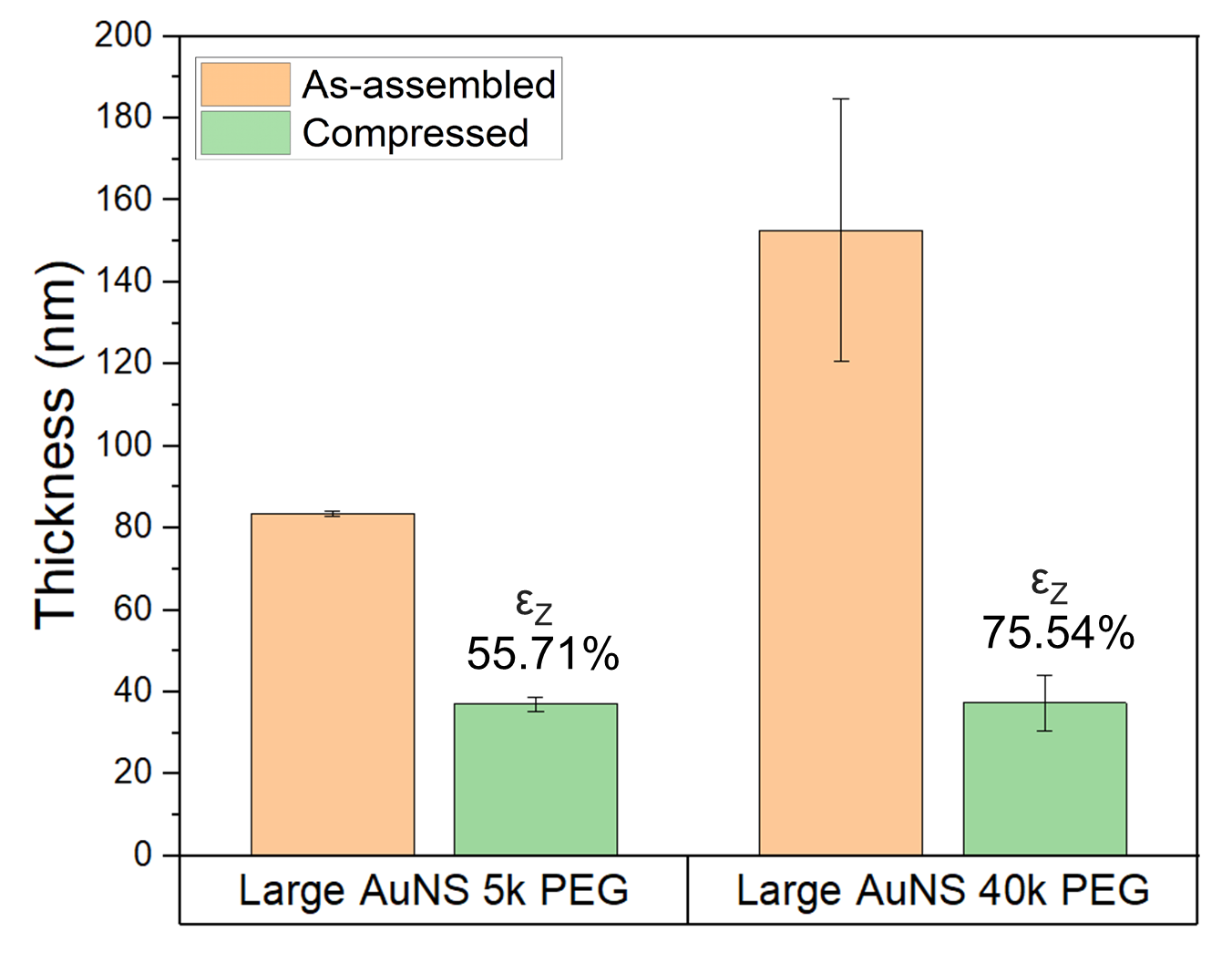
**

**Fig S28.** Thickness evolution and z-strain from the as-assembled to the compressed large AuNS with 5k PEG (close-packed) and 40k PEG (isolated). The represented data indicate an induced vertical out-of-plane z-strain (ε_Z_) of 55.71 ± 0.1% (from 83.6 ± 0.7 nm to 37.0 ± 1.7 nm) for large AuNS with 5k PEG and 75.54 ± 0.1% (from 152.7 ± 32.1 nm to 37.3 ± 6.9 nm) for large AuNS with 40k PEG under applied normalized compressive stress.

**Transformation of close-packed AuNSs with progressively larger compressive stresses: nanoscale goldbeating.**

The applied normalized stress dictates the post-compression morphology of the AuNSs. We demonstrate that close-packed AuNSs can be compressed repeatedly with progressively higher applied normalized stresses, which emulates the ancient craft of goldbeating. Figure S29 shows the morphological evolution of close-packed large AuNSs with 5k PEG from as-assembled, then after applied normalized stress of σ_norm 1_ = 1 GPa, and then again after a larger applied normalized stress of σ_norm 2_ = 2.15 GPa.


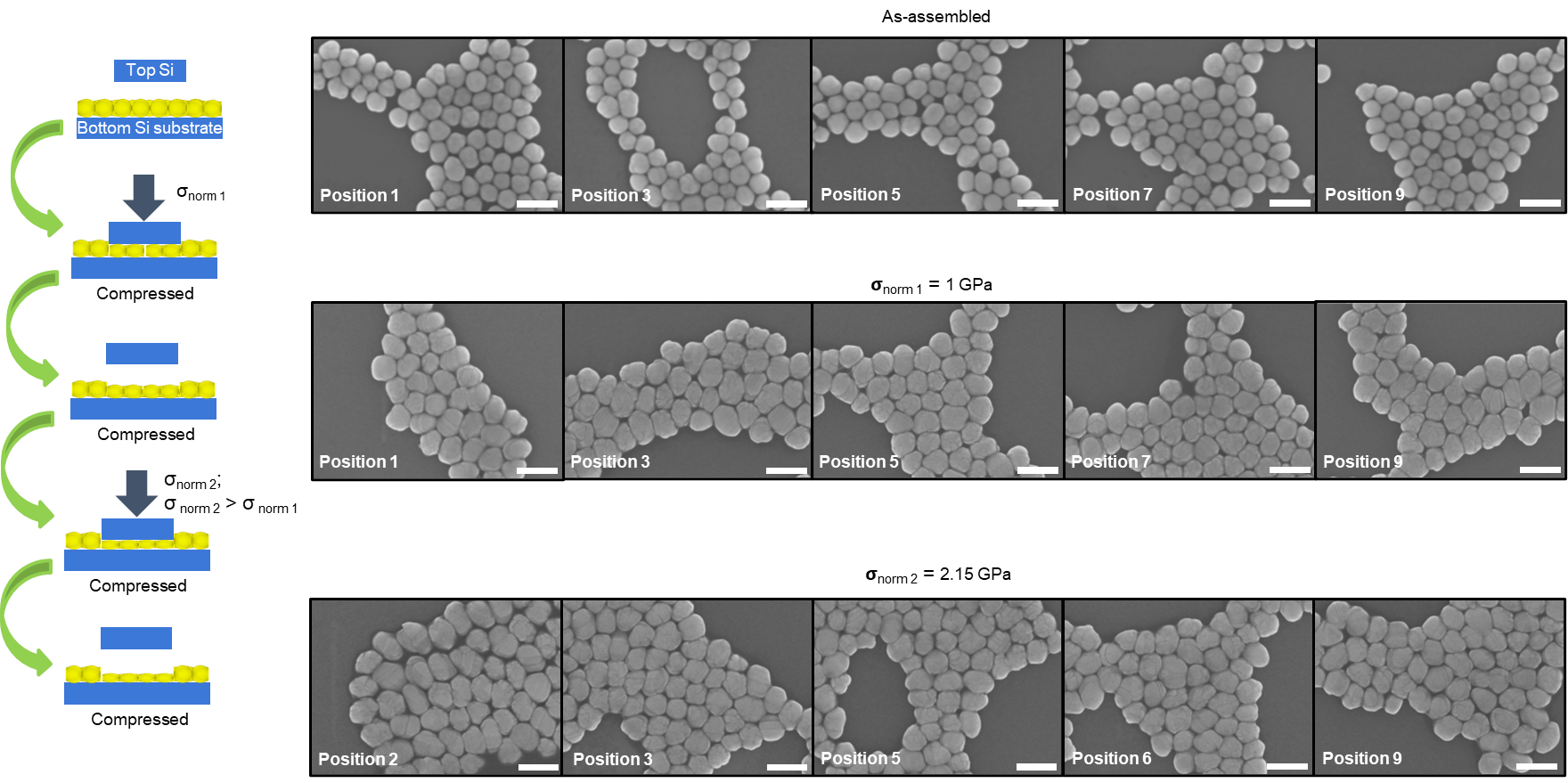


**Fig. S29.** Schematic and SEM images (at five different positions on the substrate) of repeatedly compressed close-packed large AuNSs with 5k PEG with progressively larger applied normalized stresses, emulating the conventional goldbeating process and introducing concomitant progressive reshaping and thinning of the AuNSs. The as-assembled, close-packed large AuNSs with 5k PEG were first compressed under σ_norm 1_ = 1GPa, and then subsequently compressed again under σ_norm 2_ = 2.15 GPa, where the progressive reshaping or goldbeating is evident from the SEM images. All scale bars are 200 nm.

**The morphology of as-assembled and compressed large AuNS 40k PEG at a larger 𝞂_norm_**


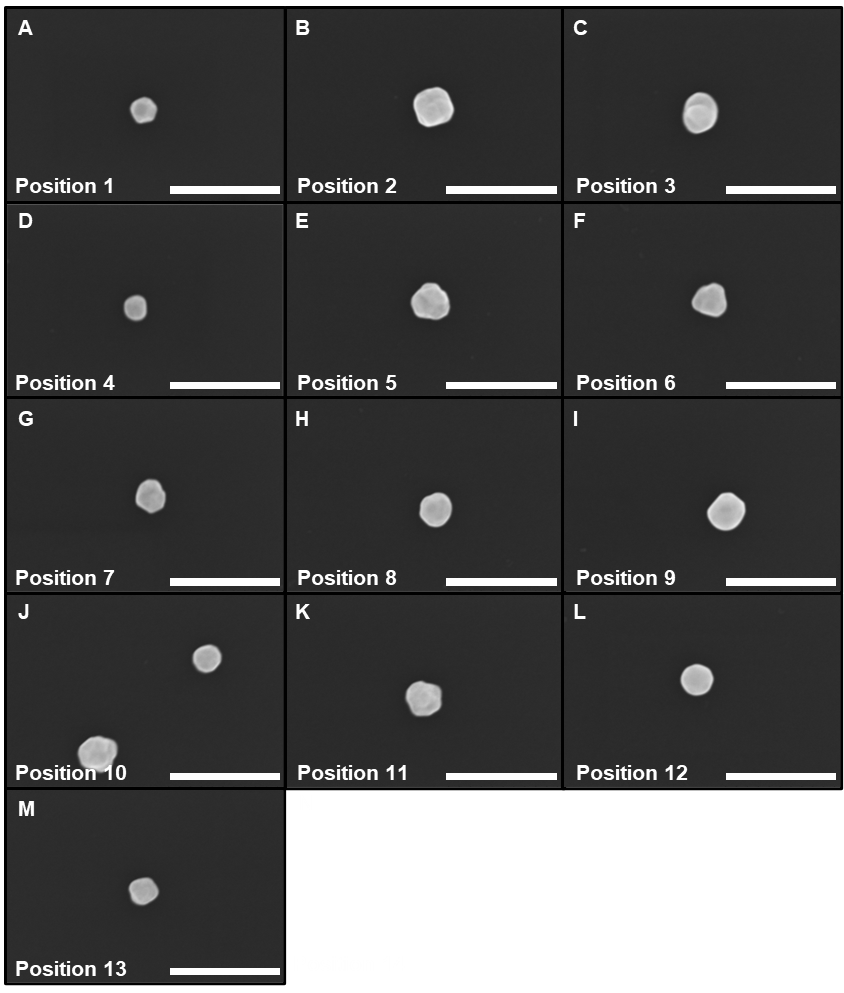


**Fig. S30.** A) - M) SEM images of as-assembled isolated large AuNS with 40k PEG from position 1 through position 13. All scale bars are 500 nm.

**
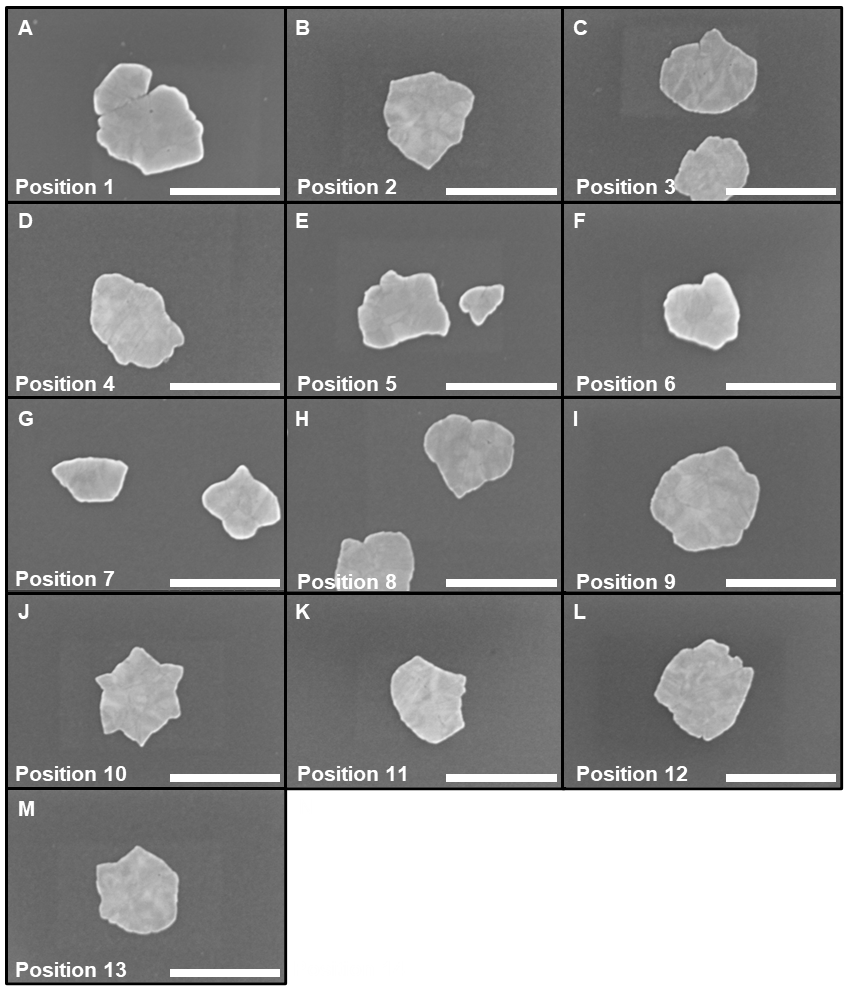
**

**Fig. S31.** A) - M) SEM images of compressed (𝞂_norm_ = 6 GPa) isolated large AuNS with 40k PEG from position 1 through position 13. The average ε_Z_ = 80.16 ± 0.1%. All scale bars are 500 nm.


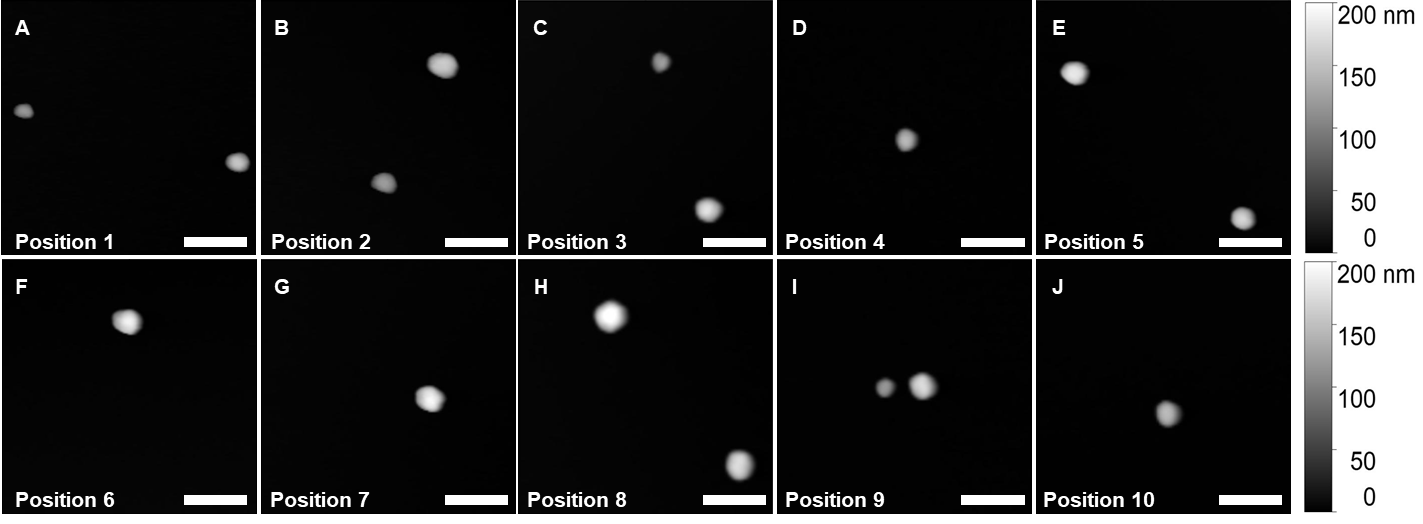


**Fig. S32.** A) - J) AFM maps of as-assembled large AuNS with 40k PEG. All scale bars are 500 nm.


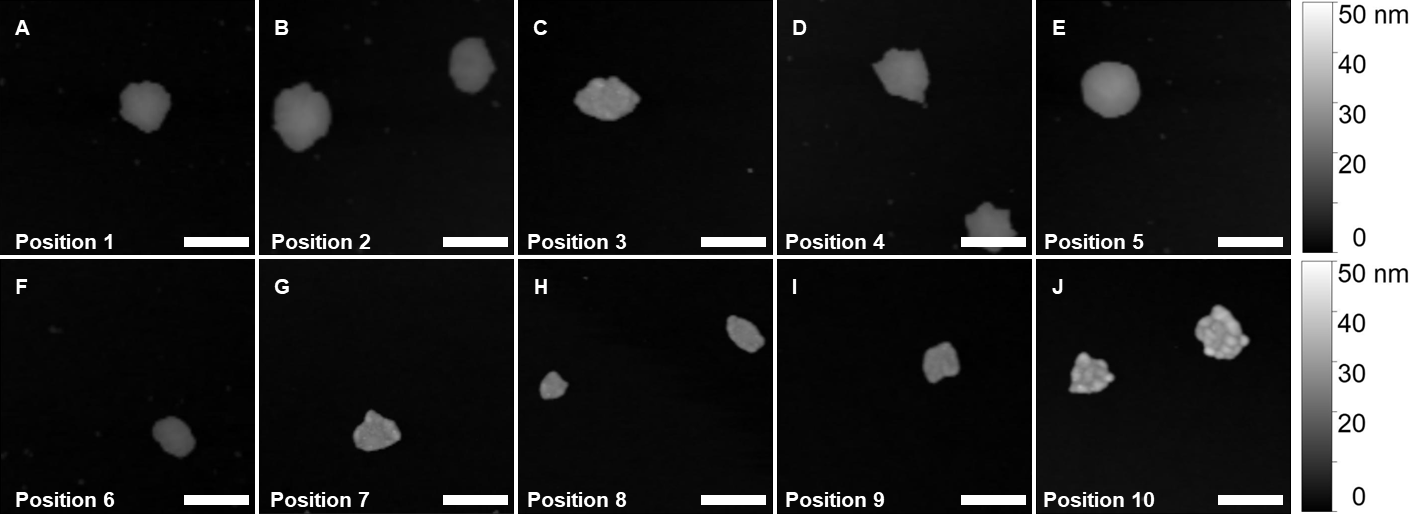


**Fig. S33.** A) - J) AFM maps of compressed (𝞂_norm_ = 6 GPa) isolated large AuNS with 40k PEG. The average ε_Z_ = 80.16 ± 0.1%. All scale bars are 500 nm.


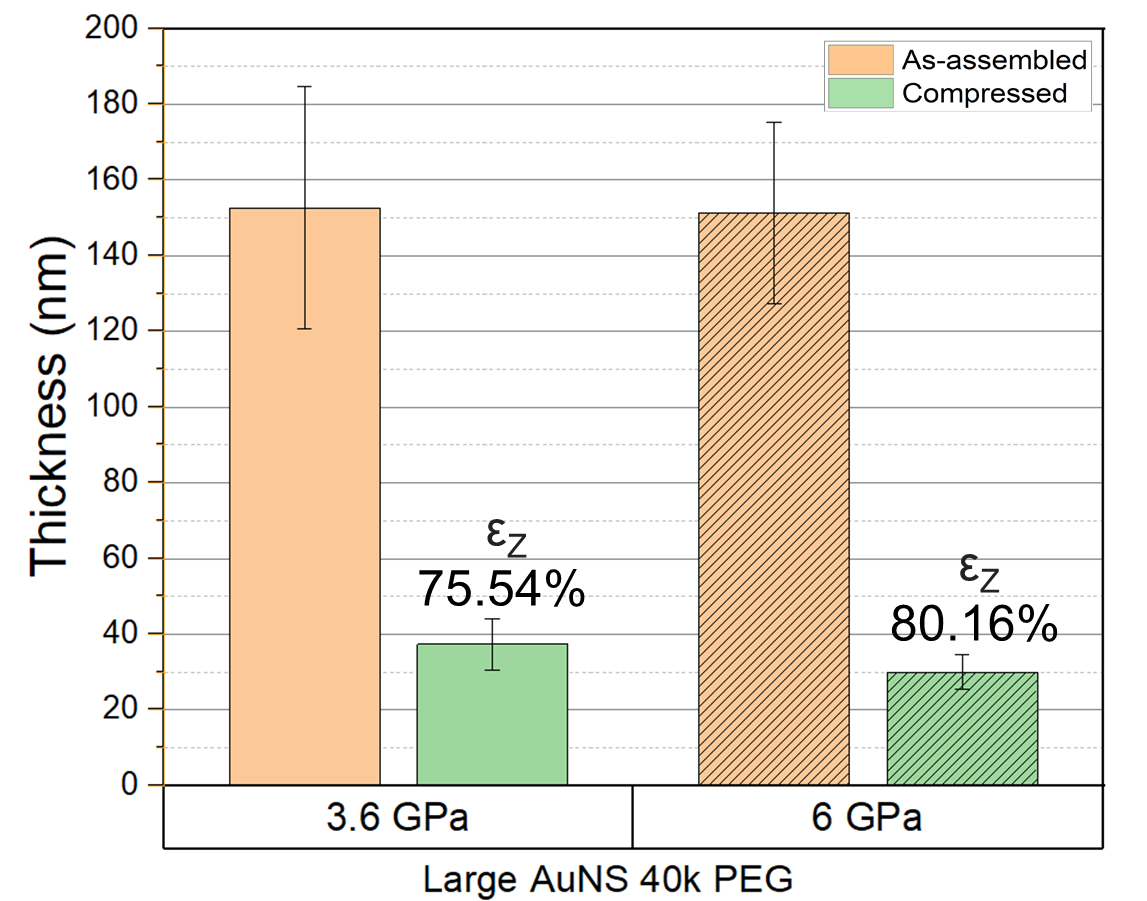


**Fig S34.** Thickness evolution and z-strain from the as-assembled, isolated large AuNS with 40k PEG to post-compression at 𝞂_norm_ = 3.6 GPa and 𝞂_norm_ = 6 GPa. The represented data indicate an induced vertical out-of-plane z-strain (ε_Z_) of 75.54 ± 0.1% (from 152.7 ± 32.1 nm to 37.3. ± 6.9 nm) for isolated large AuNS with 40k PEG under 𝞂_norm_ = 3.6 GPa versus ε_Z_ of 80.16 ± 0.1% (from 151.4 ± 24.0 nm to 30.0 ± 4.6 nm) for large isolated AuNS with 40k PEG under a higher 𝞂_norm_ = 6 GPa.

**Molecular Dynamics Simulation of AuNS**

To study the role of the compression velocity of the top compression surface in the deformation behavior of the AuNS, we also selected compression velocity to be v=0.005 nm/ps, a higher velocity, and run a few more cases with different initial equilibrium configurations. The true yield stress is listed in Table S5. The true yield stress evaluated in the MD simulation relates to the compression velocity selections. Even with a higher compression velocity of 0.005 nm/ps, it was found that the average yield stress was within 3% of the yield stress calculated at a low strain rate. However, it was found that the variation in yield stress with a slower compression velocity was less compared to a higher velocity. Therefore in this study, a slower velocity of 0.001 nm/s was adopted. Even though the activated slipping directions are different based on thermal vibrations, all the deformed AuNSs appear to have morphology consistent with the deformed AuNSs in the SEM images shown in Fig. 3. Top view of the compressed AuNS at ~38% strain. The atoms are colored using dislocation analysis (DXA) in OVITO. Yellow represents the FCC structure, red represents the HCP structure, and surface atoms are removed for better visualization.

**The morphology of as-assembled and compressed AuNR**


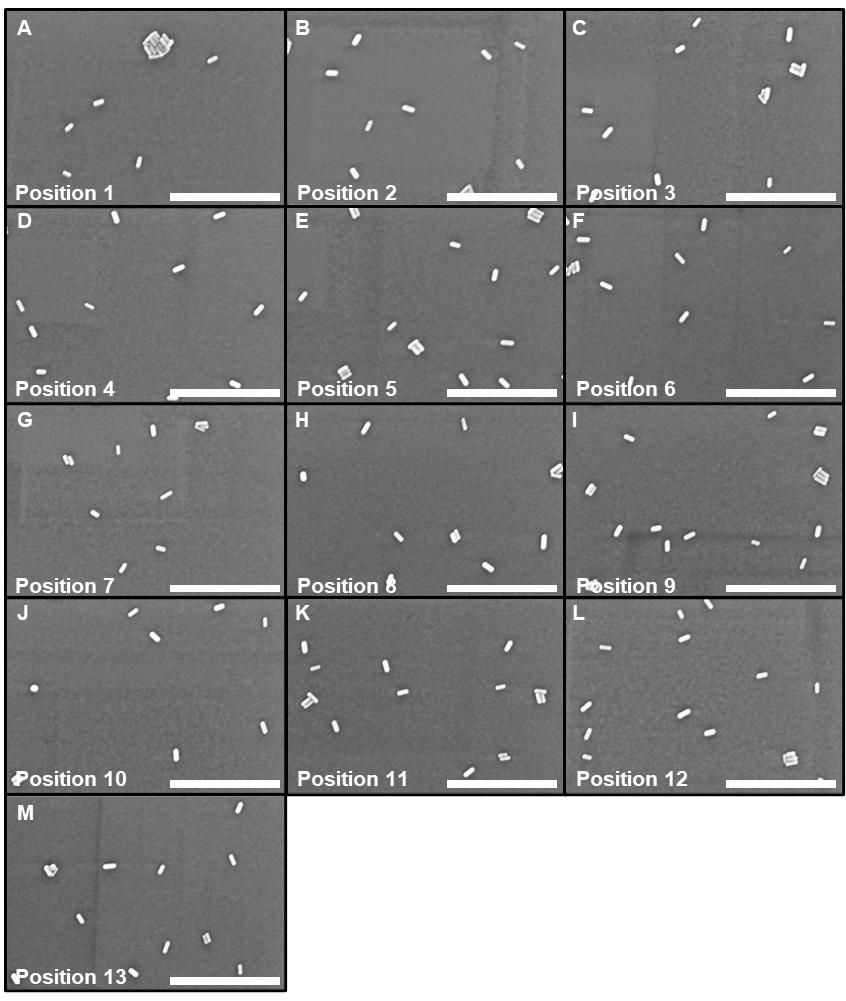


**Fig. S35.** SEM images of as-assembled short AuNR with 5k PEG on silicon wafer from position 1 to position 13. All scale bars are 500 nm.


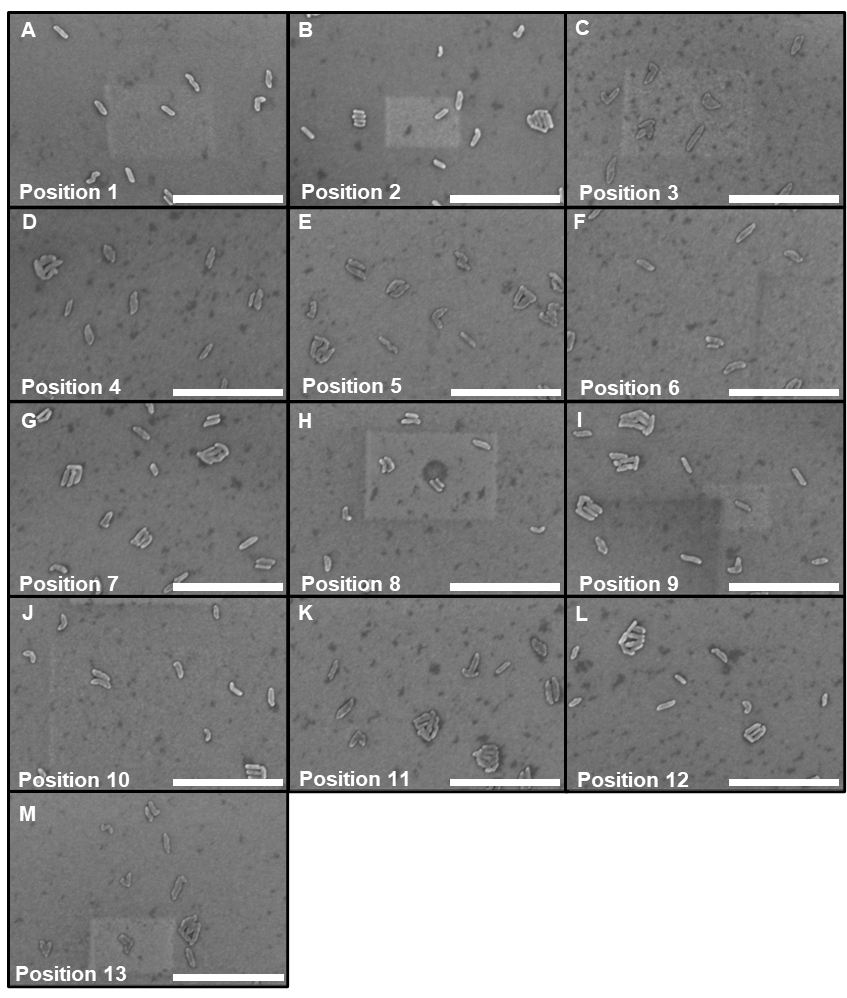


**Fig. S36**. SEM images of compressed short AuNR with 5k PEG on silicon wafer from position 1 to position 13. All scale bars are 500 nm.

**
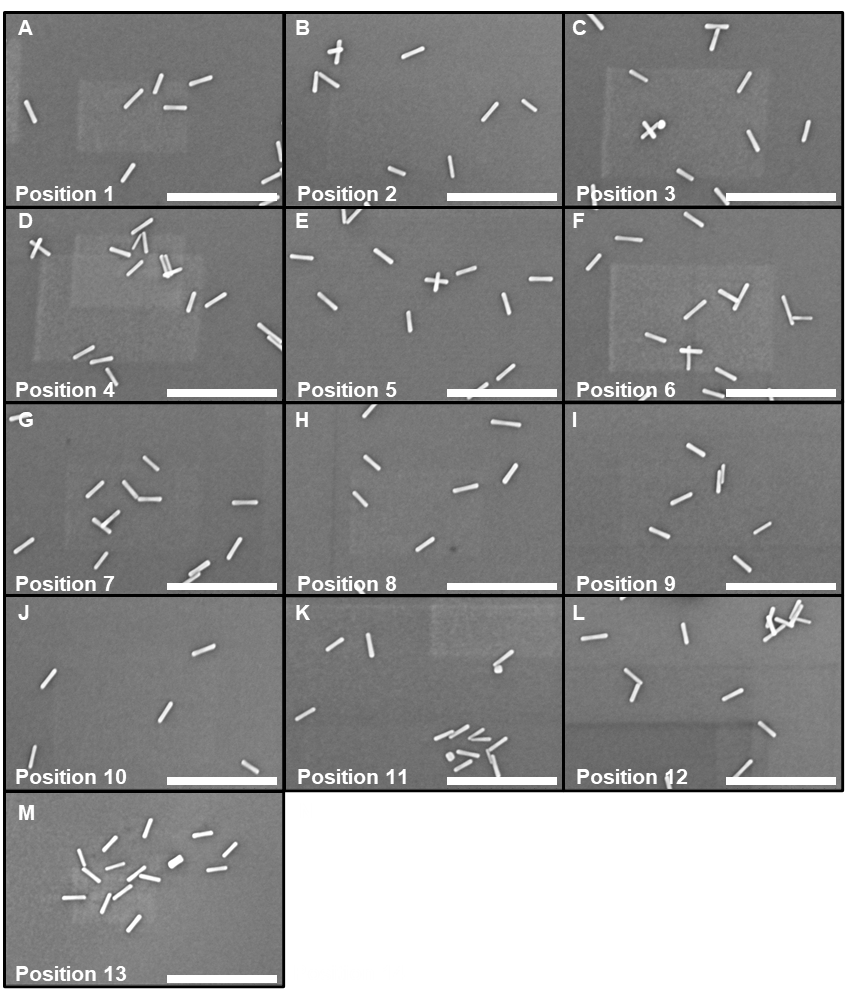
**

**Fig. S37**. SEM images of as-assembled long AuNR with 5k PEG on Si wafer from position 1 to position 13. All Scale bars are 500 nm.

**
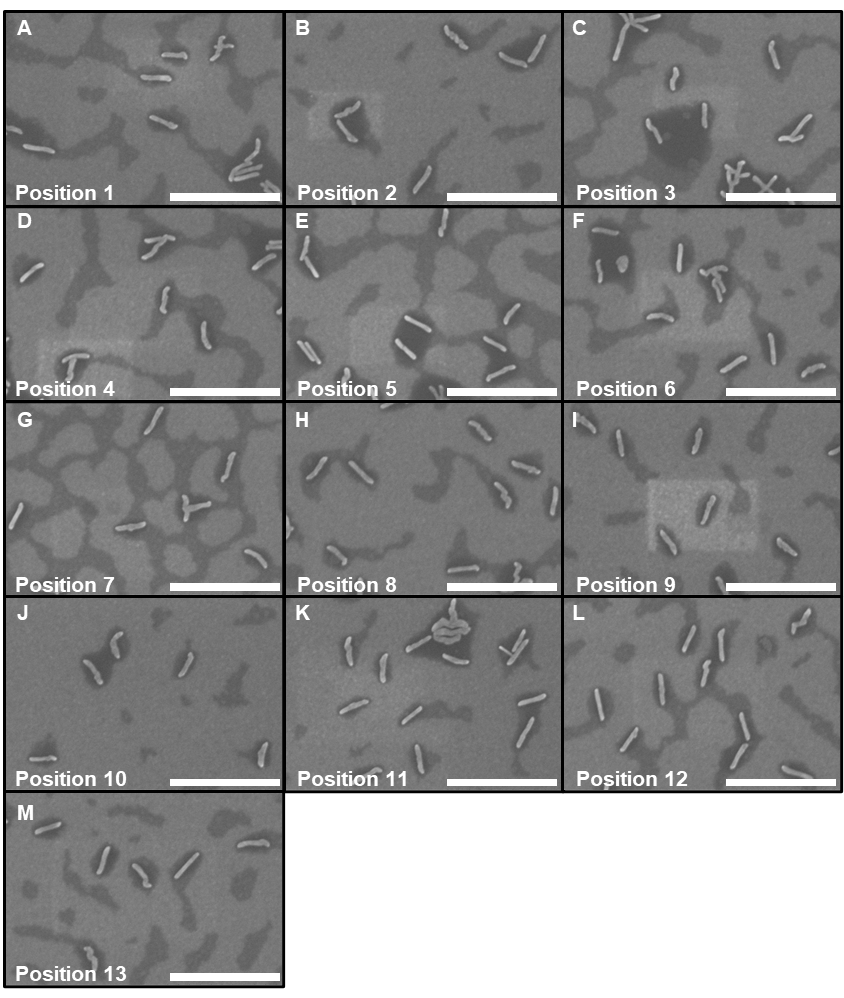
**

**Fig. S38**. SEM images of compressed long AuNR with 5k PEG on silicon wafer from position 1 to position 13. All scale bars are 500 nm.

**Metrology analysis of AuNR**

The plastic deformation-driven metrology of the AuNR was characterized by analyzing the as-assembled and post-compression morphology. The area of the AuNRs was estimated by drawing a shape outline meticulously along the AuNR boundary using ImageJ (Fig. S39*A-B*). 100 as-assembled and compressed AuNRs, each, were analyzed across the sample from position 1 to 13, corresponding to the SEM images in Figs. S35-38.

Evident from the SEM images, the compression of the short and long AuNRs results in anisotropic 2D morphology with nonuniform edges in the lateral direction. As such, an end-to-end measurement of the dimensions (i.e., length and width) may result in erroneous estimation. We resort to the ellipse fitting of these non-uniform shapes to estimate the metrological evolution (length - major axis and width - minor axis) during the compression process because the AuNR shape can be closely approximated to be elliptical. While acknowledging that this could result in overestimating the dimensions, we aim to estimate the dimensional change by the ellipse fitting of both as-assembled and compressed AuNRs.

Here, the image analysis of individual AuNRs was performed by fitting an ellipse using ImageJ. An arbitrarily constructed shape with an enclosed profile contains a two-dimensional distribution of data points, which can be fitted by the best representative ellipse using ImageJ. During this attempt toward the elliptical fitting, the area is kept similar (maximum deviation of 4%), while the second-order central moment of both shapes is kept equal (1). A shape outline is drawn along the boundary edge of the AuNRs to assume their anisotropic shape with a closed profile; subsequently, processing of the image with the best-fitted ellipse was performed to find the length (major axis) and width (minor axis) of the as-assembled and compressed AuNR morphology (Fig. S39*D*). SEM images for all the positions (1 to 13) across the samples were used to characterize the as-assembled and compressed AuNRs (Figs. S35-38). Only individual AuNRs were characterized, while the sintered AuNRs were avoided during the analysis. This metrology analysis provides statistical information regarding the change of the length (major axis) and width (minor axis) before and after compression (Figs. S40-43).

**
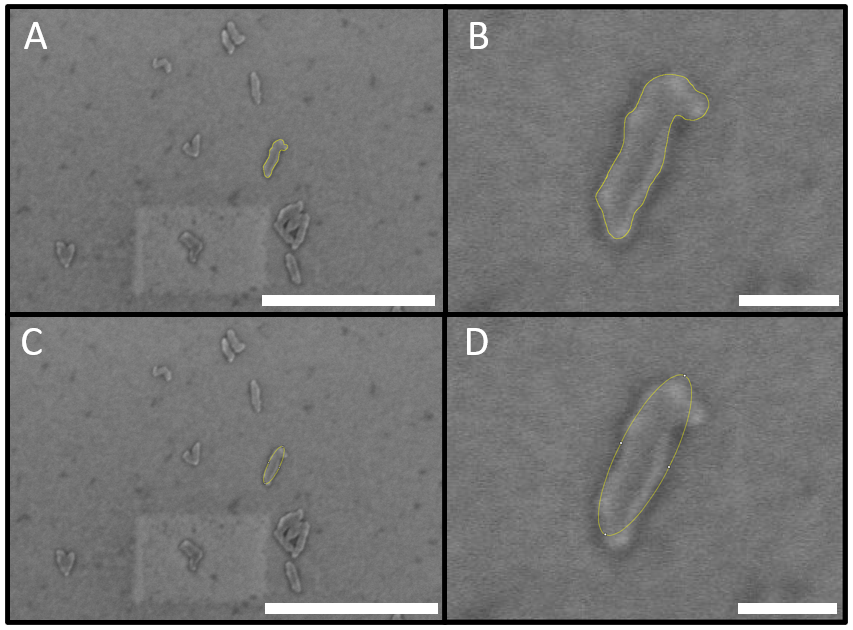
**

**Fig. S39.** A), B) Shape outline drawn along the anisotropic boundary line of the deformed (yellow outlined) small AuNR with 5k PEG (position 13), which was subsequently used for estimation of the area and ellipse fitting. C), D) Ellipse fitting of the shape outline using ImageJ. All scale bars are A), C) 500 nm, and B), D) 50 nm.

**
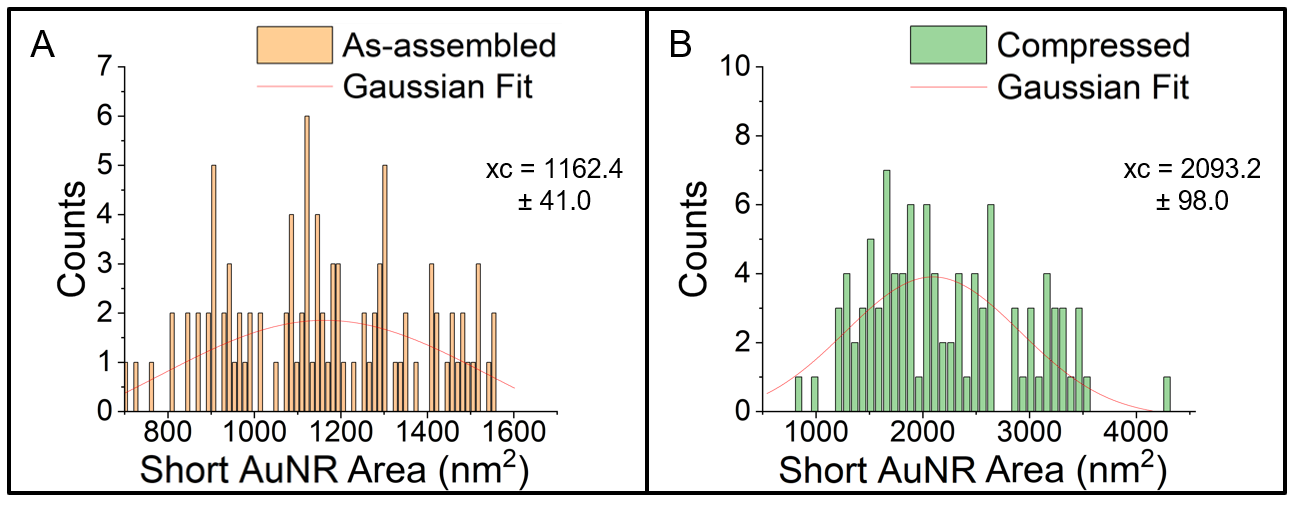
**

**Fig. S40**. Statistical metrology analysis of projected area as-assembled and compressed short AuNRs. A) The projected area of as-assembled short AuNRs was estimated to be 1162.4 ± 41.0 nm^2^. B) After compression, the area increased to 2093.2 ± 98.0 nm^2^ (an 80% change).

**
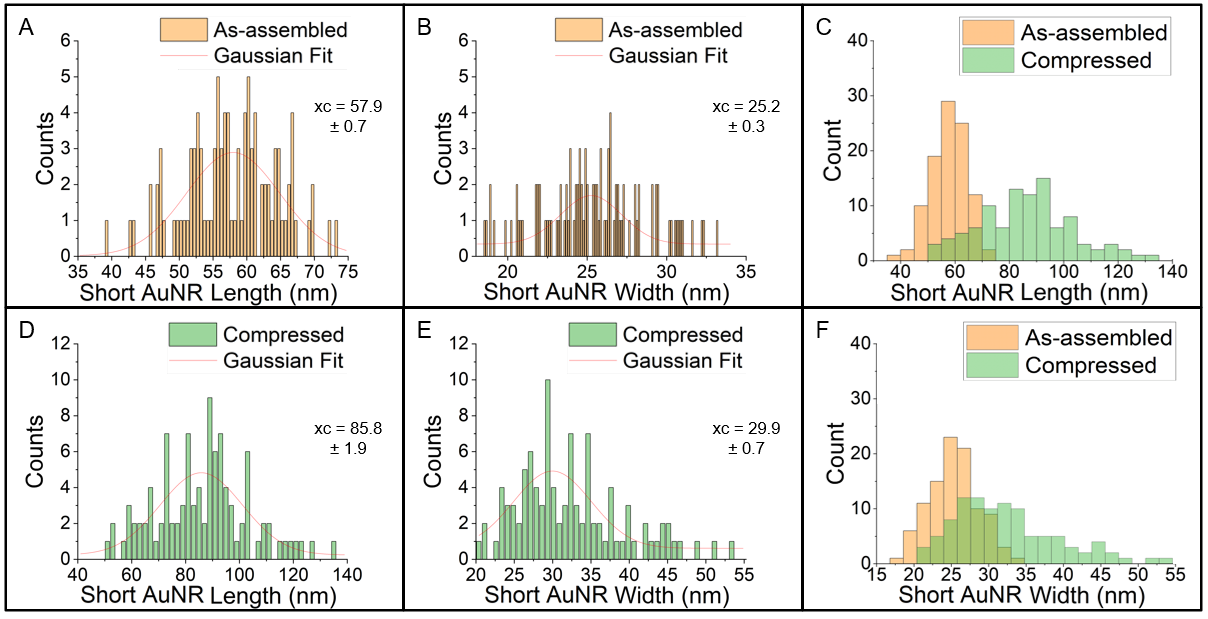
**

**Fig. S41**. A) - F) Statistical metrology analysis of as-assembled and compressed short AuNRs to understand their elongation along the length (major axis) and width (minor axis) via ellipse fitting of 100 AuNRs. As-assembled short AuNR has a length (major axis) and width (minor axis) of 57.9 ± 0.7 nm and 25.2 ± 0.3 nm, respectively. After compression, length, and width increased to 85.8 ± 1.9 nm and 29.9 ± 0.7 nm, respectively.

**
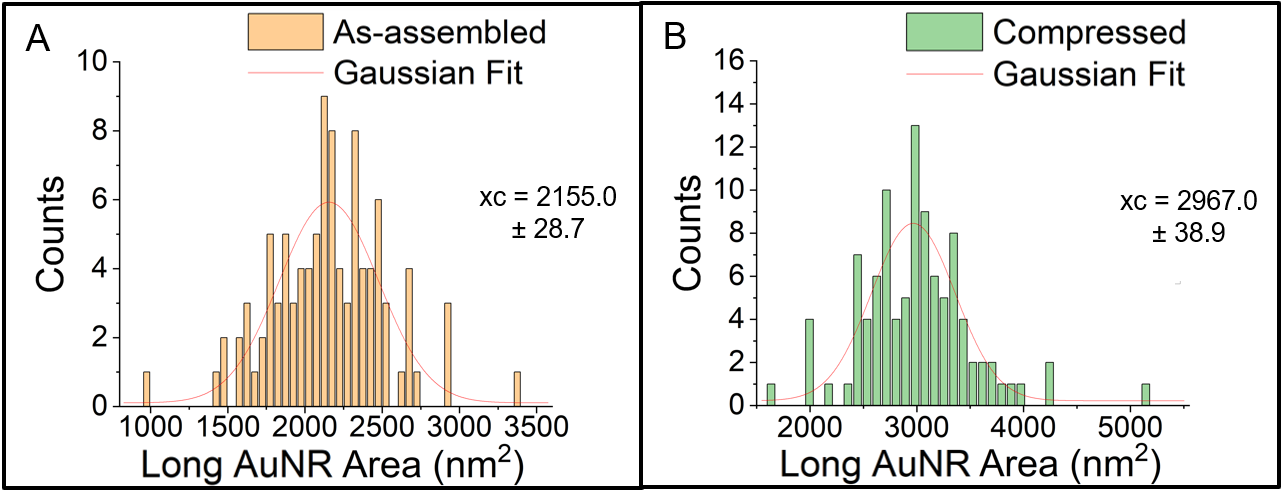
**

**Fig. S42**. Statistical metrology analysis of projected area as-assembled and compressed long AuNRs. A) The projected area of as-assembled long AuNRs was estimated to be 2155.0 ± 28.7 nm^2^. B) After compression, the area increased to 2967.0 ± 38.9 nm^2^ (a 37.68% change).


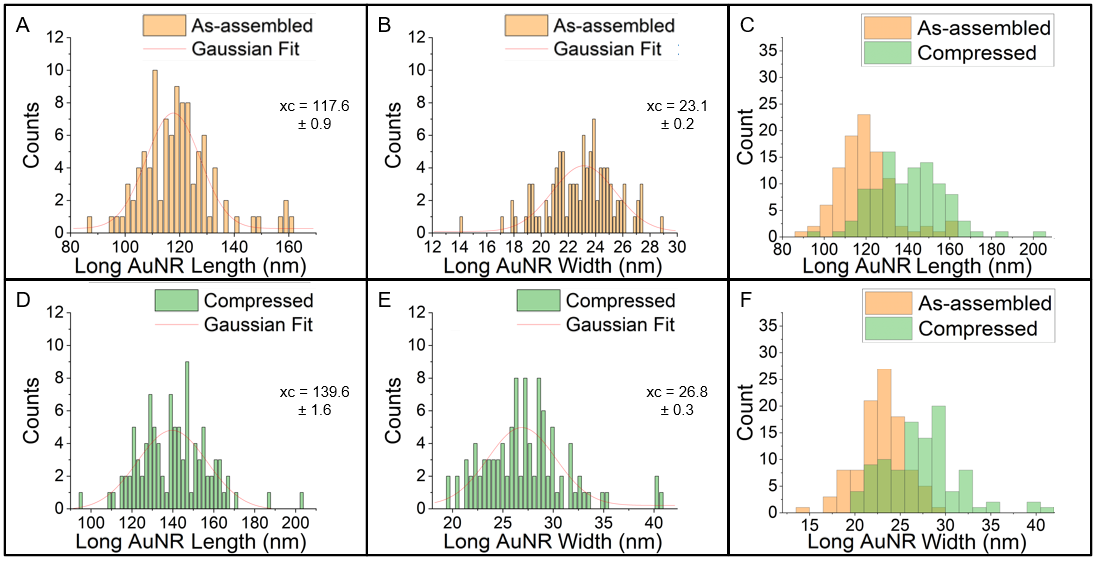


**Fig. S43**. A) - F) Statistical metrology analysis of as-assembled and compressed long AuNRs to understand their elongation along the length (major axis) and width (minor axis) via ellipse fitting of 100 AuNRs. As-assembled long AuNRs have a length (major axis) and width (minor axis) of 117.6 ± 0.9 nm and 23.1 ± 0.2 nm, respectively. After compression, length and width increased to 139.6 ± 1.6 nm and 26.8 ± 0.3 nm, respectively.

**Morphological anisotropy characterization and evolution of AuNR**

The morphological characterization of the AuNRs in the z-direction was characterized by methods similar to the AuNSs. For statistical analysis of the thickness evolution and understanding of the uniformity of the compression technique, ten AFM datasets across the sample were acquired and analyzed for each as-compressed and compressed AuNR sample (Figs. S44-47).

**
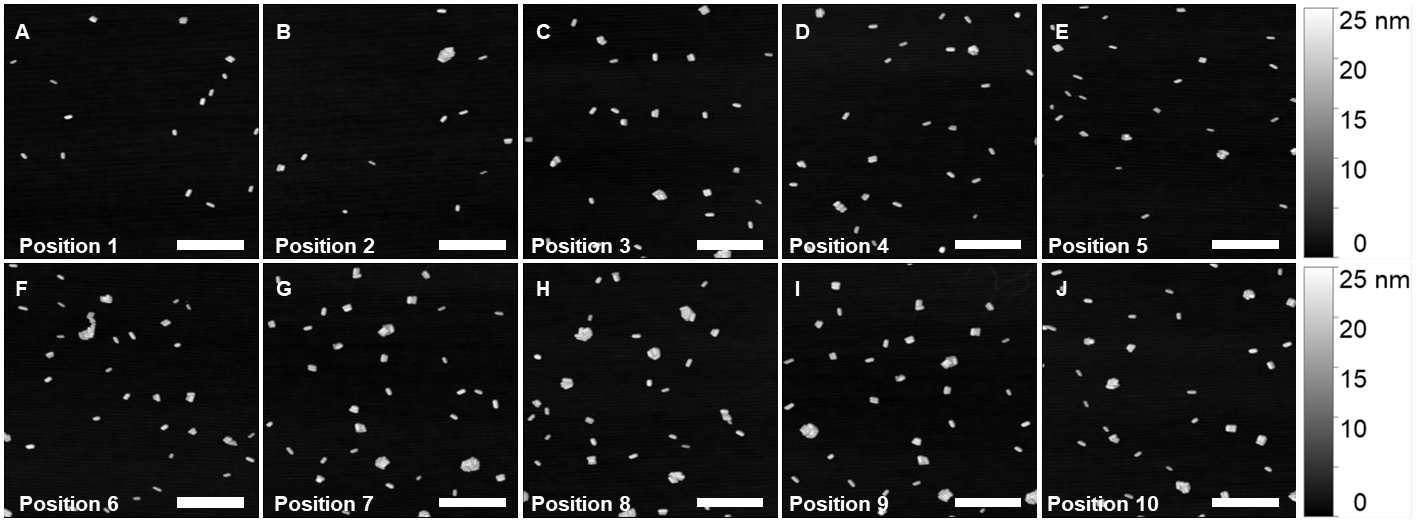
**

**Fig. S44.** A) - J) AFM maps of as-assembled short AuNR with 5k PEG. All scale bars are 520 nm.

**
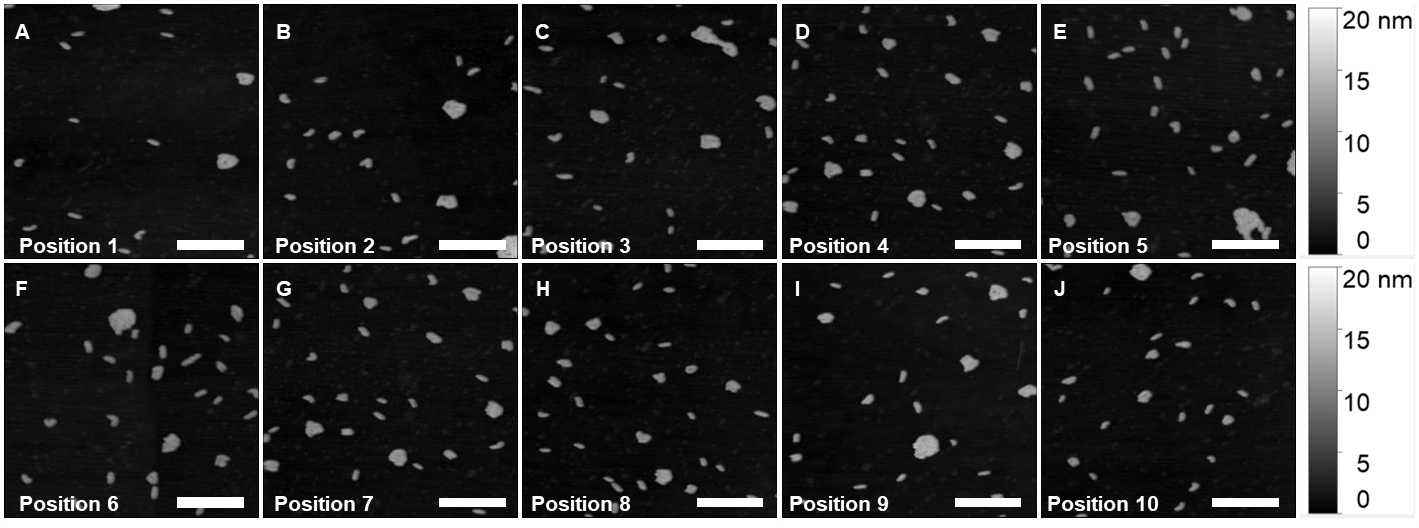
**

**Fig. S45.** A) - J) AFM maps of compressed short AuNR with 5k PEG. All scale bars are 520 nm.


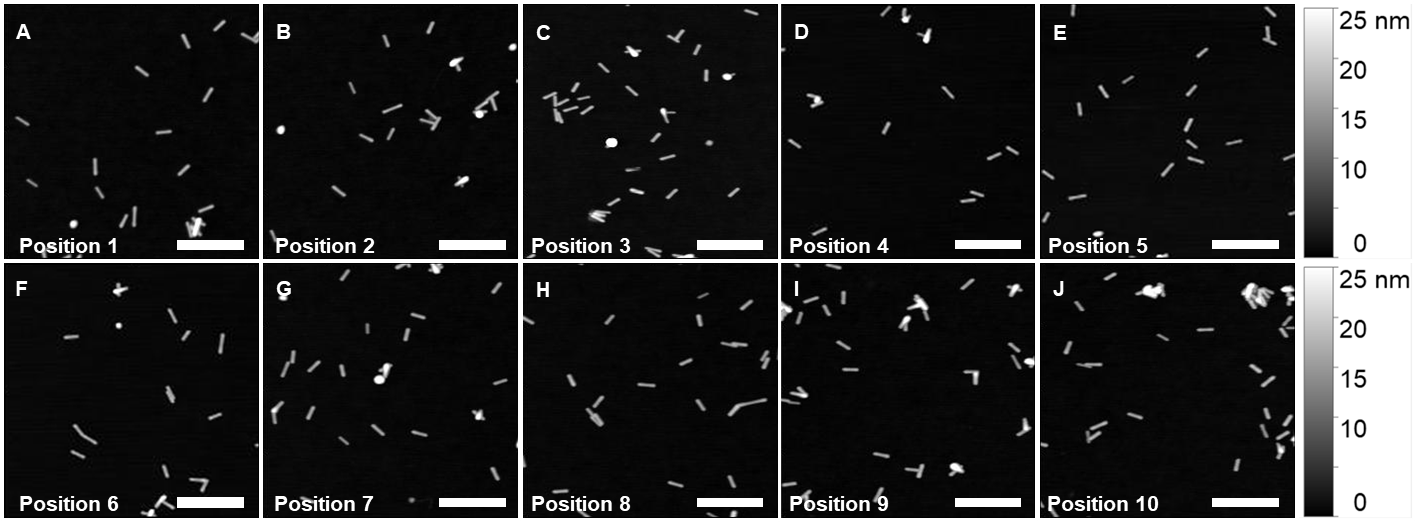


**Fig. S46.** A) - J) AFM maps of as-assembled long AuNR with 5k PEG. All scale bars are 520 nm.

**
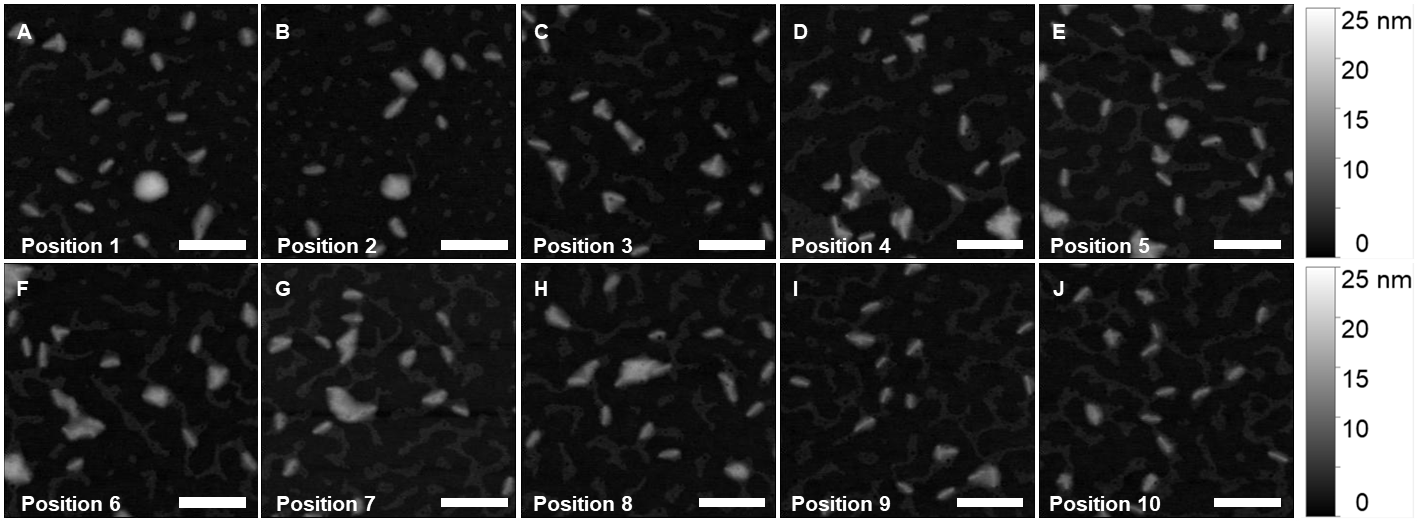
**

**Fig. S47.** A) - J) AFM maps of compressed long AuNR with 5k PEG. All scale bars are 520 nm.

**
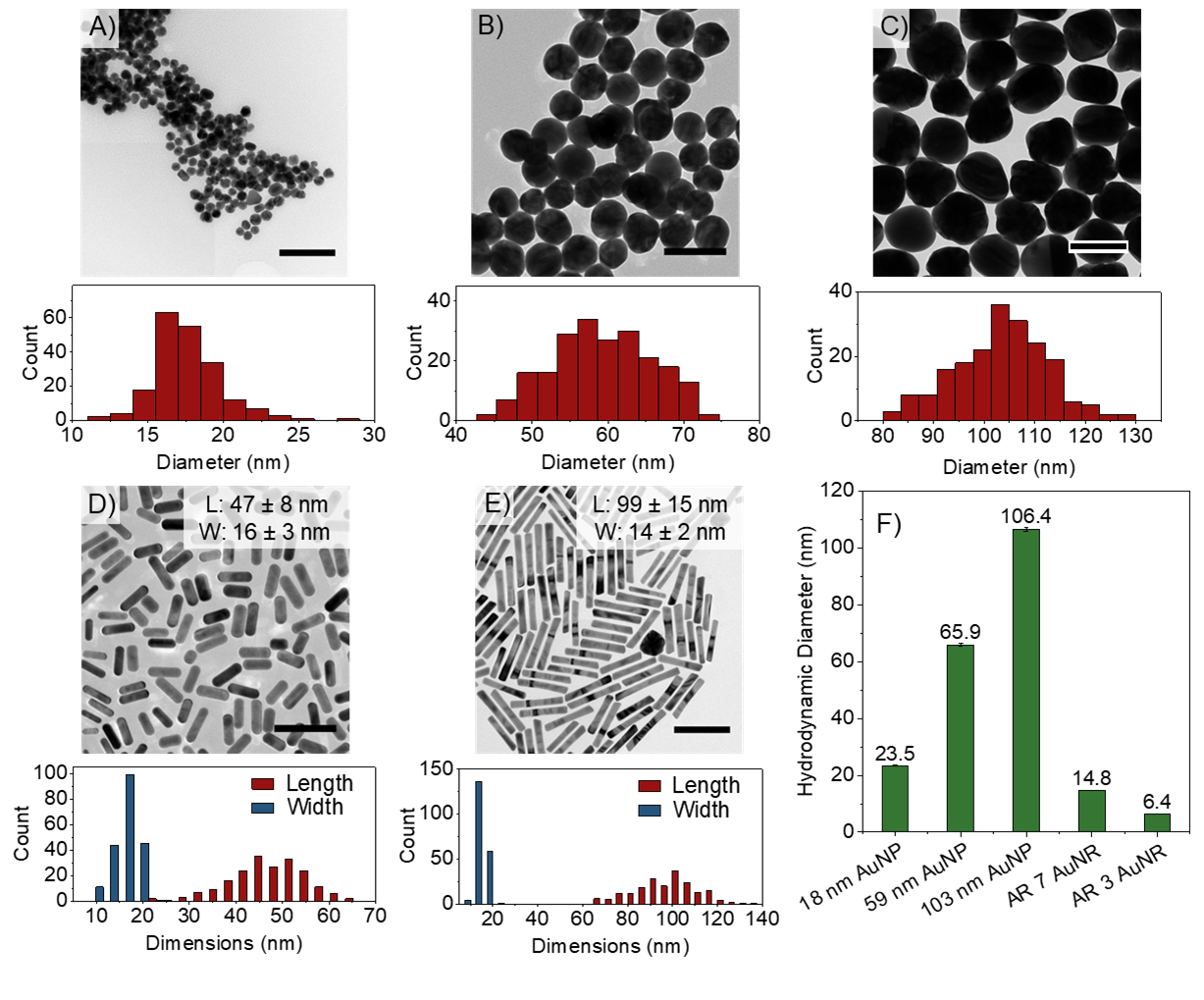
**

**Fig. S48.** BF**-**TEM micrographs of A) small (18 nm) AuNSs, B) medium (59 nm) AuNSs, C) large (103 nm) AuNSs, D) short AuNRs (60x20 nm and “AR 3”), E) long AuNRs (99x14 nm and “AR 7”), with corresponding size histograms. Each histogram contains data for 200 particles. All scale bars 100 nm. F) Hydrodynamic diameters were determined through dynamic light scattering.

**Effect of SAM on the top compression Si wafer**

Both the top Si wafer and bottom Si substrate are of the same bulk material and crystalline orientation. Therefore, the relative surface energy determines the relative binding of AuNPs to either surface after compression. The same cleaning processes were applied to both top and bottom Si wafers, including solvent cleaning, oxygen plasma cleaning, and piranha cleaning, all of which increase the surface energy of the native oxide surface and increase the interaction between the Si wafer and AuNPs during compression. With the bare native oxide surface, we found that a relatively high proportion of AuNPs become attached to the top Si wafer surface after compression, with the AuNP area coverage on the top Si wafer increasing from null to ~61% (Figs. S49 D, E, and F). To prevent this from occurring and to maintain the compressed nanoparticles on the bottom Si substrate, surface modification was performed to the top Si wafer surfaces with self-assembled monolayers (SAM) (trichloro (1H,1H,2H,2H perfluorooctyl), which reduces the surface energy significantly. For example, Wang et al. showed that the surface energy of oxide surfaces on Si wafers can be modified by using SAM which increases the water contact angle from 71.1° to 105.2° (2). Using this surface modification, the tendency of AuNPs to attach to the top Si wafer surface after compression is drastically reduced (close to null) (Figs. S49 A, B, and C). Consequently, nearly all the compressed AuNPs remain on the bottom Si substrate which facilitates the millimeter-scale uniformity of the compressed AuNP morphology and enables statistical characterization at distributed positions to quantify such uniformity.


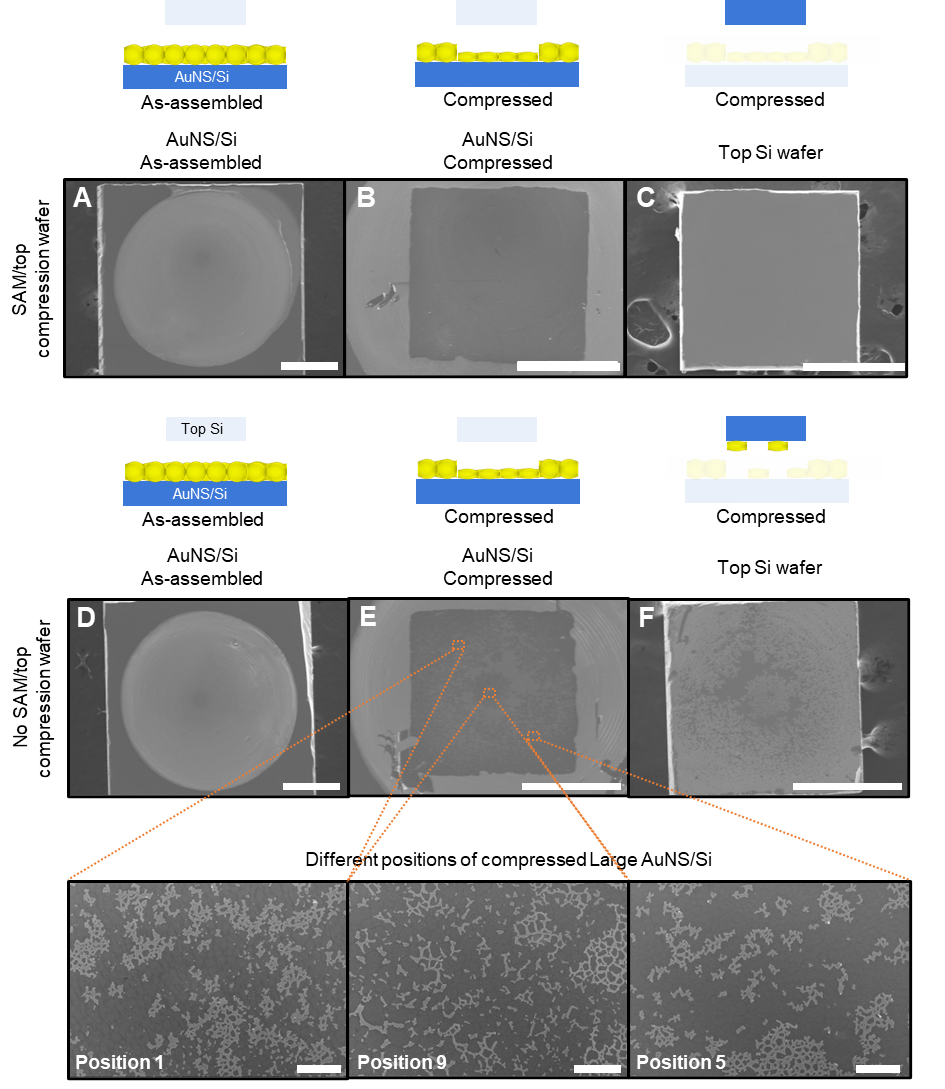


**Fig. S49.** A) As-assembled large AuNSs with 5k PEG on Si substrate, B) compressed large AuNSs with 5k PEG on the Si substrate (within the center square area), and C) SAM/top compression Si wafer after the compression process, showing no adhering particles. D) As-assembled large AuNSs with 5k PEG on the Si substrate, E) compressed large AuNS with 5k PEG on the Si substrate (within the center square area), and F) top compression Si wafer with no SAM after the compression process, with significant amounts of adhered particles. AuNSs were transferred onto the top Si wafer (area coverage of ~61% by AuNSs) after compression. Scale bars are 500 µm. Representative images (shown for positions 1, 9, and 5) of the particles remaining on the bottom Si substrate after a significant amount have been picked up by bare top compression Si wafer with no SAM. Scale bars are 10 µm.

**Area coverage calculation**

The area coverages of the AuNPs on the silicon substrates are statistically determined. The deposition parameters, such as nanoparticle concentration, deposition volume, and drying time, are optimized to achieve a deterministic and uniform area coverage of AuNP assembly on the silicon wafer. The area coverages of AuNPs are determined statistically by sampling thirteen positions distributed across the substrate within the central region (within the 0.6 mm × 0.6 mm area), as indicated by the black dots in Fig. S50. For each position, an SEM image is taken (at 20kx magnification with a field of view of ~28 um^2^) and processed using ImageJ software to perform binary conversion and area coverage calculations (Fig. S51). Considering all thirteen positions, the average area coverage, standard deviation (SD), and coefficient of variation (COV) for each sample are determined (Table S6). The uniform deposition of AuNPs on silicon substrate is confirmed when a sample has less than 30% COV.


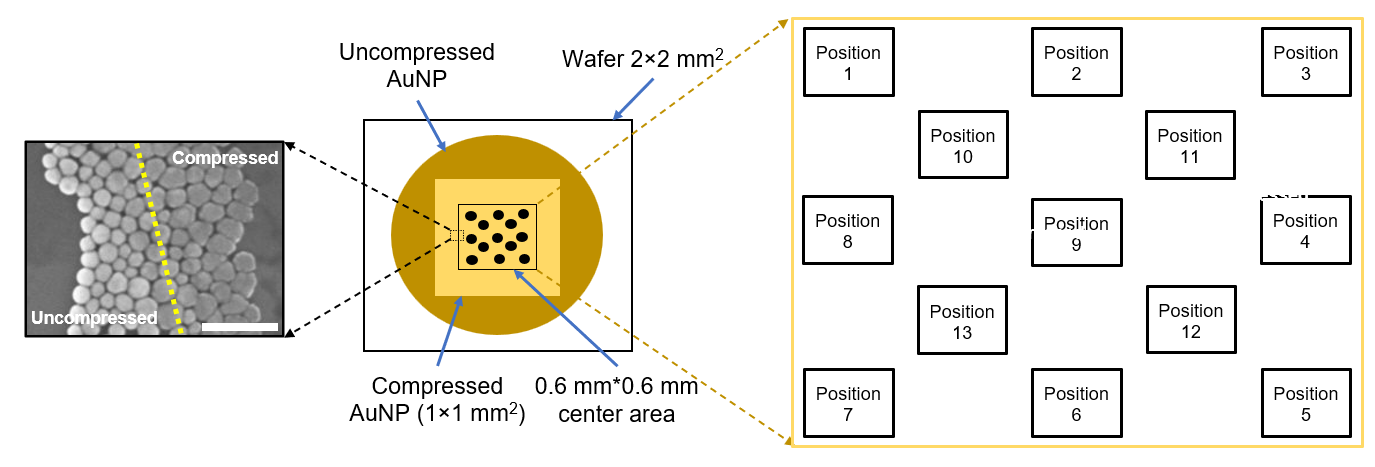


**Fig. S50.** Schematic of methodology for statistical calculation of AuNP area coverage on the silicon substrate. The left panel shows the boundary area of the as-assembled and compressed region of medium AuNP 5k PEG on silicon substrate. All scale bars are 250 nm.

**
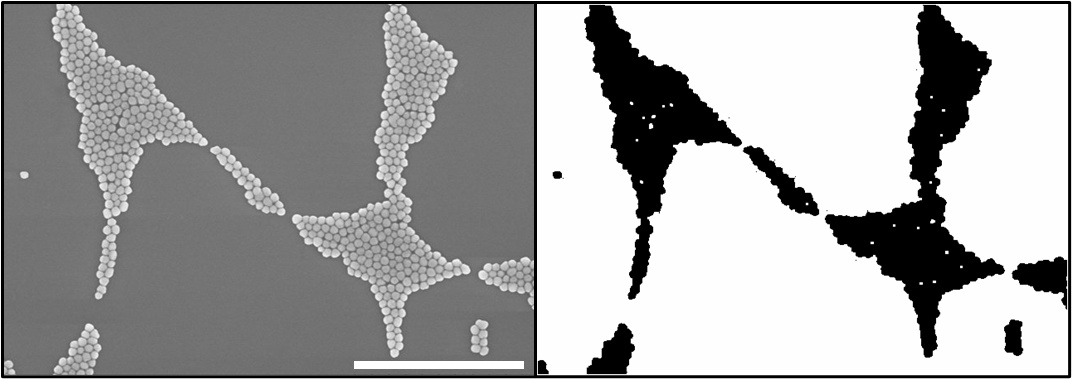
**

**Fig. S51.** Example area coverage quantification for assembled large AuNS 5k PEG, at position 12. The scale bar is 2 µm. The 20kx magnification SEM image is converted to binary, and the area coverage is calculated to be 19.11%.

**Table S1**: Summary of results from existing studies on the mechanical properties of gold at bulk, micro, and nanoscale:

| Research Group | Material | Study Type | Result | Computational Parameters | Remarks |
| --- | --- | --- | --- | --- | --- |
| Wang et al., *Journal of Physics: Condensed Matter (2010)* (3) | Single crystal Gold | Density functional theory calculation | Ideal compressive strength of 1.6 GPa | NA | Tensile properties were evaluated |
| Cerny et al., *Journal of Physics: Condensed Matter* (2009) (4) | Bulk Gold | Density functional theory calculation | Theoretical tensile strength:  1.8GPa [110] loading 3.2GPa [11-1] loading  3.6GPa [001] loading | NA | Orientation effect to the theoretical tensile strength. |
| Deng et al., *ACS Nano* (2009) (5) | Gold nanowire | Molecular Dynamics | UTS of 3.23 GPa | Temperature: 300 ± 2 K;  Diameter: 20 nm;  Strain rate: 2.7×10^7^/s | Tensile properties were evaluated |
| Wang et al., *Nature Communications* (2013) (6) | Gold nanowire | Molecular Dynamics | UTS of 5.5 GPa | Temperature: 300 ± 2 K;  Length: 68-270 nm  Strain rate: 1×10^8^/s | Tensile properties were evaluated |
| Mordehai et al., *Acta Materialia* (2011) (7) | Gold nanoparticles | Molecular Dynamics | The onset of dislocation nucleation ranged from ~3–4.2 GPa (particle diameter 24.1–17.5 nm) | NA | Quantitative information on the compressibility of particles in the nanoscale regime. |

| Research Group | Material | Study Type | Result | Experimental Parameters | Remarks |
| --- | --- | --- | --- | --- | --- |
| Islam et al., *Journal of Materials Research* (2017)(8) | Gold microparticle | Empirical solid-state compression | Compressive stress at which plastic yield occurs:  Case I: 0.99 ± 0.36 GPa to   2.5 ± 0.64 GPa  Case II: 0.17± 0.04 GPa to 0.34 ± 0.06 MPa | Temperature: 300K;  Sphere Diameter:  Case I: 0.8 µm,  Case II: 6 µm;  Loading rate: 0.01-1.0 mN/s | Quantitative information on the compressibility of particles in the micro regime. |
| Mordehai et al., *Acta Materialia* (2011) (7) | Gold microparticle | Empirical solid-state compression | The onset of plastic deformation in 199 MPa–7.6 GPa | Temperature: 300K | Quantitative information on the compressibility of particles in the micro regime. |
| Wang et al., *Nature Communications* (2013) (6) | Gold nanowire | In-situ TEM tension | UTS of 1.43-3.12 GPa | Diameter: 8-20 nm | Tensile properties were evaluated |
| Fang et al., *Nanoscale* (2020) (9) | Gold nanowires | AFM-based three-point bending test | Tensile Yield strength:  Case I: 0.8 ± 0.4 GPa to 1.9 ± 1.0 GPa;  Case II: 1.2 ± 0.3 GPa to 5.0 ± 2.4 GPa | Nanowire dimension:  Case I:  Width= 193-86 nm, Thickness: 228-60 nm  Case II:  Width: 170-97 nm  Thickness: 295-43 nm | Quantitative information on nanowire strength |
| Vogl et al., *MRS Advances* (2021) (10) | Gold nanowires | Empirical tensile test | UTS of  Wire I: 10.5 GPa  Wire II: 1.4 GPa  Wire III: 2.3  GPa | Cross section of different gold nanowires:  Wire I: 36 nm × 30 nm  Wire II: 96 nm × 100 nm  Wire III: 96 nm × 954 nm  Strain rate: 2-3×10^-4^/s | Quantitative information on nanowire strength |

**Table S2.** Tabulated data for the calculated IPS for the different sizes of AuNS with different PEG lengths.


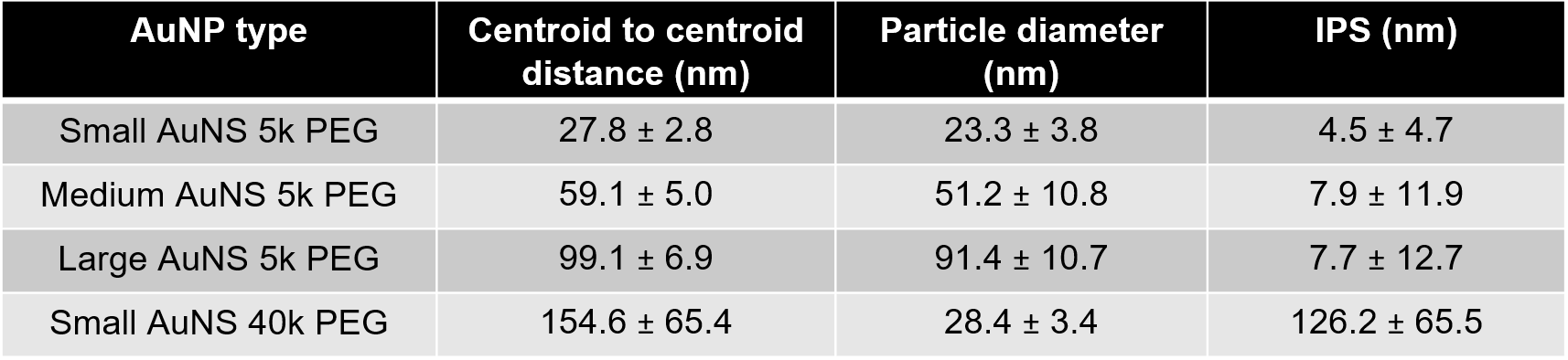


**Table S3.** Tabulated Schmid factor for compressive stress at different compression directions on the AuNP facets, considering the slip plane and slip direction as {111} and {101}, respectively. The Schmid factor ranges from 0.37 to 0.49.


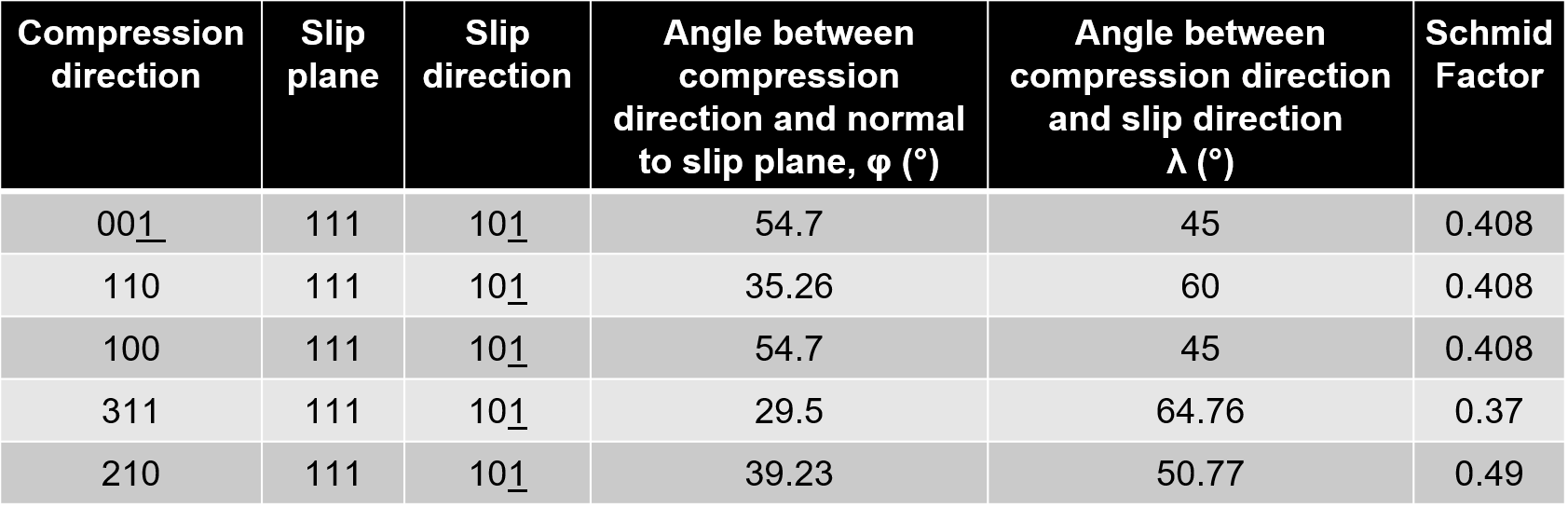


**Table S4.** AFM-extracted metrology data for as-assembled and compressed: small, medium, and large AuNSs with 5k PEG at different positions.


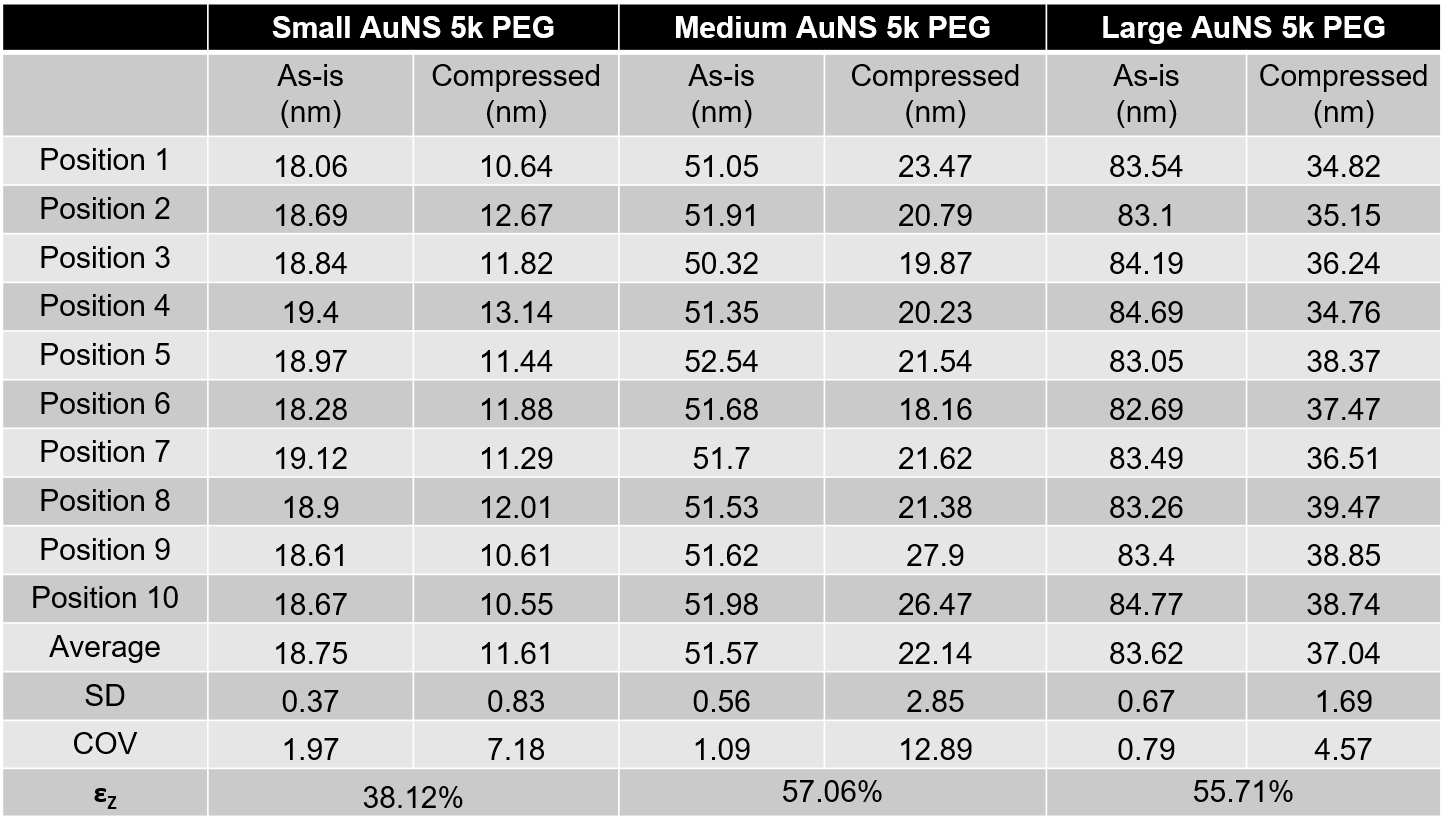


**Table S5.** Tabulated various compressed morphologies of small AuNSs for different initial equilibrium structures of the AuNSs with the corresponding true yield stresses at a compression velocity of 0.005 nm/ps and 0.001 nm/ps. The load is applied along [001] direction at 300K. All snapshots are taken at engineering strain of 38%.


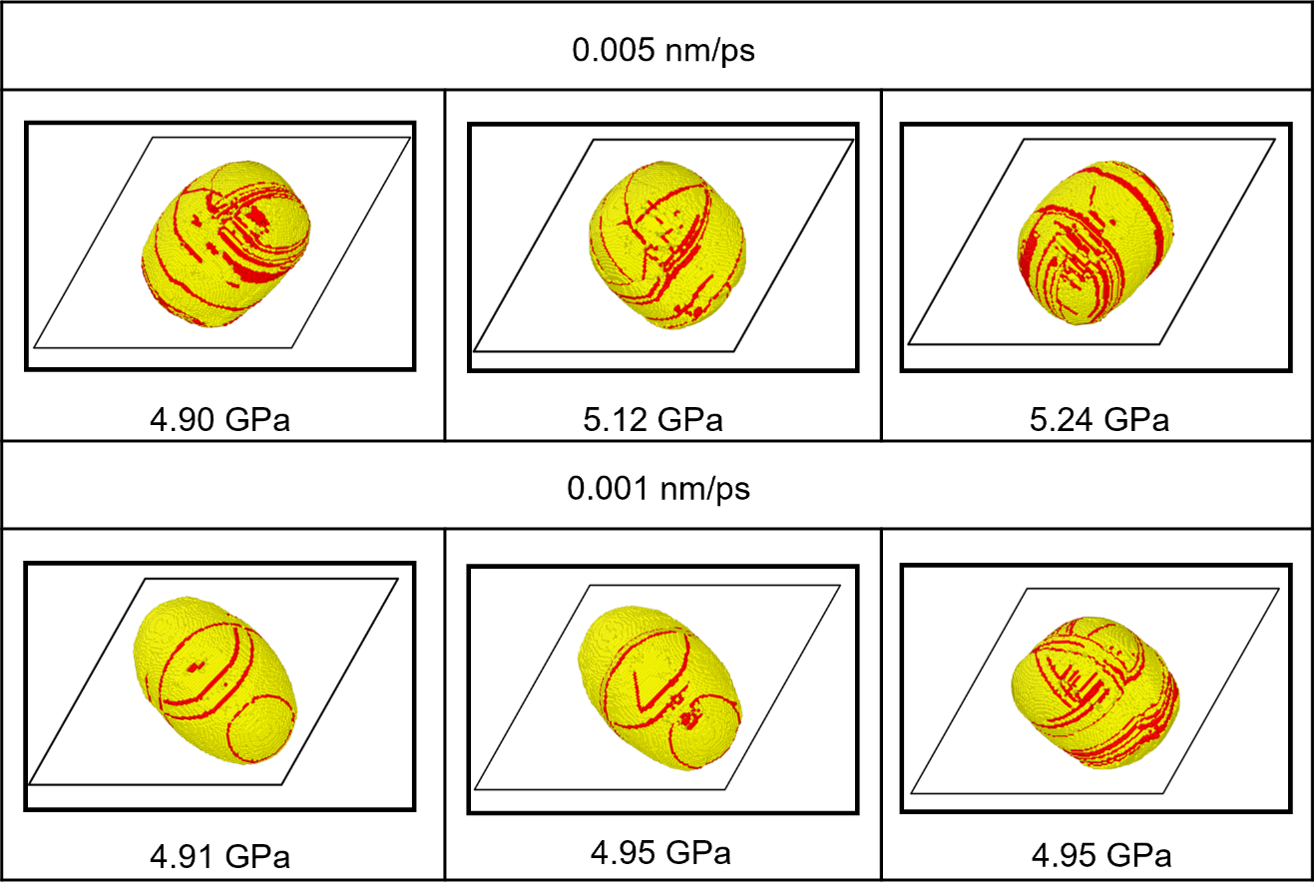


**Table S6.** Tabulated data for the calculated average area coverage of AuNP assembled on silicon substrates.

**
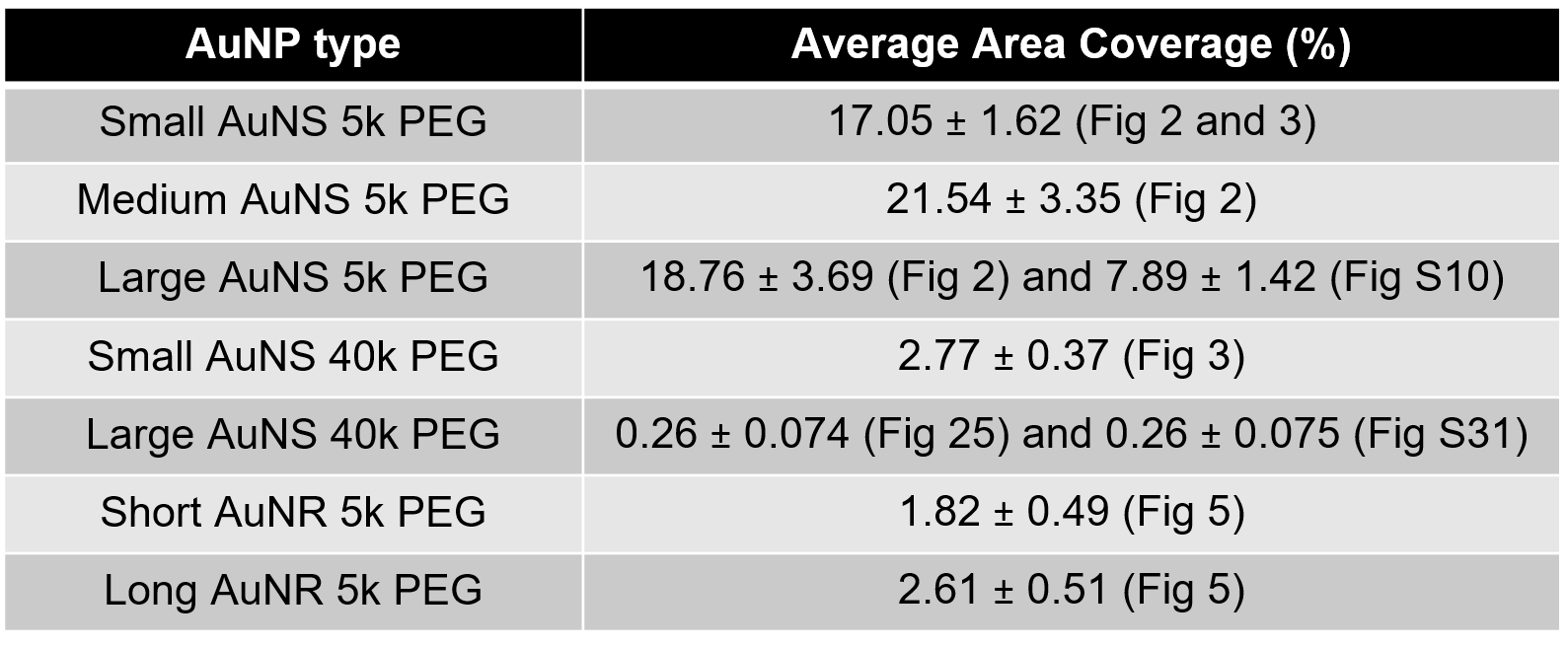
**

**SI References**

1. F. N. David, H. Cramer, Mathematical Methods of Statistics. *Biometrika* **34**, 374 (1947).

2. B. Wang, *et al.*, Support-free transfer of ultrasmooth graphene films facilitated by self-Assembled monolayers for electronic devices and patterns. *ACS Nano* **10**, 1404–1410 (2016).

3. H. Wang, M. Li, The ideal strength of gold under uniaxial stress: An ab initio study. *J. Phys. Condens. Matter* **22**, 5 (2010).

4. M. Ĉerný, J. Pokluda, The theoretical tensile strength of fcc crystals predicted from shear strength calculations. *J. Phys. Condens. Matter* **21**, 5 (2009).

5. C. Deng, F. Sansoz, Near-ideal strength in gold nanowires achieved through microstructural design. *ACS Nano* **3**, 3001–3008 (2009).

6. J. Wang, *et al.*, Near-ideal theoretical strength in gold nanowires containing angstrom scale twins. *Nat. Commun.* **4** (2013).

7. D. Mordehai, *et al.*, Size effect in compression of single-crystal gold microparticles. *Acta Mater.* **59**, 5202–5215 (2011).

8. A. A. Islam, R. J. Klassen, Kinetics of length-scale dependent plastic deformation of gold microspheres. *J. Mater. Res.* **32**, 3507–3515 (2017).

9. Z. Fang, Y. Geng, J. Wang, Y. Yan, G. Zhang, Mechanical properties of gold nanowires prepared by nanoskiving approach. *Nanoscale* **12**, 8194–8199 (2020).

10. L. M. Vogl, P. Schweizer, G. Richter, E. Spiecker, Effect of size and shape on the elastic modulus of metal nanowires. *MRS Adv.* **6**, 665–673 (2021).
